# Supplementary material for: Implementing communication and decision-making interventions directed at goals of care: a theory-led scoping review
Source: BMJ Open. 2017 Oct 6;7(10):e017056. doi: 10.1136/bmjopen-2017-017056 (PMC5640076; doi:10.1136/bmjopen-2017-017056)
Supplement: Supplementary data [file bmjopen-2017-017056supp001.pdf]

Appendix 1: ***Primary literature search strategy***

Combined key word searches		Number of results in MEDLINE	3rd key word added if results >200
End of life care	Goals of care	(298) 12	Treatment decision making
End of life care	Ceiling of care	5	
End of life care	Ceiling of treatment	1	
End of life care	Treatment limitations	12	
End of life care	Resuscitation	(545) 11	Treatment decision making
End of life care	DNACPR	(859) 79	Goals of care
End of life care	Treatment escalation plans	0	
End of life care	Treatment decision making	68	
End of life care	Advance care plans	28	
End of life care	Supported decision making	8	
End of life care	End of life decisions	(624) 4	Treatment limitations for Medline, goals of care for CINAHL
End of life care	Treatment goals	56	
Rapid response	Goals of care	11	
Rapid response	Ceiling of care	0	
Rapid response	Ceiling of treatment	0	
Rapid response	Treatment limitations	5	
Rapid response	Resuscitation	(239) 2	Treatment limitations or End of life decisions
Rapid response	DNACPR	(549) 5	Palliative care
Rapid response	Treatment escalation plans	0	
Rapid response	Treatment decision making	4	
Rapid response	Advance care plans	0	
Rapid response	Supported decision making	0	
Rapid response	End of life decisions	1	
Rapid response	Treatment goals	5	
Medical emergency	Goals of care	53	
Medical emergency	Ceiling of care	0	
Medical emergency	Ceiling of treatment	2	
Medical emergency	Treatment limitations	8	
Medical emergency	Resuscitation	(6,041) 11	End of life decisions
Medical emergency	DNACPR	(2,291) 7	End of life decisions or Treatment goals
Medical emergency	Treatment escalation plans	0	
Medical emergency	Treatment decision making	26	
Medical emergency	Advance care plans	0	

Medical emergency	Supported decision making	4	
Medical emergency	End of life decisions	22	
Medical emergency	Treatment goals	17	
Critical care outreach team	Goals of care	0	
Critical care outreach team	Ceiling of care	0	
Critical care outreach team	Ceiling of treatment	0	
Critical care outreach team	Treatment limitations	0	
Critical care outreach team	Resuscitation	2	
Critical care outreach team	DNACPR	0	
Critical care outreach team	Treatment escalation plans	0	
Critical care outreach team	Treatment decision making	0	
Critical care outreach team	Advance care plans	0	
Critical care outreach team	Supported decision making	0	
Critical care outreach team	End of life decisions	0	
Critical care outreach team	Treatment goals	0	
Palliative care	Goals of care	(633) 27	End of life decisions or Treatment decision making
Palliative care	Ceiling of care	4	
Palliative care	Ceiling of treatment	0	
Palliative care	Treatment limitations	34	
Palliative care	Resuscitation	(599) 54	Goals of care
Palliative care	DNACPR	(2,232) 84	Goals of care
Palliative care	Treatment escalation plans	0	
Palliative care	Treatment decision making	146	
Palliative care	Advance care plans	12	
Palliative care	Supported decision making	3	
Palliative care	End of life decisions	(359) 28	Treatment decision making or Goals of care
Palliative care	Treatment goals	182	
Clinical deterioration	Goals of care	5	
Clinical deterioration	Ceiling of care	0	
Clinical deterioration	Ceiling of treatment	0	
Clinical deterioration	Treatment limitations	1	
Clinical deterioration	Resuscitation	67	

Clinical deterioration	DNACPR	220	N/A (adding 3rd term gave 0 results)
Clinical deterioration	Treatment escalation plans	0	
Clinical deterioration	Treatment decision making	3	
Clinical deterioration	Advance care plans	0	
Clinical deterioration	Supported decision making	1	
Clinical deterioration	End of life decisions	0	
Clinical deterioration	Treatment goals	7	
Treatment escalation plans	--------------------	10	
Advance care plans	--------------------	57	
Treatment decision making	Resuscitation	49	
Treatment limitations	Resuscitation	25	
