# Supplementary material for: Implementing communication and decision-making interventions directed at goals of care: a theory-led scoping review
Source: BMJ Open. 2017 Oct 6;7(10):e017056. doi: 10.1136/bmjopen-2017-017056 (PMC5640076; doi:10.1136/bmjopen-2017-017056)
Supplement: Supplementary data [file bmjopen-2017-017056supp002.pdf]

Appendix 2: *Data extraction tool*

ARTICLE DETAILS				
Study Title				
Primary Author				
Year				
Country				
Setting	Community/Population		Nursing home/Residential care	
	Hospital/Secondary		General Practice/Primary care	
	Other (specify):			
Study design				
Participant(s)				
What is the research objective?				
Is the study describing a new/existing decision or communication tool?	Yes	Name of tool:	No	
What are the decisions of interest being studied? (e.g. DNACPR/active medical care/palliative care)	DNACPR		Other (specify):	
	Active Medical Care			
	Supportive/Palliative Care			
What are the core components of the tools/intervention being studied?	Escalation levels/interventions		Record of who plan discussed was discussed	
	Resuscitation status		Indication of person recording discussion and decision	
	Document rationale for treatment decision and resuscitation status		Mental capacity assessment	
	Review/discontinuation date		Prompt to record completion in notes (relating to separate tool only)	

<table>
<tr><td>Who makes decisions (e.g. consultant, junior doctors) and who is involved in decision-making (e.g. patients and families)?</td><td colspan="4"></td></tr>
<tr><td rowspan="2">What is the trigger to initiating the process of decision making and/or what factors are taken into consideration? (e.g in advance of deterioration/in crisis or emergency situation)</td><td>All patients within study setting</td><td></td><td>Selected patients within study setting</td><td></td></tr>
<tr><td colspan="4"></td></tr>
<tr><td rowspan="8">Coherence

Sense making work individually and collectively when faced with the problem of operationalizing an intervention</td><td colspan="4">1) Differentiation: Does the intervention differ, and if so, how is it different from current practices? What work has been undertaken to aid understanding of how the new intervention differs from current practice?</td></tr>
<tr><td colspan="4"></td></tr>
<tr><td colspan="4">2) Communal specification: Do staff have a shared understanding of the purpose of the intervention? How has a shared understanding of the purpose of the intervention been built among staff within the organisation?</td></tr>
<tr><td colspan="4"></td></tr>
<tr><td colspan="4">3) Individual specification: Do staff understand their specific tasks and responsibilities in relation to the intervention? What work has been done to ensure individual tasks and responsibilities are understood. Do individuals understand how the intervention will impact upon the nature of their work?</td></tr>
<tr><td colspan="4"></td></tr>
<tr><td colspan="4">4) Internalization: Do staff understand the potential value of the intervention for their work? What work has been done to promote understanding of the value, benefits and importance of the new intervention among staff and the organisation as a whole?</td></tr>
<tr><td colspan="4"></td></tr>
<tr><td rowspan="4">Cognitive participation

Relational work people do to build and sustain a community of practice around a new intervention</td><td colspan="4">1) Initiation: Are there key individuals identified as those driving the intervention forward?</td></tr>
<tr><td colspan="4"></td></tr>
<tr><td colspan="4">2) Enrolment: What work has been done to promote others to ‘buy-in’ and engage with the intervention, including training and education? Are people open to working with others in new ways to adopt the intervention?</td></tr>
<tr><td colspan="4"></td></tr>
</table>

	3) Legitimation: Do staff believe that participating in the intervention is a legitimate part of their role? What work has been undertaken to ensure people believe it is right for them to be involved with the intervention?
	4) Activation: Do people continue to support the intervention? What work has been undertaken to sustain the new intervention in practice and keep people supporting it's use?
**Collective action**<br><br>*The operational work that people do to enable new practices*	1) Interactional workability: Can the intervention be easily integrated into existing work practices? What work has been undertaken to allow the new intervention to integrate with existing staff and organisational practices?
	2) Relational integration: Do individuals feel confident in their own and others' abilities to use the intervention? What work has been undertaken to build knowledge and confidence in using the new intervention? Does the intervention impact upon working relationships and what work has been done to minimise this?
	3) Skill set workability: Has the work (associated with the intervention) been assigned to those with the most appropriate skills? What formed the decision behind how the division of labour was allocated? Has sufficient training given staff the skills to enable them to use the intervention?
	4) Contextual integration: What resources have been provided to support the new intervention in practice? Is the intervention adequately supported at managerial level?
**Reflective monitoring**<br><br>*The appraisal work that people do to assess and understand the ways a new intervention affects them and others around them*	1) Systemization: Has information been collected or are there plans to collect information to determine the usefulness of the intervention through feedback, audit or other means? Have the effects of the intervention been reported back to those involved?
	2) Communal appraisal: Is there communal agreement among staff as to the value of the intervention? Has the worth of the intervention been evaluated collaboratively in formal or informal groups?

	3) Individual appraisal: What is the effect of the intervention on an individuals' workload? Do individuals value the effect it has on their individual work?
	4) Reconfiguration: Has appraisal work lead to attempts to improve or modify the intervention?
Do patients and families understand the purpose of the intervention? What work has been done to increase their understanding?	
What is the work that patients have to do and do they involve others in the work (eg. family members)?	
Has feedback from patient/families been sought? Do they see the value of the intervention?	
What are the <u>types</u> of outcomes measured by the study?	
Other	
