# Supplementary material for: Implementing communication and decision-making interventions directed at goals of care: a theory-led scoping review
Source: BMJ Open. 2017 Oct 6;7(10):e017056. doi: 10.1136/bmjopen-2017-017056 (PMC5640076; doi:10.1136/bmjopen-2017-017056)
Supplement: Supplementary data [file bmjopen-2017-017056supp003.pdf]

Appendix 3: The constructs and sub-constructs of NPT [16]

Core Constructs		Sub-constructs	
**Coherence**	The work required to make sense of:<br>a) an intervention, both individually and collectively as a healthcare professional, clinical care team, service and/or organization.<br>b) what is required to individually and collectively implement the intervention (in this case a tool and/or process to support decision making regarding goals of care in the context of pathophysiological deterioration)	**Differentiation**	Understanding how a new set of practices are different from current practices
		**Communal Specification**	How people work together to build a shared understanding of the aims, objectives and expected benefits of a set of new practices
		**Individual Specification**	Work that will help individuals understand their specific tasks and responsibilities around a set of practices
		**Internalization**	Work to understand the value, benefits and importance of a set of practices
**Cognitive Participation**	The relational work required within a clinical care team, service and/or organization to establish and sustain the particular intervention	**Initiation**	Work to drive the new set of practices forward
		**Enrolment**	Organizing or reorganizing of participants in order to collectively contribute to the work involved in new practices
		**Legitimation**	The work of ensuring that participants believe it is right for them to be involved and that they can make a valid contribution
		**Activation**	Actions and procedures needed to sustain a practice and maintain participant involvement
**Collective Action**	The operational work of organising and preparing the care environment, such as a	**Interactional Workability**	The interactional work that people do with each other and with other elements of a set of practices, when they seek to operationalise them in everyday settings

	particular service or organisation, to enact the new practices required to deliver the intervention	**Relational Integration**	The knowledge work that people do to build accountability and maintain confidence in a set of practices and in each other as they use them
		**Skill set Workability**	The allocation work that underpins the division of labour that is built up around a set of practices
		**Contextual Integration**	Managing a set of practices through the allocation of different kinds of resources and the execution of protocols, policies and procedures
**Reflexive Monitoring**	The appraisal work that people do to assess the acceptability and utility of the intervention, as well as the outcomes of the intervention and its delivery on those who negotiate and enact it (clinicians, individual patients and their families/carers)	**Systemization**	The work of collecting information to determine the effectiveness and usefulness of a new set of practices
		**Communal appraisal**	Participants working together in formal collaboratives or in informal groups to evaluate the worth of a set of practices
		**Individual appraisal**	Participants in a new set of practices work as individuals to appraise its effects on them and the contexts in which they are set
		**Reconfiguration**	Appraisal work by individuals or groups may lead to attempts to redefine procedures or modify practices
