# Supplementary material for: Implementing communication and decision-making interventions directed at goals of care: a theory-led scoping review
Source: BMJ Open. 2017 Oct 6;7(10):e017056. doi: 10.1136/bmjopen-2017-017056 (PMC5640076; doi:10.1136/bmjopen-2017-017056)
Supplement: Supplementary data [file bmjopen-2017-017056supp004.pdf]

Appendix 4: *Summary of findings by NPT construct*

Core NPT Construct [16]	NPT Sub-Construct and Questions [16]	Key Findings	Identified Themes
Coherence	*Differentiation*<br>- Does the intervention differ, & if so, how is it different from current practices?<br>- What work has been undertaken to aid understanding of how the new intervention differs from current practice?	- High clarity & description of differentiation, & where relevant, explanation of aspects that were similar to or based upon existing practice: based upon existing national programmes of work [40, 44], frameworks [41] or guidelines [69]; established from the format of locally used tools [44], adapted from local trusts [46] or consisted of adaptations to pre-existing interventions [43, 45, 46]; entirely new interventions developed [1, 3, 31, 39, 50-52, 67], usually resulting from extensive scoping work [31, 36, 67].<br>- Interventions were developed to replace or complement existing practices or tools [1, 3, 33, 39, 41, 43, 46, 50-52].<br>- To understand how & why interventions should differ from current practices the following were drawn upon: case note reviews [46]; staff interviews [36]; scoping of published literature & guidelines [42, 67, 69]; review of local practices & contexts [31, 36, 47, 48, 61, 68].	Workability/functionality<br>Input into development
	Communal specification<br>- Do staff have a shared understanding of the purpose of the intervention?<br>- How has a shared understanding of the purpose of the intervention been built among staff within the organisation?	- Number of common themes highlighted as the key purposes of interventions: reducing unnecessary or inappropriate treatment [39, 44, 65]; enabling consideration of goals of care [31, 39, 42], guiding treatment planning & decision making [31, 39, 67, 71]; improving communication & discussions between patients & clinicians [31, 39, 40, 65, 71]; respecting patient wishes & ensuring appropriate care [31, 40, 44, 53]; improving the management of uncertainty & advanced planning [31, 65]; guiding smooth transitions of care out-of-hours & between different clinical settings [44, 46, 53, 62, 64]; improving documentation, accessibility & awareness of treatment plans [44, 46, 53, 62, 65]; providing uniformity & continuity of care [44, 53, 62, 64, 65].<br>- Background evidence & scoping work has the secondary purpose of informing staff as to the rationale & purpose of interventions [3, 31, 38, 42, 43, 46-48, 53, 67, 70].<br>- Multi-disciplinary collaboration in both development [3, 36, 46-48, 52] & education provision [42, 52, 68].<br>- Accessing alternative education programmes can act as a barrier to understanding purpose [69].<br>- Staff perception of purpose only minimally described [36, 62, 69], & some evidence of staff misunderstanding purpose [31, 39, 52].	Perceived value & appraisal<br>Input into development<br>Training & education
	Individual Specification<br>- Do staff understand their specific tasks & responsibilities in relation to the intervention? What work has been done to ensure they are understood?<br>- Do individuals understand how the intervention will impact upon the nature of their work?	- Importance of training [36, 48, 52, 68] & tailored education [36, 39] in facilitating staff understanding of tasks & responsibilities.<br>- Lack of training resulting in differing interpretations of an individuals' responsibilities for the same intervention [52].<br>- Lack of understanding of responsibilities or discomfort with proposed tasks leading to ineffective or reduced use [39-41, 54, 69].<br>- Methods of training delivery: incorporation into induction programmes [48]; simulated clinical scenarios [36]; presentations & meetings [68]; provision of material resources [36, 68].	Training & education<br>Perceived value & appraisal
	Internalization<br>- Do staff understand the potential value of the intervention for their work?<br>- What work has been done to promote understanding of the value, benefits &	- Importance of implementation staff understanding the value of an intervention [36, 39, 42, 46].<br>- Foreseen advantages included prompting consideration of more forms of treatment & reducing the negative connotations of DNACPR [36, 46].<br>- Foreseen disadvantages included potential for staff confusion, committing patients to overtreatment & potential patient distress [36].<br>- Implementation area appears important in promoting value, & more challenging in specialities less used to practicing the concepts involved in the intervention [45, 69].	Perceived value & appraisal<br>Setting & context

	importance of the new intervention among staff & the organisation as a whole?	- Staff surveys [36, 46, 62, 71] & collaborative development [36, 38, 65, 67, 69] generated consensus as to the intervention's importance, & promoted value, often based on problems encountered in current practice [46, 71].<br>- National guidance & policy were drawn upon to highlight current inadequacies in practice interventions were designed to address [3, 41, 65]. Established local policy influenced the value attributed to an intervention [41].	Input into development
Cognitive Participation	*Initiation*<br>- Are there key individuals identified as those driving the intervention forward?	- Majority of interventions piloted or implemented within limited settings or specific services [32, 44, 46, 60, 61, 63], often with input into the design from staff within these settings [44, 46, 63].<br>- Key clinical proponents [41, 54, 69] or multidisciplinary teams [68] were used to liaise & orchestrate implementation over multiple sites.<br>- Interventionist/facilitator contact important in orientating & maintaining momentum of local leadership [41, 68, 69].<br>- Input into design & development from: specialists within relevant specialties [31, 41, 46, 67] & multidisciplinary contributors, gaining collective knowledge from all potential users [31, 36, 47, 48, 62].<br>- Senior clinicians or management responsible for compliance with new interventions [54, 64, 69].	Key clinical proponents<br>Input into development<br>Setting & context
	Enrolment<br>- What work has been done to promote others to 'buy-in' & engage with the intervention, including training & education?<br>- Are people open to working with others in new ways to adopt the intervention?	- Training & education encouraged 'buy-in' [3, 31, 36, 41, 42, 48, 53, 54, 68, 69]: incorporated into established working practices [42, 48, 68, 69]; led by senior clinicians from relevant specialties [31, 41, 69]; consisted of multiple modalities [31, 41, 42, 68, 69].<br>- Timeliness of training delivery: prior to implementation [3, 36]; alongside implementation [41, 42, 53, 54, 68, 69]; repeated training [31, 69].<br>- Participation in development shown to promote 'buy-in' [36, 41, 45, 46, 48, 67]. Different techniques were adopted: anonymity of input [45, 67]; individual input in isolation, or prior to group consultation [36, 45, 46, 67]; actively involving those opposed to the intervention [36]; seeking opinions from staff experienced in the subject area targeted by the intervention [31, 36, 46, 67]; input into the intervention design [46, 47, 69] & implementation process [41, 46] from staff/specialists working within implementation settings.<br>- Difficulty in transforming clinician attitudes can act as a barrier to engagement [45].	Workability/functionality<br>Key clinical proponents<br>Training & education<br>Perceived value & appraisal<br>Input into development<br>Setting & context
	Legitimation<br>- Do staff believe that participating in the intervention is a legitimate part of their role?<br>- What work has been undertaken to ensure people believe it is right for them to be involved with the intervention?	- Legitimation was important, but its extent among clinicians varied [43, 61, 69].<br>- Facilitators of legitimation: prior use of the intervention [41]; perceived usefulness of the intervention [60, 61]; opportunities to provide input or suggest modifications to the intervention, irrespective of seniority [36, 46, 47, 67].<br>- Legality appeared as both a barrier & facilitator to legitimation [45, 46]: participation related to a sense of legal duty as opposed to behaviour change [45] & concern for potential legal ramifications were a barrier for clinicians [46].	Perceived value & appraisal<br>Input into development<br>Setting & context
	Activation<br>- Do people continue to support the intervention?<br>- What work has been undertaken to sustain the new intervention in practice & keep people supporting its use?	- Evidence of diffusion of uptake & cultural change across settings [1, 31, 36, 68, 69].<br>- Follow-up resources (including guidelines & policy), & mentorship facilitated implementation & continued support of interventions [34, 42, 47, 53, 64, 65, 68, 69].<br>- Consideration given to specialty & robustness of implementation site [41, 69].<br>- Some evidence of poor completion or failure to honour interventions [40, 52].	Setting & context<br>Key clinical proponents<br>Training & education<br>Perceived value & appraisal
Collective action	*Interactional Workability*<br>- Can the intervention be easily integrated into existing work practices?<br>- What work has been undertaken to allow the new intervention to integrate with existing staff & organisational practices?	- Ability to integrate with existing working practices recognised as important [31, 36, 68].<br>- Facilitators to integration: integration as part of, or in conjunction with, existing practices [3, 31, 38, 39, 46, 64, 68]; usefulness, pragmatism & accessibility of intervention [1, 3, 36, 44, 47-49, 60, 64, 65, 71]; involvement of users in design & piloting, allowing for amendments to facilitate utility [36, 41, 46, 47]; format of intervention based on existing design [44, 46]; transferability across healthcare settings [40, 44, 64].<br>- Barriers to integration: logistical & staffing barriers including staff turnover, management changes & staff availability to complete interventions [39-41, 44]; replication of existing work [47, 69]; clinical complexity [32, 46].	Workability/functionality<br>Input into development<br>Setting & context<br>Training & education

		- Interactional workability of training was an important consideration [69, 71].<br>- Electronic medical health records (EMR): for some, configuration of interventions within EMR facilitated implementation [42, 68], whilst others described poor navigation of interventions within EMR [36, 52].	
	Relational Integration<br>- Do individuals feel confident in their own & others' abilities to use the intervention?<br>- What work has been undertaken to build knowledge & confidence in using the new intervention?<br>- Does the intervention impact upon working relationships & what work has been done to minimise this?	- Circulation of interventions during development/early implementation appeared important for building confidence in their use [31, 36, 44, 52, 62] & allowed for amendments to facilitate understanding [52].<br>- Low confidence in particular aspects of interventions: capacity decisions & determining goals of care [39, 52].<br>- Reduced confidence in others: the ability of clinicians from other specialties to make decisions [36]; potential prematurity of decisions [36]; evidence of interventions not being followed due to disagreement by on-call clinicians [46].<br>- Increased confidence in others: clinicians keen to involve other clinicians & nurses expressed their confidence in others [36].<br>- Evidence of better teamwork & communication especially out-of-hours, driven by improved clarity & consistency of decisions [3, 31, 38, 44].<br>- Multi-modal format of interventions consisting of tools, prompts, algorithms & guidelines, invoke confidence in their application [1, 3, 31, 32, 36, 39, 40, 44, 48, 61-63, 67-70].<br>- Multidisciplinary educational programmes help build knowledge & confidence across disciplines [68, 69].	Workability/functionality<br>Input into development<br>Perceived value & appraisal<br>Setting & context<br>Training & education
	Skill set Workability<br>- Has the work (associated with the intervention) been assigned to those with the most appropriate skills?<br>- What formed the decision behind how the division of labour was allocated?<br>- Has sufficient training given staff the skills to enable them to use the intervention?	- Division of work based on level & nature of responsibility, among nurses, senior & junior clinicians [1, 3, 31-33, 36, 39-46, 67-71].<br>- Delegation often controlled for as the minimum level of clinicians permitted to complete or authorise was specified [1, 3, 36, 42, 46, 48, 62-65, 69].<br>- Colleagues should delegate if they do not have adequate skills [1], but concern was apparent if staff delegated to lacked the necessary experience/skills [42].<br>- Training & education was utilised [31, 39, 41, 68, 69], proposed [70, 71], or identified as deficient [40, 45], in facilitating staff development of skills, & application of skills to the processes involved in using interventions. The following were identified as important: skills training [39, 40, 42, 45, 52, 68, 69, 71]; communication skills (highlighted as difficult, important to clinicians & requiring development) [31, 33, 39, 45, 71]; other skills including: assessment & shared understanding of goals of care [1, 31, 33, 39, 45, 71]; training in application of skills to intervention [40, 52, 54, 64, 68, 69]; having someone in a position of responsibility for training execution/attendance [64, 68, 69]; different educational materials used alongside training programmes [68, 71]; value of not over-assuming existing skillset [52].<br>- Different types of training to teach relevant skills [31, 33, 68, 69]: teaching rounds [31, 68, 69] & formal group sessions [31, 69].<br>- Consideration of experience versus skill, & the interplay of external patient & organisational factors [42, 44].	Setting & context<br>Workability/functionality<br>Training & education<br>Key clinical proponents

	Contextual Integration<br>- What resources have been provided to support the new intervention in practice?<br>- Is the intervention adequately supported at managerial level?	- Patient & carer information leaflets are important [35, 36, 42, 51, 55, 66] & encourage shared communication [38, 64].<br>- A variety of staff guidance resources & updates used: newsletter updates, frequently asked questions documents, briefing notes, guidebooks, pocket cards, checklists, guidelines, frameworks, DVDs, prompt lists, algorithms, procedural leaflets [36, 42, 56-59, 64, 65, 68-70].<br>- A number of interventions also functioned as guidelines, with the use of flowcharts, prompts & checklists [1, 3, 31, 32, 36, 39, 40, 42, 44, 48, 61-63, 67-70], described as encouraging full & accurate completion [31, 42, 70].<br>- Joint patient & staff resources (poster & brochure) [36, 42].<br>- Guidelines incorporated in trust policy documents [36, 42, 64, 65, 68-70].<br>- Evidence from training scenarios used to shape information guidance, in order to address misconceptions evident during training [36].<br>- Guidance was distributed: alongside training sessions [71]; in staff inductions or updates [56, 64]; in local policy documents [65]; during or alongside implementation [36, 57-59, 68, 69]; as part of the intervention itself [69, 70].<br>- Websites: UFTO intervention has its own website providing access to resources, background to development, guidance for use & updates [37, 38]. One study refers to a palliative care website as a resource for implementation [69].<br>- Policy & protocol: three intervention specific policy documents [53, 64, 65]; one end of life care policy document referring to the intervention [34]; most relate to resuscitation & escalation planning [42, 47, 53, 64, 65], but also include trust induction policies [64] & EOLC policies [34].<br>- Evidence of managerial acceptance: approval from heads of department & medical directors [48, 62]; nationwide implementation [31]; incorporation into policy & protocol [34, 42, 47, 53, 64, 65].<br>- Managerial support acts as a catalyst to instigating & maintaining implementation [41, 68, 71].<br>- Levels of contact with management, & influence of management culture recognised [41, 69].	Training & education<br>Workability/functionality<br>Perceived value & appraisal<br>Key clinical proponents
Reflexive Monitoring	*Systemization*<br>- Has information been collected or are there plans to collect information to determine the usefulness of the intervention through feedback, audit or other means?<br>- Have the effects of the intervention been reported back to those involved?	- All describe or propose the collection of information relating to measures of usefulness [1, 3, 31-33, 38-43, 45-52, 54, 60-63, 68, 69].<br>- Data collection is proposed in studies where interventions have not yet been implemented [36, 44, 67, 70, 71].<br>- Different data collection methods: medical record audit [32, 39-41, 43, 46, 60, 61, 63, 68, 69], in the majority executed pre & post implementation, or in comparison with control groups [41, 43, 60, 61, 63, 68, 69]; staff surveys or interviews, often used in combination with medical record audit [3, 40, 41, 46-48, 50, 60, 61, 63, 69]; direct ward based observation [3]; patient interviews [1, 51].<br>- Different outcomes assessed: clinical impact of interventions (e.g. patient deaths) [3, 31, 32, 61]; clinical factors (e.g. illness trajectory) related to the use of interventions [32]; changes in end of life care processes, behaviours & outcomes (e.g. opioid prescriptions) [45, 46, 68, 69]; rates of completion, adherence & compliance with new interventions [31, 39-41, 43, 45, 46, 61, 69], & specifically with regard to documentation [39, 47, 48, 63]; new staff knowledge of care principles associated with the intervention [69]; attitudes toward the intervention [40, 63]; perceived effects of the intervention on practice [41, 50]; practicalities of the intervention [60, 61].<br>- Specific measures: study specific set of evaluation tools to measure feasibility [69] & validated measure to assess harms [3].<br>- Patient & family feedback outcomes are limited in scope & number [1, 3, 31, 33, 40, 47-49, 51, 62, 69, 71]: many recommend or propose the measure of patient & family experience [3, 31, 40, 47, 48, 62, 69, 71], some include measures of patient & family experience [1, 33, 49, 51]. Although limited, feedback was positive & demonstrated preferences for new interventions over previous processes.	Perceived value & appraisal<br>Training & education

		- Reporting the effects of the intervention to those involved: research publication [1, 3, 31-33, 38-43, 45-47, 49-51, 54, 60, 68, 69]; shared on intervention website [3, 38]; results communicated to promote shared learning [54]; shared with consultant panels for improved insight [47].	
	Communal appraisal<br>- Is there communal agreement among staff as to the value of the intervention?<br>- Has the worth of the intervention been evaluated collaboratively in formal or informal groups?	- One study evaluated staff perceptions in a collaborative setting, including focus groups & leadership meetings [69].<br>- Perceived value was evaluated via interviews, audits & questionnaires [3, 40, 41, 46-48, 50, 60, 61, 63, 69].<br>- The importance of individual & group methods in gauging attitudes was described, yet their relative efficiency was not explored [40, 41, 69].<br>- Communal staff appraisal may allow for a greater demonstration of impact than medical record audit data [69].<br>- Many demonstrated communal agreement as to the high level of value that staff attributed to an intervention [3, 40, 41, 47, 48, 50, 60-62, 69].<br>- Assessment of worth related to the intended (& perceived) purpose of interventions [31, 36, 39, 40, 42, 44, 46, 53, 62, 64, 65, 67, 69, 71].<br>- Only one study described negative staff perceptions [40].	Perceived value & appraisal
	Individual appraisal<br>- What is the effect of the intervention on an individuals' workload?<br>- Do individuals value the effect it has on their individual work?	- High value attributed to the intervention by staff, due to positive impact on working practices: reduces negative associations with DNACPR due to its universal application [3] or focus on positive treatment actions [50]; prevents unwanted resuscitation or escalation of care & ensures appropriate level of care [40, 44, 46, 47, 50]; provides a mechanism for forward planning: assessing, communicating & honouring patient preferences [3, 40]; increases staff confidence with, & improves clarity of, patient care plans [40, 44, 47, 50, 62]; improves team working & communication, especially out-of-hours [33, 46, 62]; improves ease & motivation of decision-making processes [44, 47, 60-62].<br>- Perceived & noted increases in workload were a concern for staff. However, it was generally perceived to be a worthwhile investment due to clarity, patient benefit & time saved later on [1, 3, 44, 60, 61, 69].	Workability/functionality<br>Perceived value & appraisal<br>Setting & context
	Reconfiguration<br>- Has appraisal work lead to attempts to improve or modify the intervention?	- Importance of appraisal & reconfiguration emphasised by evidence of its occurrence [31, 32, 36, 39, 41, 42, 46, 47, 51, 61, 62, 69].<br>- Reconfiguration part of the implementation process: adoption of PDSA (plan, do, study, act) cycles [46, 47]; piloting the intervention prior to roll out (with feedback, evaluation & subsequent amendments) [36]; early testing & adaptations [31]; use of sequential role out to amend implementation processes based on gathered insight [69].<br>- Appraisal & feedback led to suggested [69] & actual modifications [31, 36, 39, 46, 47, 61]: refinement of identification or trigger questions [31, 69]; ease of use, layout & clarity [36, 47]; inclusion & clarity of review dates [36, 46]; inclusion of free text boxes [36, 46].<br>- Appraisal led to further study or proposed new areas of study [32, 39, 61, 62, 69]: additional research to determine impact on specific outcomes [39]; feasibility of interventions over longer timeframes [69] & across different settings [32, 61]; studies to address distinct but associated problems identified during the appraisal work (e.g. lack of clinician presence at family meetings) [32, 62, 69].<br>- Appraisal also led to recognition of the need for, & subsequent development of, new patient information materials & services [51, 69], proposals for staff support services [41] & the revision & inclusion of interventions in local & trust policy [42, 47, 61].<br>- In two studies, appraisal demonstrated no desired impact, leading to the proposal or development of alternative interventions [43, 45].	Perceived value & appraisal<br>Input into development<br>Workability/functionality<br>Setting & context<br>Training & education
