# Supplementary material for: Implementing communication and decision-making interventions directed at goals of care: a theory-led scoping review
Source: BMJ Open. 2017 Oct 6;7(10):e017056. doi: 10.1136/bmjopen-2017-017056 (PMC5640076; doi:10.1136/bmjopen-2017-017056)
Supplement: Author's manuscript [file bmjopen-2017-017056.draft_revisions.pdf]

BMJ Open

BMJ Open is committed to open peer review. As part of this commitment we make the peer review history of every article we publish publicly available.

When an article is published we post the peer reviewers' comments and the authors' responses online. We also post the versions of the paper that were used during peer review. These are the versions that the peer review comments apply to.

The versions of the paper that follow are the versions that were submitted during the peer review process. They are not the versions of record or the final published versions. They should not be cited or distributed as the published version of this manuscript.

BMJ Open is an open access journal and the full, final, typeset and author-corrected version of record of the manuscript is available on our site with no access controls, subscription charges or pay-per-view fees (http://bmjopen.bmj.com).

If you have any questions on BMJ Open's open peer review process please email info.bmjopen@bmj.com

Implementing communication and decision-making interventions directed at goals of care: a theory led scoping review

Journal:	*BMJ Open*
Manuscript ID	bmjopen-2017-017056
Article Type:	Research
Date Submitted by the Author:	04-Apr-2017
Complete List of Authors:	Cummings, Amanda; University of Southampton Faculty of Health Sciences,<br>Lund, Susi; University of Southampton,<br>Campling, Natasha; University of Southampton Faculty of Health Sciences<br>May, Carl; University of Southampton, Faculty of Health Sciences<br>Richardson, Alison; University of Southampton, Faculty of Health Sciences<br>Myall, Michelle; University of Southampton, Faculty of Health Sciences
**Primary Subject Heading**:	Health services research
Secondary Subject Heading:	Communication, Health policy, Palliative care, Emergency medicine
Keywords:	Goals of Care, Ceilings of Care, Treatment Escalation Plans, Decision Making, Normalisation Process Theory, Implementation Science

For peer review only

Implementing communication and decision-making interventions directed at goals of care: a theory led scoping review

Amanda Cummings[1,2], Susi Lund[1,2], Natasha Campling[1,2], Carl May[1,2,3], Alison Richardson[1,2,3], Michelle Myall[1,2]

1. Faculty of Health Sciences, University of Southampton, UK
2. NIHR CLAHRC Wessex, University of Southampton, UK
3. University Hospital Southampton NHS Foundation Trust, Southampton, UK

Corresponding author: Dr Amanda Cummings, Faculty of Health Sciences, University of Southampton, Building 67, University Road, Highfield, Southampton, SO17 1BJ
Tel: +44 (0)23 8059 8250; Fax +44 (0)23 8059 8308; Email: ac2a13@soton.ac.uk

Key words: goals of care; ceilings of care; treatment escalation plans; decision-making; implementation science; Normalisation Process Theory; scoping review

Word count: 5,012 (excluding tables and references)

For peer review only

ABSTRACT

Objectives: To identify the factors that promote and inhibit the implementation of interventions which improve communication and decision-making directed at goals of care in the event of acute clinical deterioration.

Design and methods: A scoping review was undertaken based on the methodological framework of Arksey and O'Malley for conducting this type of review. Searches were carried out in MEDLINE and CINAHL to identify peer reviewed papers and in Google to identify grey literature. Searches were limited to those published in the English language from 2000 onwards. Inclusion and exclusion criteria were applied, and only papers that had a specific focus on implementation in practice were selected. Data extracted were treated as qualitative and subjected to directed content analysis. A theory informed coding framework using Normalisation Process Theory (NPT) was applied to characterise and explain implementation processes.

Results: Searches identified 2,619 citations, 43 of which met the inclusion criteria. Analysis generated six themes fundamental to successful implementation of goals of care interventions: (1) input into development; (2) key clinical proponents; (3) training and education; (4) intervention workability and functionality; (5) setting and context; and (6) perceived value and appraisal.

Conclusions: A broad and diverse literature focusing on implementation of goals of care interventions was identified. Our review recognised these interventions as both complex and contentious in nature making their incorporation into routine clinical practice dependent on a number of factors. Implementing such interventions present challenges at individual, organisational and systems levels, which make them difficult to introduce and embed. We have identified a series of factors that influence successful implementation and our analysis has distilled key learning points, conceptualised as a set of propositions, we consider relevant to implementing other complex and contentious interventions.

Strengths and limitations of this study

- This paper provides a scoping review of a broad and diverse literature focusing on the implementation of goals of care interventions in a range of healthcare settings for patients facing clinical deterioration.
- The review defines goals of care interventions as 'contentious' with a moral purpose and value, and identifies elements that comprise such interventions and learnings that could be generalizable beyond goals of care and applied to other complex and contentious interventions and processes.
- This review utilises Normalization Process Theory (NPT) to investigate and explain the successful implementation of interventions, and to our knowledge offers a different approach to other reviews, by applying a theoretical approach to all stages of the review process.
- The inclusion of published and grey literature allows diverse material relevant to the implementation of goals of care interventions in practice to be captured.
- This review focuses on the factors that promote or inhibit the implementation of goals of care interventions, as such, not all goals of care interventions are covered, which is a limitation of the study.

For peer review only - http://bmjopen.bmj.com/site/about/guidelines.xhtml

For peer review only

1. INTRODUCTION

In the event of a patient becoming acutely unwell, treatment and care decisions are recommended by clinicians. While these decisions are based on the clinical judgement of a healthcare professional they should also be bound by the preferences and wishes of the patient and their family. Processes and tools (referred to here as goals of care interventions), that provide a framework for discussing and documenting appropriate treatment options in the event of acute clinical deterioration, are paramount. These interventions aim to improve patient and family involvement, enabling exploration and understanding of the current clinical situation and facilitation of communication and negotiated decision-making about future treatment options [1, 2]. They offer a means for patients' preferences to be taken into account, improving communication and clarity across the wider clinical team.

We present a scoping review of goals of care interventions which aims to: *identify the factors that promote and inhibit the implementation of interventions which improve communication and decision-making directed at goals of care.* We have used Normalisation Process Theory (NPT) to characterise and explain implementation processes [3]. NPT provides a set of tools to investigate and understand the processes through which interventions are operationalised in healthcare settings and incorporated into everyday practice [3-6]. We have previously used NPT to aid learning in the comparable context of advance care plans [7].

We refer to goals of care in the event of acute deterioration, where different levels of treatment might be appropriate and range from full escalation in a critical care environment to symptom control measures [8]. Goals of care are currently referred to using various terms, including but not confined to ceilings of care, treatment escalation plans and treatment limitations. They exist in numerous formations including a specific, dedicated paper form, a narrative entry in a paper medical record and inclusion in an electronic patient record, and may be introduced as an extension of the 'do not attempt cardio-pulmonary resuscitation' (DNACPR) process. These interventions offer a system for recording recommended treatment and care, ideally including all components of the decision pathway and can be applied across different care settings. They require a process to be created and implemented that is recognised across organisations, takes account of socio-legal frameworks, such as the UK Mental Capacity Act (MCA) (2005) [9], and is designed to protect individuals who do not have capacity to make decisions about their care and treatment.

Goals of care interventions are complex and consist of multiple interacting components. The number and difficulty of behaviours required by those delivering or receiving such an intervention, variability of outcomes, and degree of flexibility and tailoring that is permitted, all contribute to making this a complex intervention [10]. As well as being complex, goals of care interventions are established as interactions and recording systems with a moral purpose and value. This means they may be *contentious* in practice because they contain elements which seek to routinise highly complex clinical skills, practice and different types of wisdom, in a context of uncertainty. Contentiousness can arise because the intervention relies on patient, family and clinician interactions, inter-clinician interactions (potentially across clinical settings and organisational boundaries), and societal and legal frameworks, such as the MCA (2005) and the European Convention on Human Rights (ECHR) (2002) [9, 11].

For peer review only - http://bmjopen.bmj.com/site/about/guidelines.xhtml

In spite of a growing body of literature describing the introduction and benefits of goals of care interventions, little is known about the factors which influence their successful implementation in clinical practice. Here implementation is defined as 'any deliberately initiated attempt to introduce new, or modify existing, patterns of action in health care or some other formal organizational setting. Deliberate initiation means that an intervention is: institutionally sanctioned; formally defined; consciously planned; and intended to lead to a changed outcome' [12]. As we have previously argued, "this is more than the adoption or diffusion of innovations" as effective implementation is about interventions being made workable and embedded in routine clinical practice [12].

Understanding and evaluating the implementation of complex interventions in practice remains a challenge for healthcare managers, policy makers and for those who enact them outside of formal research settings [13]. Furthermore, as interventions found to be effective in the context of health services research studies can fail to translate into meaningful healthcare outcomes across varying contexts [14] this makes understanding the reasons for failure or partial success even more essential. Implementation science, which promotes the integration of research findings and evidence into healthcare policy and practice, is increasingly recognised within health services research to make a key contribution to such knowledge. Comprehensive process evaluation of the implementation of healthcare interventions is increasingly important for future learning [15], as it enables understanding of transition from closed systems of highly structured research or service development projects into the real world of open systems healthcare delivery where they are operationalised [16, 17]. Learning from the available existing knowledge in this area can be used to inform healthcare practice change and contribute to the field of implementation science.

2. DESIGN AND METHODS

A scoping review was the most appropriate methodology, given the need to extract and map principles from a diverse and broad body of evidence [18, 19].

We used Arksey and O'Malley's five stage framework for conducting scoping reviews which includes: identifying the research question, identifying relevant literature, selection, charting the data and collating, summarising and reporting the results [20]. This guided the scoping review and where necessary we developed more specific procedures to inform the review process. Levac et al's recommendations for refining the methodological application were also incorporated to increase rigour of the review process [21].

Inclusion and exclusion criteria

Literature was selected using specific inclusion and exclusion criteria (Table 1), and was included if it was considered to have a specific focus on implementation in practice. To identify all relevant literature on goals of care, grey literature was included and actively sought as part of the search strategy. Only papers published from the year 2000 onwards were included as those published earlier were unlikely to reflect current practice.

For peer review only - http://bmjopen.bmj.com/site/about/guidelines.xhtml

Table 1. Inclusion and exclusion eligibility criteria

Inclusion criteria	Exclusion criteria
Literature describing implementation of interventions related to communication and decision-making around goals of care.<br>Studies with adult patients in hospital and community settings.<br>Studies involving end of life care, clinical deterioration and clinically uncertain outcomes.<br>Studies published in the English language.<br>Papers published between 2000-2015.<br>Qualitative and quantitative studies, including clinical trials and RCTs.<br>Published conference abstracts/conference related papers<br>Grey literature (limited to: policies, reports, research posters, patient/staff guidance, websites).	Papers not describing an intervention (process or tool).<br>Papers reporting treatment effectiveness.<br>Papers describing DNACPR orders only.<br>Studies in neonatal and paediatric settings.<br>Studies involving brain stem death.<br>Studies using biomedical data and drug trials.<br>Non-English language studies<br>Papers published before 1 January 2000.

Search strategy and information sources

The search strategy was designed to identify primary studies and other literature, both published and unpublished, which met the eligibility criteria. Separate searches were undertaken for primary and grey literature.

Primary literature searches

The primary literature search was carried out in two stages. The first stage involved an initial search of the bibliographic database MEDLINE using a preliminary key word search based on the terms of the topic, the text terms used in titles and abstracts, and index terms used to describe articles. Full details of the primary literature search strategy are outlined in appendix 1.

Terms identified in the titles and abstracts of relevant articles produced from stage 1 were used to develop further key words for the second stage of the literature search (see appendix 1). Dual combined key word searches were conducted in MEDLINE and CINAHL bibliographic databases, with a third key word added if searches produced >200 results. In addition, further literature were identified through existing knowledge and networks.

Grey literature searches

Google searches were conducted using terminology relating to known goals of care interventions identified in an earlier scoping exercise which mapped the use of forms for recording these decisions in the UK. The first 10 results were screened for relevance and further review. A further Google search focused on policies and guidance related to goals of care was undertaken and the first 50 results screened for relevance.

For peer review only - http://bmjopen.bmj.com/site/about/guidelines.xhtml

Searches were completed by August 2015. All screened, de-duplicated citations were imported into the bibliographic software management package Endnote.

Screening

An extensive screening process was undertaken. At the first stage of screening, articles were assessed by two independent reviewers (AC, CRM) based on the information provided in the title. Primary literature judged to be relevant after first screen, and which met the eligibility criteria, were obtained in full text. Articles for which there was disagreement between reviewers, were also obtained in full text. Full text articles were examined for adherence to the inclusion criteria and then screened (by AC, with input from CRM and MM, in cases of uncertainty or disagreement). Grey literature were title screened for relevance and a further full review examined adherence to inclusion criteria.

Quality assessment

This scoping review included a non-heterogeneous sample of primary and grey literature, which made it difficult to universally apply quality assessment criteria. As standard to most scoping reviews, we did not undertake formal quality assessment and excluded papers only on grounds of relevance. As a result the analytical focus of this review centred on a critique of relevance and contribution of the included literature and did not consider methodological quality.

Data extraction

In line with Arksey and O'Malley's method [20], data extraction (charting) was multi-staged. In the first stage we collected descriptive characteristics from each paper such as study design and setting. In the second stage, findings and discussion sections of included literature were extracted into a data extraction tool (see appendix 2) informed by NPT [4-6].

The data extraction tool was designed to chart specific details of the literature and to understand factors influencing implementation. The tool was piloted on a sample (n=5) of primary and grey literature, with subsequent amendments resulting in the final version. Data was extracted by independent reviewers (AC, SL, MB). Following Levac et al's recommendations, two reviewers (AC, MM) independently extracted data for a 30% sample of primary literature to ensure approaches to extraction were consistent with each other and with the research aims [21].

Data analysis

Data extracted were treated as qualitative data and analysed using directed content analysis [22]. A theory informed coding framework was developed using the four main constructs and sub-constructs of NPT [4, 5] was used. Analysis was undertaken to identify, characterise and explain the factors that shape implementation of interventions that initiate communication and decision-making around goals of care. Data were identified, categorised and thematically analysed to the constructs and sub-constructs of NPT, exploring barriers and facilitators to implementation.

The use of NPT as a theoretical framework followed its successful application in a number of different healthcare intervention reviews [7, 23-27]. NPT investigates and explains the successful operationalization of interventions: how they become part of everyday practice in healthcare settings. It embodies the different types of 'work' undertaken by individuals around implementing, embedding and integrating, and

For peer review only - http://bmjopen.bmj.com/site/about/guidelines.xhtml

allows us to understand the social structures and contexts through which new interventions are operationalised [3-6]. In relation to implementation of goals of care interventions, definitions of the four core constructs and sub-constructs of NPT used in this review are outlined in appendix 3.

3. RESULTS

Searches identified 2619 citations. Following de-duplication and relevance screening, 43 sources of literature (relating to 23 interventions), including 24 items of published literature (e.g. peer-reviewed papers, conference abstracts) and 19 items of grey literature (e.g. conference posters, patient information documents), met the inclusion criteria and were included for data extraction (see figure 1). Table 2 outlines details of the included literature, the range of interventions described and the decisions of interest addressed, including DNACPR, goals of active care, supportive or palliative care and those which are limited to communication guidelines only.

For peer review only

Insert Figure 1. Here

For peer review only - http://bmjopen.bmj.com/site/about/guidelines.xhtml

Table 2. Summary of included papers

Name and description of intervention	Decisions of interest				Author & Year	Country & Healthcare Setting	Literature Type	Study Design	Study Objective
	DNACPR	Ceilings of active care	Supportive/Palliative Care	Goals of care communication guidelines					
AMBER care bundle<br><br>Care bundle consisting of a proforma and sticker.<br>Promotes consistent communication and care planning for patients identified as facing uncertain recovery.	X	X			Carey et al 2014[28]	UK Hospital	*Published peer-reviewed study*	Descriptive: Development and implementation review	To describe the design, development and implementation of the AMBER care bundle.
					Etkind et al 2014[29]	UK Hospital	*Published peer-reviewed study*	Retrospective electronic case note review	To evaluate application of the AMBER care bundle in a UK hospital and describe factors affecting its use.
					Morris et al 2011[30]	UK Hospital	*Conference Abstract*	Descriptive	Learning points from implementation of the AMBER care bundle.
					Doncaster and Bassetlaw Hospitals NHS Foundation Trust 2015[31]	UK	*Grey Literature*	Policy Document	Management of patients who are at end of life.
					Guy's and St Thomas' NHS Foundation Trust 2012[32]	UK	*Grey Literature*	Patient Guidance	The AMBER care bundle – a guide for patients, their relatives and carers.
Universal Form Of Treatment Options (UFTO)<br><br>Form contextualising the CPR decision within overall treatment plans.	X	X	X		Fritz et al 2013[8]	UK Hospital	*Published peer-reviewed study*	Prospective mixed methods before and after study	To determine whether the introduction of the UFTO reduces harms in patients in whom a decision not to attempt CPR was made.
					Fritz et al	UK	*Published peer-*	Descriptive: Cross-	To describe a cross-disciplinary

For peer review only - http://bmjopen.bmj.com/site/about/guidelines.xhtml

Includes documentation and discussion of care and treatment decisions.					2015[33]	Hospital	*reviewed study*	disciplinary adapted Delphi method	approach to developing a universal form of treatment options as an alternative to DNACPR orders.
					www.ufto.org Accessed: 01/07/15[34]	UK	*Grey Literature*	Website	UFTO: Universal form of treatment options
					www.ufto.org/background/scientific-background Accessed 01/07/15[35]	UK	*Grey Literature*	Website	UFTO: Scientific background
Goals of Patient Care Summary (GOPC)<br><br>Form to summarise goals of care. Serves as a guide for treatment decisions as the patient's condition improves, stabilises, or deteriorates.	X	X	X		Brimblecombe et al 2014[36]	Australia Hospital	*Published peer-reviewed study*	Single-centre observational cross-sectional study	To describe the implementation of the GOPC, quantify its completion and review outcomes of the GOPC assessment.
Physician Orders for Life-Sustaining Treatment (POLST)<br><br>Form to ensure that patient wishes regarding life-sustaining treatments are honoured.<br>Converts treatment preferences into medical orders.	X	X	X		Hickman et al 2009[37]	USA Hospice	*Published peer-reviewed study*	Descriptive and evaluation	To evaluate use of the POLST by hospice programs.
Gold Standards Framework for Care Homes (GSFCH) and an adapted Liverpool Care Pathway for Care Homes (LCP)<br><br>The implementation of two end-of-life care tools: the GSFCH supportive/palliative care register and an adapted Liverpool Care Pathway for Care Homes.	X	X	X		Hockley et al 2010[38]	UK Nursing Home	*Published peer-reviewed study*	A multi-centre implementation trial and in-depth evaluation	To evaluate the effectiveness of a high facilitation implementation of two end of life care tools in the nursing home setting.
UIHC (University of Iowa Hospital and Clinics) Policy Regarding Do Not Resuscitate Orders and End of Life Discussions with Hospitalised	X	X	X		Kaldjian et al 2011[39]	USA Hospital	*Published peer-reviewed study*	Descriptive: the integration of goals of care into education and revision of hospital	To describe the background and rationale for DNR policy revision and development. To describe the policy itself and ongoing

For peer review only

For peer review only - http://bmjopen.bmj.com/site/about/guidelines.xhtml

Patients<br><br>DNR order policy placing CPR discussions within a framework of end of life discussions, and within a larger goal orientated context.								policy. Includes a structured literature review and a survey of hospitalised adults	implementation efforts.
A revised DNACPR form, decision making tools and updated CPR guidelines	X	X	X		Mbriwa et al 2014[40]	UK Hospital	*Published peer-reviewed study*	Prospective case note audit	To assess any improvement in CPR decision making, ceiling of care documentation and discussions since the introduction of a revised DNACPR form, decision making tools and guidelines.
Providers Signout for Scope of Treatment (PSOST)<br><br>A daily handover tool documenting resuscitation status, goals of care, desired and undesired interventions, in addition to standard handover information.	X	X	X		Newport et al 2010[41]	USA Palliative care in-patient unit	*Published peer-reviewed study*	Descriptive	To describe the PSOST tool and its implementation.
An intervention informing clinicians of the laws on Patients' Rights and the importance of advanced care planning, alongside a new DNR form<br><br>Adapted DNR form, putting emphasis on the motivation, participants involved and communication of the decision-making process.	X	X			Piers et al 2011[42]	Belgium Hospital	*Published peer-reviewed study*	A prospective observational study	To compare the quality of end-of-life policy between hospital wards after a hospital-wide intervention to improve advanced care planning.
Treatment Escalation Plan and Resuscitation Decision (TEP-CPR)<br><br>Treatment escalation plan tool, to be included in the resuscitation form.	X	X	X		Stockdale et al 2013[43]	UK Hospital	*Published peer-reviewed study*	Descriptive and evaluation: Notes audit, survey and development and testing of TEP using PDSA cycles	To design and test a tool for escalation planning. To identify whether a TEP could reduce the number of patients receiving inappropriate treatments out of hours.
Ceiling of Treatment (COT) Form<br><br>Tool for improving decision making and documentation of treatment	X	X	X		Dahill et al 2013[44]	UK Hospital	*Published peer-reviewed study*	A quality improvement project and pilot study	To improve decision-making and the documentation of treatment escalation decisions.
					Powter et al	UK	*Grey Literature*	Poster	To improve decision making and

For peer review only

escalation decisions					2012[45]	Hospital			documentation of CPR and ceiling of treatment decisions.
Treatment Escalation Plan (Devon)<br><br>Form outlining what treatment options would be appropriate if a patient were to become acutely unwell.	X	X			Obolensky et al 2010[1]	UK Hospital	*Published peer-reviewed study*	A prospective quantitative and qualitative evaluation	To evaluate patient and relative experiences/thoughts regarding the Devon TEP.
					Mercer, M 2009[46]	UK Hospital	*Published Letter*	Letter to the editor	The death of DNR: Treatment escalation plans.
					Obolensky et al 2007[47]	UK Hospital	*Conference Abstract*	Evaluation	To evaluate initial impact of the Devon TEP from a clinical staff perspective.
					Obolensky et al 2007[48]	UK Hospital	*Conference Abstract*	Evaluation	To evaluate patient and relative experiences of the Devon TEP.
					Karakusevic, C [49]	UK Hospital	*Grey Literature*	Poster	To evaluate use of the Devon TEP.
					Torbay and Southern Devon Health and Care NHS Trust 2014[50]	UK	*Grey Literature*	Policy Document	Treatment escalation plans (TEP) and resuscitation decision records policy.
					Care Quality Commission (CQC) 2014[51]	UK	*Grey Literature*	CQC Report	Northern Devon healthcare NHS trust, end of life care quality report.
					Rowcroft Hospice 2013[52]	UK	*Grey Literature*	Patient Guidance	Treatment escalation plan (TEP) forms, public information document.
					Devon Local Medical Committee 2013[53]	UK	*Grey Literature*	Staff Guidance	Treatment escalation plans (TEP) – update article.
					Macmillan Cancer Support, St Luke's Hospice Plymouth & North, East and West Devon CCG[54]	UK	*Grey Literature*	Staff Guidance	New TEP form (version 10) – Frequently asked questions document.
					North Devon Healthcare NHS Trust[55]	UK	*Grey Literature*	Staff Guidance	Staff briefing note on the use of treatment escalation plans (TEPs).

For peer review only

For peer review only - http://bmjopen.bmj.com/site/about/guidelines.xhtml

					NHS Improving Quality Team 2014[56]	UK	*Grey Literature*	Staff Guidance	Guidance for completing treatment escalation plan and resuscitation decisions.
Treatment Escalation Plan (TEP)<br><br>Tool for planning the care of a patient at risk of deterioration.	X	X	X		Paes, P et al 2012[57]	UK Hospital	*Conference Abstract*	Pilot, audit and evaluation	To pilot and audit the TEP, evaluating decision making before and after its introduction across select general hospital wards.
					Paes, P 2012[58]	UK Hospital	*Grey Literature*	Poster	To pilot and evaluate effectiveness of TEP across acute hospital wards, palliative care and a community hospital.
HDU Specific Treatment Escalation Plan<br><br>Plan for ceiling of treatment and escalation level decision on every patient within 24hrs.	X	X			Hannah, J 2014[59]	UK Hospital	*Grey Literature*	Poster	To evaluate the process and effects, of introducing a TEP in a medical high dependency unit.
Treatment Escalation Plan Proforma	X	X	X		Thomson et al 2014[60]	UK Hospital	*Grey Literature*	Poster	To evaluate outcomes from piloting a TEP proforma in acute medicine.
Cornwall Treatment Escalation Plan and Resuscitation Decision Record	*X*	*X*			Royal Cornwall Hospitals NHS Trust 2015[61]	UK	*Grey Literature*	Policy Document	Treatment escalation plan & resuscitation decision record policy.
Escalation plan and resuscitation status (EPARS)<br><br>Form amalgamating escalation plan and CPR decisions.	X	X	X		West Suffolk NHS Foundation Trust 2013[62]	UK	*Grey Literature*	Policy Document	Trust policy and procedure: Escalation plan and resuscitation status (EPARS) (including DNACPR).
Treatment Escalation Plan (TEP)	X	X			Basildon and Thurrock University Hospitals NHS Foundation Trust 2014[63]	UK	*Grey Literature*	Patient Guidance	Treatment escalation plan (TEP) patient information.
Content guidelines for discussions of CPR and life-sustaining therapy<br><br>Guidelines for the content of a discussion about resuscitation or goals of care.	X	X		X	Downar et al 2010[64]	Canada Hospital	*Published peer-reviewed study*	Descriptive: Delphi method of consensus generation to develop content guidelines.	To develop content guidelines for discussions of CPR and life-sustaining therapy.

For peer review only

For peer review only - http://bmjopen.bmj.com/site/about/guidelines.xhtml

A palliative care treatment plan intervention<br><br>Best practice guidance supported by a staff education programme, electronic decision support tool and paper educational tools.			X		Bailey et al 2014[65]	USA Hospital	*Published peer-reviewed study*	Multi-centre implementation trial.	To evaluate the effectiveness of a multi-modal intervention strategy to improve the processes and quality of end-of-life care in acute inpatient settings.
The Palliative Care for Advanced Disease (PCAD) pathway<br><br>To improve palliative care for inpatients who are expected to die from advanced disease. Clinical pathway including:<br>- Inter-disciplinary care pathway<br>- Nurses documentation flowsheet<br>- Physicians order sheet with guidelines for symptom management			X		Bookbinder et al 2005[66]	USA Hospital	*Published peer-reviewed study*	Evaluation study (of clinical pathway and implementation strategy)	To describe the development and quality improvement strategy for implementation of PCAD.
Serious illness conversation guide<br><br>Guide describing key elements to be addressed in patient conversations about serious illness care goals.			X	X	Bernacki et al 2014[67]	USA	*Published peer-reviewed study*	Literature review and synthesis of best practices	To provide clinicians with practical, evidence-based advice (in the form of a serious illness conversation guide).
The VOICE (Values and Options in Cancer Care) intervention<br><br>Education and coaching communication intervention.<br>To facilitate communication and decision making among oncologists, patients and caregivers.				X	Hoerger et al 2013[68]	USA Oncology Services	*Published peer-reviewed study*	Descriptive<br><br>(The intervention is being evaluated as part of an RCT which is also described in this paper)	To describe the intervention in relation to its aims of enhancing patient-centred communication, quality of care, and patient outcomes.

For peer review only - http://bmjopen.bmj.com/site/about/guidelines.xhtml

Results are presented in table 3 as a summary and appraisal of the literature. Thematic analysis of literature content suggested there were six common themes (see appendix 4) fundamental to the successful implementation of goals of care interventions: input into development, key clinical proponents, training and education, intervention workability and functionality, setting and context, perceived value and appraisal. These are outlined below. The links between these identified themes and the sub-constructs of NPT are outlined in table 3.

Input into development

The involvement of clinical staff in development of interventions facilitated the identification of current shortfalls in practice, understanding purpose, and shaping novelty, accessibility and utility of design [33, 38, 43, 44, 68]. Developmental input also assisted in promoting 'buy-in', legitimization and confidence in the intervention [28, 33, 38, 41-45, 59, 64]. While some literature focused on the importance of input from senior, specialist individuals [28, 38, 43, 64], including those within implementation settings [43, 44, 66], for intervention design, multi-disciplinary collaboration in the development process was common across papers [8, 28, 33, 35, 43-45, 49, 59, 62, 64, 66], and helpful for gaining collective knowledge from all potential users [28, 33, 44, 45, 59]. In one paper, individuals who had expressed disagreement with one intervention were actively sought for involvement at each stage of development [33], highlighting how inclusion in development can contribute to overcoming barriers of individual resistance. Reconfiguration of forms and procedures were incorporated in the development and implementation process in a number of initiatives [28, 33, 43, 44, 66]. The benefit of this approach was that it allowed for assessment of factors such as usability, clarity and safety, incorporating feedback into further iterations [33, 43, 66].

Key clinical proponents

Key clinical proponents were assigned to orchestrate and lead implementation within specific sites or settings [38, 51, 65, 66]. They were key resources for attending and cascading training as well as overseeing changes, embedding cultural change and providing mentorship [30, 38, 51, 65, 66]. Their direct contact with intervention instigators appeared important in orientating and maintaining momentum of local leadership, including training and facilitation [38, 65, 66]. Similarly, support from individual senior managers in ensuring compliance with training and interventions, promoting sustained use, as well as acting as catalysts for initial implementation was valuable [38, 51, 61, 65, 66, 68]. Trust level managerial support was also evident through incorporation into policy and protocol [31, 39, 44, 50, 61, 62] and approval from directors [45, 59]. Managerial backing could be influential for organisational change, although in reality, engagement with the management of healthcare organizations was recognised by some for its complexity [38, 66].

Training and education

Training and education encouraged 'buy-in' [8, 28, 33, 38, 39, 45, 50, 51, 65, 66], promoted understanding of tasks and responsibilities [28, 33, 39, 45, 65, 66] and facilitated a shared understanding of purpose [39, 49, 65]. Staff often negatively perceived the increased training requirement [33], inclusion within established working practices [39, 45, 65, 66] and feasibility of incorporation into workload [66, 68] were important considerations. Repeated sessions were used to train rotational/part-time staff, address staff turnover and enable sustained engagement [28, 44, 45, 66]. Tailored education based on evidence and

For peer review only - http://bmjopen.bmj.com/site/about/guidelines.xhtml

feedback facilitated the most efficacious training, especially since misunderstanding of responsibilities could result in ineffective or reduced use [33, 36, 38, 51, 66]. Training in the application of skills to the intervention was fundamental [61, 65, 66] and without this, regardless of skill level, the completion, interpretation and application of interventions in practice could be inconsistent [37, 49, 51]. Addressing communication skills was highlighted as important in a number of papers [28, 30, 36, 42, 68]. Individuals responsible for training could ensure that as far as possible all staff received necessary training [61, 65, 66]. The provision of guides, algorithms and interactive materials facilitated skill development [65, 68], and patient and carer information materials helped to sustain practice around newly implemented interventions [31-33, 39, 44, 48, 50, 52, 61-63, 65, 66].

Intervention workability and functionality

A frequently adopted approach included using existing programs or frameworks (local or otherwise) as the basis for new intervention development [37, 38, 40-43, 66]. Literature suggested healthcare practices can flex to incorporate interventions designed as part of, or alongside, existing processes [8, 28, 35, 36, 43, 61, 65]. This approach was often more acceptable to staff, promoting incorporation into practice, adherence and behavioural change [8, 35, 36, 43]. Conversely, lack of transferability across healthcare settings was viewed as a barrier to implementation [37, 41]. The workability of interventions was important, with the integral use of guidelines and prompts facilitating intervention utility and accessibility [1, 8, 28, 29, 33, 36, 37, 39, 41, 44-46, 57-62, 64-68]. The use of stickers and brightly coloured forms for insertion into patient medical records promoted easy recognition of interventions [28, 37]. For many, successful incorporation into working practices appeared to rely upon the paper format of tools, their design and usability [1, 33, 46, 57, 61, 62].

Setting and context

Implementation often occurred within limited clinical settings, and the direct relevance of the specialism (to the intervention) was highlighted as important for promoting its value [29, 41-43, 57, 58, 60, 66]. Subsequent diffusion from implementation setting to hospital-wide acceptance was demonstrated in relation to a number of interventions [1, 28, 33, 66]. Despite this, multiple barriers and enablers to change were identified including: transferability across primary and secondary care settings [37, 61], staff turnover and management stability [38], availability of staff for updates, counter signatories to meet completion timeframes [36, 37, 41], time for communication processes [43], robustness of implementation sites [38, 66], and the difficulty in transforming clinician attitudes [42, 59, 66].

The clinical complexity and unpredictability surrounding the patients for whom the interventions were targeted often affected successful or opportune application [29, 43]. Utility was improved where deterioration was predictable [28, 29, 43], yet recognition of deterioration was on occasion inherently difficult [29]. Clinical uncertainty meant that at times interventions were not fully utilised for the patients they were designed to support [28, 43].

Perceived value and appraisal

Staff perceptions and the degree of alignment with the intended purpose of interventions, were infrequently reported [33, 59, 66]. Staff valuing the intervention from the outset had important implications for effective application [33, 36, 39, 43] and perceived utility, existing supportive local policy

For peer review only - http://bmjopen.bmj.com/site/about/guidelines.xhtml

and inclusive collaborative development were all highlighted as facilitators [33, 35, 36, 38, 43, 59, 62, 64, 66, 68]. At an organisational level, responsiveness to national guidance and policy was likely to promote value [8, 38, 62]. Understanding objectives early on in the implementation pathway was key, as misunderstandings were not uncommon [28, 36, 49], and could lead to reduced or incomplete application of interventions [28, 36].

Following implementation, appraisal of intervention value was frequent [8, 37, 38, 43-45, 47, 57, 58, 60, 66], often demonstrating communal agreement centred around positive impact on working practices [8, 30, 37, 38, 41, 43-45, 47, 57-59, 66]. Increased workload was expressed as a concern by staff, mainly relating to the completion of tools and the need for patient or family discussions [1, 8, 41, 57, 58, 66]. On appraisal, interventions were perceived to be a worthwhile investment due to patient benefit and the improved clarity and time saved, as a result of decisions and discussions taking place earlier in the care trajectory [1, 8, 58]. A number of papers recommended or proposed outcome measures relating to patient and relative experience [8, 28, 37, 44, 45, 59, 66, 68], but only a small number reported utilising such measures [1, 30, 46, 48].

For peer review only

For peer review only

For peer review only - http://bmjopen.bmj.com/site/about/guidelines.xhtml

Table 3. Summary of findings by NPT construct

Core NPT Construct [5]	NPT Sub-Construct and Questions [5]	Key Findings	Identified Themes
Coherence	*Differentiation*<br>- Does the intervention differ, & if so, how is it different from current practices?<br>- What work has been undertaken to aid understanding of how the new intervention differs from current practice?	- High clarity & description of differentiation, & where relevant, explanation of aspects that were similar to or based upon existing practice: based upon existing national programmes of work [37, 41], frameworks [38] or guidelines [66]; established from the format of locally used tools [41], adapted from local trusts [43] or consisted of adaptations to pre-existing interventions [40, 42, 43]; entirely new interventions developed [1, 8, 28, 36, 47-49, 64], usually resulting from extensive scoping work [28, 33, 64].<br>- Interventions were developed to replace or complement existing practices or tools [1, 8, 30, 36, 38, 40, 43, 47-49].<br>- To understand how & why interventions should differ from current practices the following were drawn upon: case note reviews [43]; staff interviews [33]; scoping of published literature & guidelines [39, 64, 66]; review of local practices & contexts [28, 33, 44, 45, 58, 65].	Workability/functionality<br>Input into development
	Communal specification<br>- Do staff have a shared understanding of the purpose of the intervention?<br>- How has a shared understanding of the purpose of the intervention been built among staff within the organisation?	- Number of common themes highlighted as the key purposes of interventions: reducing unnecessary or inappropriate treatment [36, 41, 62]; enabling consideration of goals of care [28, 36, 39], guiding treatment planning & decision making [28, 36, 64, 68]; improving communication & discussions between patients & clinicians [28, 36, 37, 62, 68]; respecting patient wishes & ensuring appropriate care [28, 37, 41, 50]; improving the management of uncertainty & advanced planning [28, 62]; guiding smooth transitions of care out-of-hours & between different clinical settings [41, 43, 50, 59, 61]; improving documentation, accessibility & awareness of treatment plans [41, 43, 50, 59, 62]; providing uniformity & continuity of care [41, 50, 59, 61, 62].<br>- Background evidence & scoping work has the secondary purpose of informing staff as to the rationale & purpose of interventions [8, 28, 35, 39, 40, 43-45, 50, 64, 67].<br>- Multi-disciplinary collaboration in both development [8, 33, 43-45, 49] & education provision [39, 49, 65].<br>- Accessing alternative education programmes can act as a barrier to understanding purpose [66].<br>- Staff perception of purpose only minimally described [33, 59, 66], & some evidence of staff misunderstanding purpose [28, 36, 49].	Perceived value & appraisal<br>Input into development<br>Training & education
	Individual Specification<br>- Do staff understand their specific tasks & responsibilities in relation to the intervention? What work has been done to ensure they are understood?<br>- Do individuals understand how the intervention will impact upon the nature of their work?	- Importance of training [33, 45, 49, 65] & tailored education [33, 36] in facilitating staff understanding of tasks & responsibilities.<br>- Lack of training resulting in differing interpretations of an individuals' responsibilities for the same intervention [49].<br>- Lack of understanding of responsibilities or discomfort with proposed tasks leading to ineffective or reduced use [36-38, 51, 66].<br>- Methods of training delivery: incorporation into induction programmes [45]; simulated clinical scenarios [33]; presentations & meetings [65]; provision of material resources [33, 65].	Training & education<br>Perceived value & appraisal
	Internalization<br>- Do staff understand the potential value of the intervention for their work?<br>- What work has been done to promote understanding of the value, benefits & importance of the new intervention among staff & the organisation as a whole?	- Importance of implementation staff understanding the value of an intervention [33, 36, 39, 43].<br>- Foreseen advantages included prompting consideration of more forms of treatment & reducing the negative connotations of DNACPR [33, 43].<br>- Foreseen disadvantages included potential for staff confusion, committing patients to overtreatment & potential patient distress [33].<br>- Implementation area appears important in promoting value, & more challenging in specialities less used to practicing the concepts involved in the intervention [42, 66].<br>- Staff surveys [33, 43, 59, 68] & collaborative development [33, 35, 62, 64, 66] generated consensus as to the intervention's importance, & promoted value, often based on problems encountered in current practice [43, 68].<br>- National guidance & policy were drawn upon to highlight current inadequacies in practice interventions were designed to address [8, 38, 62]. Established local policy influenced the value attributed to an intervention [38].	Perceived value & appraisal<br>Setting & context<br>Input into development
Cognitive Participation	*Initiation*<br>- Are there key individuals identified as those driving the intervention forward?	- Majority of interventions piloted or implemented within limited settings or specific services [29, 41, 43, 57, 58, 60], often with input into the design from staff within these settings [41, 43, 60].<br>- Key clinical proponents [38, 51, 66] or multidisciplinary teams [65] were used to liaise & orchestrate implementation over multiple sites.<br>- Interventionist/facilitator contact important in orientating & maintaining momentum of local leadership [38, 65, 66].<br>- Input into design & development from: specialists within relevant specialties [28, 38, 43, 64] & multidisciplinary contributors, gaining collective knowledge from all potential users [28, 33, 44, 45, 59].	Key clinical proponents<br>Input into development<br>Setting & context

For peer review only - http://bmjopen.bmj.com/site/about/guidelines.xhtml

		- Senior clinicians or management responsible for compliance with new interventions [51, 61, 66].	
	Enrolment<br>- What work has been done to promote others to 'buy-in' & engage with the intervention, including training & education?<br>- Are people open to working with others in new ways to adopt the intervention?	- Training & education encouraged 'buy-in' [8, 28, 33, 38, 39, 45, 50, 51, 65, 66]: incorporated into established working practices [39, 45, 65, 66]; led by senior clinicians from relevant specialties [28, 38, 66]; consisted of multiple modalities [28, 38, 39, 65, 66].<br>- Timeliness of training delivery: prior to implementation [8, 33]; alongside implementation [38, 39, 50, 51, 65, 66]; repeated training [28, 66].<br>- Participation in development shown to promote 'buy-in' [33, 38, 42, 43, 45, 64]. Different techniques were adopted: anonymity of input [42, 64]; individual input in isolation, or prior to group consultation [33, 42, 43, 64]; actively involving those opposed to the intervention [33]; seeking opinions from staff experienced in the subject area targeted by the intervention [28, 33, 43, 64]; input into the intervention design [43, 44, 66] & implementation process [38, 43] from staff/specialists working within implementation settings.<br>- Difficulty in transforming clinician attitudes can act as a barrier to engagement [42].	Workability/functionality<br>Key clinical proponents<br>Training & education<br>Perceived value & appraisal<br>Input into development<br>Setting & context
	Legitimation<br>- Do staff believe that participating in the intervention is a legitimate part of their role?<br>- What work has been undertaken to ensure people believe it is right for them to be involved with the intervention?	- Legitimation was important, but its extent among clinicians varied [40, 58, 66].<br>- Facilitators of legitimation: prior use of the intervention [38]; perceived usefulness of the intervention [57, 58]; opportunities to provide input or suggest modifications to the intervention, irrespective of seniority [33, 43, 44, 64].<br>- Legality appeared as both a barrier & facilitator to legitimation [42, 43]: participation related to a sense of legal duty as opposed to behaviour change [42] & concern for potential legal ramifications were a barrier for clinicians [43].	Perceived value & appraisal<br>Input into development<br>Setting & context
	Activation<br>- Do people continue to support the intervention?<br>- What work has been undertaken to sustain the new intervention in practice & keep people supporting its use?	- Evidence of diffusion of uptake & cultural change across settings [1, 28, 33, 65, 66].<br>- Follow-up resources (including guidelines & policy), & mentorship facilitated implementation & continued support of interventions [31, 39, 44, 50, 61, 62, 65, 66].<br>- Consideration given to specialty & robustness of implementation site [38, 66].<br>- Some evidence of poor completion or failure to honour interventions [37, 49].	Setting & context<br>Key clinical proponents<br>Training & education<br>Perceived value & appraisal
Collective action	*Interactional Workability*<br>- Can the intervention be easily integrated into existing work practices?<br>- What work has been undertaken to allow the new intervention to integrate with existing staff & organisational practices?	- Ability to integrate with existing working practices recognised as important [28, 33, 65].<br>- Facilitators to integration: integration as part of, or in conjunction with, existing practices [8, 28, 35, 36, 43, 61, 65]; usefulness, pragmatism & accessibility of intervention [1, 8, 33, 41, 44-46, 57, 61, 62, 68]; involvement of users in design & piloting, allowing for amendments to facilitate utility [33, 38, 43, 44]; format of intervention based on existing design [41, 43]; transferability across healthcare settings [37, 41, 61].<br>- Barriers to integration: logistical & staffing barriers including staff turnover, management changes & staff availability to complete interventions [36-38, 41]; replication of existing work [44, 66]; clinical complexity [29, 43].<br>- Interactional workability of training was an important consideration [66, 68].<br>- Electronic medical health records (EMR): for some, configuration of interventions within EMR facilitated implementation [39, 65], whilst others described poor navigation of interventions within EMR [33, 49].	Workability/functionality<br>Input into development<br>Setting & context<br>Training & education
	Relational Integration<br>- Do individuals feel confident in their own & others' abilities to use the intervention?<br>- What work has been undertaken to build knowledge & confidence in using the new intervention?<br>- Does the intervention impact upon working relationships & what work has been done to minimise this?	- Circulation of interventions during development/early implementation appeared important for building confidence in their use [28, 33, 41, 49, 59] & allowed for amendments to facilitate understanding [49].<br>- Low confidence in particular aspects of interventions: capacity decisions & determining goals of care [36, 49].<br>- Reduced confidence in others: the ability of clinicians from other specialties to make decisions [33]; potential prematurity of decisions [33]; evidence of interventions not being followed due to disagreement by on-call clinicians [43].<br>- Increased confidence in others: clinicians keen to involve other clinicians & nurses expressed their confidence in others [33].<br>- Evidence of better teamwork & communication especially out-of-hours, driven by improved clarity & consistency of decisions [8, 28, 35, 41].<br>- Multi-modal format of interventions consisting of tools, prompts, algorithms & guidelines, invoke confidence in their application [1, 8, 28, 29, 33, 36, 37, 41, 45, 58-60, 64-67].<br>- Multidisciplinary educational programmes help build knowledge & confidence across disciplines [65, 66].	Workability/functionality<br>Input into development<br>Perceived value & appraisal<br>Setting & context<br>Training & education

For peer review only - http://bmjopen.bmj.com/site/about/guidelines.xhtml

	Skill set Workability<br>- Has the work (associated with the intervention) been assigned to those with the most appropriate skills?<br>- What formed the decision behind how the division of labour was allocated?<br>- Has sufficient training given staff the skills to enable them to use the intervention?	- Division of work based on level & nature of responsibility, among nurses, senior & junior clinicians [1, 8, 28-30, 33, 36-43, 64-68].<br>- Delegation often controlled for as the minimum level of clinicians permitted to complete or authorise was specified [1, 8, 33, 39, 43, 45, 59-62, 66].<br>- Colleagues should delegate if they do not have adequate skills [1], but concern was apparent if staff delegated to lacked the necessary experience/skills [39].<br>- Training & education was utilised [28, 36, 38, 65, 66], proposed [67, 68], or identified as deficient [37, 42], in facilitating staff development of skills, & application of skills to the processes involved in using interventions. The following were identified as important: skills training [36, 37, 39, 42, 49, 65, 66, 68]; communication skills (highlighted as difficult, important to clinicians & requiring development) [28, 30, 36, 42, 68]; other skills including: assessment & shared understanding of goals of care [1, 28, 30, 36, 42, 68]; training in application of skills to intervention [37, 49, 51, 61, 65, 66]; having someone in a position of responsibility for training execution/attendance [61, 65, 66]; different educational materials used alongside training programmes [65, 68]; value of not over-assuming existing skillset [49].<br>- Different types of training to teach relevant skills [28, 30, 65, 66]: teaching rounds [28, 65, 66] & formal group sessions [28, 66].<br>- Consideration of experience versus skill, & the interplay of external patient & organisational factors [39, 41].	Setting & context<br>Workability/functionality<br>Training & education<br>Key clinical proponents
	Contextual Integration<br>- What resources have been provided to support the new intervention in practice?<br>- Is the intervention adequately supported at managerial level?	- Patient & carer information leaflets are important [32, 33, 39, 48, 52, 63] & encourage shared communication [35, 61].<br>- A variety of staff guidance resources & updates used: newsletter updates, frequently asked questions documents, briefing notes, guidebooks, pocket cards, checklists, guidelines, frameworks, DVDs, prompt lists, algorithms, procedural leaflets [33, 39, 53-56, 61, 62, 65-67].<br>- A number of interventions also functioned as guidelines, with the use of flowcharts, prompts & checklists [1, 8, 28, 29, 33, 36, 37, 39, 41, 45, 58-60, 64-67], described as encouraging full & accurate completion [28, 39, 67].<br>- Joint patient & staff resources (poster & brochure) [33, 39].<br>- Guidelines incorporated in trust policy documents [33, 39, 61, 62, 65-67].<br>- Evidence from training scenarios used to shape information guidance, in order to address misconceptions evident during training [33].<br>- Guidance was distributed: alongside training sessions [68]; in staff inductions or updates [53, 61]; in local policy documents [62]; during or alongside implementation [33, 54-56, 65, 66]; as part of the intervention itself [66, 67].<br>- Websites: UFTO intervention has its own website providing access to resources, background to development, guidance for use & updates [34, 35]. One study refers to a palliative care website as a resource for implementation [66].<br>- Policy & protocol: three intervention specific policy documents [50, 61, 62]; one end of life care policy document referring to the intervention [31]; most relate to resuscitation & escalation planning [39, 44, 50, 61, 62], but also include trust induction policies [61] & EOLC policies [31].<br>- Evidence of managerial acceptance: approval from heads of department & medical directors [45, 59]; nationwide implementation [28]; incorporation into policy & protocol [31, 39, 44, 50, 61, 62].<br>- Managerial support acts as a catalyst to instigating & maintaining implementation [38, 65, 68].<br>- Levels of contact with management, & influence of management culture recognised [38, 66].	Training & education<br>Workability/functionality<br>Perceived value & appraisal<br>Key clinical proponents
Reflexive Monitoring	*Systemization*<br>- Has information been collected or are there plans to collect information to determine the usefulness of the intervention through feedback, audit or other means?<br>- Have the effects of the intervention been reported back to those involved?	- All describe or propose the collection of information relating to measures of usefulness [1, 8, 28-30, 35-40, 42-49, 51, 57-60, 65, 66].<br>- Data collection is proposed in studies where interventions have not yet been implemented [33, 41, 64, 67, 68].<br>- Different data collection methods: medical record audit [29, 36-38, 40, 43, 57, 58, 60, 65, 66], in the majority executed pre & post implementation, or in comparison with control groups [38, 40, 57, 58, 60, 65, 66]; staff surveys or interviews, often used in combination with medical record audit [8, 37, 38, 43-45, 47, 57, 58, 60, 66]; direct ward based observation [8]; patient interviews [1, 48].<br>- Different outcomes assessed: clinical impact of interventions (e.g. patient deaths) [8, 28, 29, 58]; clinical factors (e.g. illness trajectory) related to the use of interventions [29]; changes in end of life care processes, behaviours & outcomes (e.g. opioid prescriptions) [42, 43, 65, 66]; rates of completion, adherence & compliance with new interventions [28, 36-38, 40, 42, 43, 58, 66], & specifically with regard to documentation [36, 44, 45, 60]; new staff knowledge of care principles associated with the intervention [66]; attitudes toward the intervention [37, 60]; perceived effects of the intervention on practice [38, 47]; practicalities of the intervention [57, 58].<br>- Specific measures: study specific set of evaluation tools to measure feasibility [66] & validated measure to assess harms [8].<br>- Patient & family feedback outcomes are limited in scope & number [1, 8, 28, 30, 37, 44-46, 48, 59, 66, 68]: many recommend or propose the measure of patient & family experience [8, 28, 37, 44, 45, 59, 66, 68], some include measures of patient & family experience [1, 30,	Perceived value & appraisal<br>Training & education

For peer review only - http://bmjopen.bmj.com/site/about/guidelines.xhtml

For peer review only

		46, 48]. Although limited, feedback was positive & demonstrated preferences for new interventions over previous processes.<br>- Reporting the effects of the intervention to those involved: research publication [1, 8, 28-30, 35-40, 42-44, 46-48, 51, 57, 65, 66]; shared on intervention website [8, 35]; results communicated to promote shared learning [51]; shared with consultant panels for improved insight [44].	
	Communal appraisal<br>- Is there communal agreement among staff as to the value of the intervention?<br>- Has the worth of the intervention been evaluated collaboratively in formal or informal groups?	- One study evaluated staff perceptions in a collaborative setting, including focus groups & leadership meetings [66].<br>- Perceived value was evaluated via interviews, audits & questionnaires [8, 37, 38, 43-45, 47, 57, 58, 60, 66].<br>- The importance of individual & group methods in gauging attitudes was described, yet their relative efficiency was not explored [37, 38, 66].<br>- Communal staff appraisal may allow for a greater demonstration of impact than medical record audit data [66].<br>- Many demonstrated communal agreement as to the high level of value that staff attributed to an intervention [8, 37, 38, 44, 45, 47, 57-59, 66].<br>- Assessment of worth related to the intended (& perceived) purpose of interventions [28, 33, 36, 37, 39, 41, 43, 50, 59, 61, 62, 64, 66, 68].<br>- Only one study described negative staff perceptions [37].	Perceived value & appraisal
	Individual appraisal<br>- What is the effect of the intervention on an individuals' workload?<br>- Do individuals value the effect it has on their individual work?	- High value attributed to the intervention by staff, due to positive impact on working practices: reduces negative associations with DNACPR due to its universal application [8] or focus on positive treatment actions [47]; prevents unwanted resuscitation or escalation of care & ensures appropriate level of care [37, 41, 43, 44, 47]; provides a mechanism for forward planning: assessing, communicating & honouring patient preferences [8, 37]; increases staff confidence with, & improves clarity of, patient care plans [37, 41, 44, 47, 59]; improves team working & communication, especially out-of-hours [30, 43, 59]; improves ease & motivation of decision-making processes [41, 44, 57-59].<br>- Perceived & noted increases in workload were a concern for staff. However, it was generally perceived to be a worthwhile investment due to clarity, patient benefit & time saved later on [1, 8, 41, 57, 58, 66].	Workability/functionality<br>Perceived value & appraisal<br>Setting & context
	Reconfiguration<br>- Has appraisal work lead to attempts to improve or modify the intervention?	- Importance of appraisal & reconfiguration emphasised by evidence of its occurrence [28, 29, 33, 36, 38, 39, 43, 44, 48, 58, 59, 66].<br>- Reconfiguration part of the implementation process: adoption of PDSA (plan, do, study, act) cycles [43, 44]; piloting the intervention prior to roll out (with feedback, evaluation & subsequent amendments) [33]; early testing & adaptations [28]; use of sequential role out to amend implementation processes based on gathered insight [66].<br>- Appraisal & feedback led to suggested [66] & actual modifications [28, 33, 36, 43, 44, 58]: refinement of identification or trigger questions [28, 66]; ease of use, layout & clarity [33, 44]; inclusion & clarity of review dates [33, 43]; inclusion of free text boxes [33, 43].<br>- Appraisal led to further study or proposed new areas of study [29, 36, 58, 59, 66]: additional research to determine impact on specific outcomes [36]; feasibility of interventions over longer timeframes [66] & across different settings [29, 58]; studies to address distinct but associated problems identified during the appraisal work (e.g. lack of clinician presence at family meetings) [29, 59, 66].<br>- Appraisal also led to recognition of the need for, & subsequent development of, new patient information materials & services [48, 66], proposals for staff support services [38] & the revision & inclusion of interventions in local & trust policy [39, 44, 58].<br>- In two studies, appraisal demonstrated no desired impact, leading to the proposal or development of alternative interventions [40, 42].	Perceived value & appraisal<br>Input into development<br>Workability/functionality<br>Setting & context<br>Training & education

For peer review only - http://bmjopen.bmj.com/site/about/guidelines.xhtml

4. DISCUSSION

In this review we have identified a broad and diverse literature focusing on the implementation of goals of care interventions. Findings from this review suggest these interventions are both complex and contentious in nature, and as such it is conceivable that what we have learnt here applies to other contentious interventions and processes, for example discharge planning.

Our analysis has led us to characterise the elements that constitute contentious interventions. Using the example of goals of care interventions, we propose that these interventions consist of three components that intersect at three different levels in a system of negotiated interactions:

1. Negotiated decision making between clinicians, patients and family members, which is localised and characterised by its meaning for the individual.
2. The organisational procedure and collective system of making negotiated decisions.
3. The socio-legal constraints of taking account of preferences (including consent and capacity) that define the parameters.

Review findings point to negotiated interaction processes taking place at between individuals. This mediates a set of procedures about how decisions should be made: a set of expectations about what procedures should be done, what negotiations are possible, and what these look like within and across organisations. Socio-legal conceptualisation involves how organisations must deal with these preferences. All of these continuous components interact and affect a patient's care trajectory.

From the six themes generated from our analysis, we have extrapolated key learnings points for implementation, and conceptualised them as a series of propositions relevant to goals of care, and which we contend may apply across contentious interventions as a whole. These six propositions are:

1. *Individuals resist interventions that replicate the work of existing practices.*

The value of incorporating interventions into working practices has previously been described [13] and lies in minimising disruption. The adoption of existing intervention formats and integral guidelines was popular, and may act to improve successful compliance and integration through increased staff familiarity and confidence. However there is a fine line, as individuals resist interventions that replicate the work of existing practices [44, 66]. Interventions have to be easily differentiated from other practices to be valued. There needs to be clarity regarding the practices that are discontinued, preventing unnecessary duplication, and clear identification of the benefits of the new intervention. Evidence from this review suggests that high visibility methods (for example a sticker or coloured background) are valued for quick identification and time saving in clinical practice. These are features unique to the paper based nature of tools. Technological infrastructure facilitating electronic access to one record in all contexts will enhance the likelihood of widespread embedding across organisations.

For peer review only - http://bmjopen.bmj.com/site/about/guidelines.xhtml

2. *Contentious interventions are difficult to integrate in environments where there is clinical unpredictability and uncertainty.*

Interventions aimed at improving care for patients facing uncertainty can be difficult to integrate due to the very nature of complexity that exists for these patients and their clinicians. Usually the application of skills and techniques introduce order into such situations, with clinicians seeking scripts to work to. However, clinical uncertainty can render these scripts ineffectual, leading to uncertain outcomes. In light of this, studies in this review suggest the importance of the intervention being delivered by a clinician who has an established relationship with the patient and/or knows their situation well [39, 41]. While this represents the ideal, in the reality of clinical practice and a highly pressurised health service, this may prove difficult to achieve.

3. *Legitimacy is established where individuals build a shared understanding of purpose that enables them to attribute value to the intervention.*

The use of senior clinicians and managers in the development process is recognised for building a shared vision [69]. However, the potential for authoritative dominance of certain clinicians is recognised [70], and efforts must be made to enable active contributions from key members of junior staff. A high degree of clinical ownership is recognised as important for successful implementation [71]. This is more likely to be achieved if all clinicians, irrespective of seniority, believe they have a role to play and are making a valid contribution [72]. Key clinical proponents are essential in building legitimacy, successful and sustained implementation, especially in the context of organisational instability [73, 74].

4. *Training and education provide the framework for individuals to understand the value of what they do.*

Training and education has an important role in facilitating a shared understanding of purpose, addressing both moral and technical aspects, which are important for building value in the intervention. Training and education serves to facilitate this, fostering clarity of value in the intervention.

The importance of training and education is not a new concept. However, there is a lack of agreement regarding optimal delivery methods, which may relate to a need for training to fit within the clinical context. Findings from the review point to the need for multi-modal educational programmes, repetition and timeliness of training, but this should be considered in the context of staff and financial resource implications [75, 76].

5. *Appraisal work is critically important because it is how individuals value the intervention.*

The importance of sharing appraisal outcomes, conveying purpose and value to others is evident, as value is often not realised until successful implementation is achieved. However, reporting of this in the literature was limited [44, 51]. In particular, evaluation of patient and family perspectives was notably absent in the papers included in this review. We suggest their inclusion is crucial for imputing value into interventions which seek to include them [77]. Surveys suggest that clinicians will foresee advantages and disadvantages in any proposed intervention [33, 43]. Therefore, sharing appraisal work, which addresses the positives and negatives, may be beneficial in providing a more balanced view of an intervention's value.

For peer review only - http://bmjopen.bmj.com/site/about/guidelines.xhtml

For peer review only

6. The transfer of contentious interventions to other settings is problematic.

The initial setting can influence implementation success and subsequent transfer to other locations. In our review there is limited evidence of transfer at scale, over multiple geographical sites [65]. There are multiple barriers through which interventions need to filter, including specialisms, care locations, and structural and cultural factors. In addition, groups of clinicians hold different values, skills and knowledge. This introduces problems of discretion, interpretation and enactment within different contexts and may limit scale up and transferability. While not widely addressed by the papers in this review, wider implementation literature suggests the importance of involving patients and caregivers when transferring and implementing interventions beyond formal clinical settings [78, 79].

In summary, improving decision-making around goals of care and ensuring patient preferences are taken into account requires a better understanding of the implementation processes and factors that promote or impede implementation. We have used the exemplar of goals of care interventions to elucidate propositions in relation to the successful implementation of contentious interventions, where success refers to the routine incorporation and embedding of intervention components into everyday practice. We believe these propositions to be transferable and generalizable beyond the remit of goals of care, to other contentious (and complex) healthcare interventions.

Strengths and limitations

This paper presents a theory-led review of the barriers and facilitators to implementing a contentious and complex healthcare intervention. To our knowledge it offers a different approach to other reviews informed by a theoretical approach. The application of NPT to all stages of the review process, including data extraction and analysis, provided a useful framework for identifying, characterising and explaining the factors that promote or impede implementation of interventions for documenting decisions about patient treatment and care. It is also the first review with a focus on implementing goals of care to have included both published and grey literature, ensuring inclusion of material that addresses the incorporation of tools and processes in practice. Knowledge gained from this review is likely to be generalizable beyond goals of care interventions which may be of benefit to clinical practice and contribute to the field of implementation science.

However, as with any review ours has important limitations. In a number of the papers that contributed to this review information relating to barriers to implementation was missing and there was bias towards the presentation of positive outcomes. This may reflect a reluctance to focus on challenges encountered during the implementation phase with study authors keen to exhibit and promote the benefits of interventions. Reporting of barriers to implementation is beneficial for shared learning and future work. Therefore, future research should hold a greater focus on implementation, and on identifying the barriers to implementation for contentious interventions. Interventions which are tailored to barriers show greater capability and potential [80].

We are aware that some goals of care interventions, such as Deciding Right [81], were not included in the review. While citations relating to these interventions were identified in the search results, they were excluded during the screening process as implementation was not a focus of the paper.

For peer review only - http://bmjopen.bmj.com/site/about/guidelines.xhtml

We have proposed that contentious interventions consist of components at three levels. However, across the body of literature contained in this review the focus is on components at a level where the negotiated decision making between participants involved with completing the intervention occurs. There is limited focus on the components which take place within and across organisations and the constraints of the system into which the intervention is being implemented. This has important limitations for our analysis and interpretation of the data.

5. CONCLUSION

Findings from the review show that whilst such interventions are variable in design and use, there are a series of collective factors that influence successful implementation into routine clinical practice. We recommend that those seeking to introduce goals of care and other contentious interventions, consider the different facets of NPT, and use knowledge of this to develop implementation strategies. The contentious nature of these interventions means that their incorporation into everyday practice is dependent on a number of factors. Building a shared understanding of purpose that enables participants to attribute value to the intervention which is key, and both training and education and appraisal work play an important role in this process. Identifying clinical proponents who are able to, not only drive, but positively influence implementation is essential. Implementing complex and contentious interventions presents challenges which operate at an individual, organisational and systems level. It is these interaction level processes which make an intervention contentious, as well as making it challenging to introduce and embed. Success is more likely to occur, be established and sustained, if due attention is paid to the processes that facilitate operationalisation in the healthcare settings in which implementation occurs.

ACKNOWLEDGEMENTS

This review was funded by the National Institute for Health Research (NIHR) Collaboration for Leadership in Applied Health Research and Care Wessex. The views expressed are those of the authors and not necessarily those of the NHS, the NIHR or Department of Health. The authors would like to thank Dr Mark Banting for his assistance with data extraction in the initial phase of the review.

AUTHOR CONTRIBUTIONS

MM, CM and AR designed the review. AC performed searches. AC, and CM screened titles and abstracts and full paper screening was carried out by AC, MM and CM. Data extraction was performed by AC, MM and SL. AC and MM drafted the manuscript with assistance from SL, CM, NC and AR. All authors critically reviewed the manuscript for intellectual content and approved the final version of the paper. AC is guarantor.

FUNDING

This work was supported by the National Institute for Health Research Collaboration for Leadership in Applied Health Research and Care (NIHR CLAHRC) Wessex which is a partnership between Wessex NHS organisations and partners and the University of Southampton. Funders had no role in study design, data collection and analysis, decision to publish or preparation of the manuscript.

For peer review only - http://bmjopen.bmj.com/site/about/guidelines.xhtml

DISCLAIMER

The views expressed are those of the author(s) and not necessarily those of the NHS, the NIHR or the Department of Health.

COMPETING INTERESTS

The authors declare no competing interests.

DATA SHARING STATEMENT

No additional data are available.

REFERENCES

1. Obolensky L, Clark T, Matthew G, Mercer M. A patient and relative centred evaluation of treatment escalation plans: a replacement for the do-not-resuscitate process. *J Med Ethics* 2010;36(9):518-520.
2. Findlay GP, Shotton H, Kelly K, *et al*. *Time to intervene? A review of patients who underwent cardiopulmonary resuscitation as a result of an in-hospital cardiorespiratory arrest.* A report by the National Confidential Enquiry into Patient Outcome and Death, 2012.
3. May C, Finch T. Implementing, embedding, and integrating practices: an outline of normalization process theory. *Sociology* 2009;43(3):535-554.
4. May CR, Mair F, Finch T, *et al*. Development of a theory of implementation and integration: Normalization Process Theory. *Implementation Science* 2009;4(1):1.
5. May C, Rapley T, Mair FS, *et al*. *Normalization Process Theory On-line Users' Manual, Toolkit and NoMAD instrument*. 2015. Available at: http://www.normalizationprocess.org.
6. Finch TL, Girling M, May CR, *et al*. *NoMAD: Implementation measure based on Normalization Process Theory*. [Measurement instrument]. 2015. Retrieved from http://www.normalizationprocess.org.
7. Lund S, Richardson A, May C. Barriers to advance care planning at the end of life: an explanatory systematic review of implementation studies. *PLoS One* 2015;10(2):e0116629.
8. Fritz Z, Malyon A, Frankau JM, *et al*. The Universal Form of Treatment Options (UFTO) as an alternative to Do Not Attempt Cardiopulmonary Resuscitation (DNACPR) orders: a mixed methods evaluation of the effects on clinical practice and patient care. *PloS one* 2013;8(9):e70977.
9. Nicholson TR, Cutter W, Hotopf M. Assessing mental capacity: The mental capacity act. *BMJ* 2008;336(7639):322-325.
10. Craig P, Dieppe P, Macintyre S, *et al*. Developing and evaluating complex interventions: the new Medical Research Council guidance. *BMJ* 2008;337:a1655.

For peer review only - http://bmjopen.bmj.com/site/about/guidelines.xhtml

11. Council of Europe (1950) *European Convention for the Protection of Human Rights and Fundamental Freedoms, as amended by Protocols Nos. 11 and 14,* November 4 (ets, no. 5). Available at: http://www.refworld.org/docid/3ae6b3b04.html (Accessed: 08 March 2017).
12. May C, Finch T, Mair F, et al. Understanding the implementation of complex interventions in health care: the normalization process model. *BMC health services research* 2007;7(1):148.
13. May C. Agency and implementation: understanding the embedding of healthcare innovations in practice. *Social science & medicine* 2013;78:26-33.
14. Damschroder LJ, Aron DC, Keith RE, *et al*. Fostering implementation of health services research findings into practice: a consolidated framework for advancing implementation science. *Implementation science* 2009;4(1):1.
15. Moore GF, Audrey S, Barker M, *et al*. Process evaluation of complex interventions: Medical Research Council guidance. *BMJ* 2015;350:h1258.
16. May CR, Johnson M, Finch T. Implementation, context and complexity. *Implementation Science* 2016;11(1):141.
17. May C. Mobilizing modern facts: health technology assessment and the politics of evidence. *Sociology of Health & Illness* 2006;28(5):513-32.
18. Pham MT, Rajić A, Greig JD *et al*. A scoping review of scoping reviews: advancing the approach and enhancing the consistency. *Research synthesis methods* 2014;5(4):371-385.
19. Davis K, Drey N, Gould D. What are scoping studies? A review of the nursing literature. *International journal of nursing studies* 2009;46(10):1386-1400.
20. Arksey H, O'Malley L. Scoping studies: towards a methodological framework. *International journal of social research methodology* 2005;8(1):19-32.
21. Levac D, Colquhoun H, O'Brien KK. Scoping studies: advancing the methodology. *Implementation Science* 2010;5(1):1.
22. Hsieh H-F, Shannon SE. Three approaches to qualitative content analysis. *Qualitative health research* 2005;15(9):1277-1288.
23. Watson R, Parr J, Joyce C, *et al*. Models of transitional care for young people with complex health needs: a scoping review. *Child Care Hlth Dev* 2011;37(6):780-791.
24. Johnson MJ, May CR. Promoting professional behaviour change in healthcare: what interventions work, and why? A theory-led overview of systematic reviews. *BMJ open* 2015;5(9):e008592.
25. Gallacher K, Morrison D, Jani B, *et al*. Uncovering treatment burden as a key concept for stroke care: a systematic review of qualitative research. *PLoS Med* 2013;10(6):e1001473.
26. Mair FS, May C, O'Donnell C, *et al*. Factors that promote or inhibit the implementation of e-health systems: an explanatory systematic review. *Bulletin of the World Health Organization* 2012;90(5):357-364.
27. May C, Sibley A, Hunt K. The nursing work of hospital-based clinical practice guideline implementation: an explanatory systematic review using Normalisation Process Theory. *International journal of nursing studies* 2014;51(2):289-299.
28. Carey I, Shouls S, Bristowe K, *et al*. Improving care for patients whose recovery is uncertain. The AMBER care bundle: design and implementation. *BMJ supportive & palliative care* 2015;5(1):12-18.
29. Etkind SN, Karno J, Edmonds PM, et al. Supporting patients with uncertain recovery: the use of the AMBER care bundle in an acute hospital. *BMJ supportive & palliative care* 2015;5(1):95-98.
30. Morris M, Briant L, Chidgey-Clark J, *et al*. Bringing in care planning conversations for patients whose recovery is uncertain: learning from the AMBER care bundle. *BMJ supportive & palliative care* 2011;1(1):72-72.
31. Nutt S, Lanaghan K. *Management of Patients who are End of Life*. Doncaster and Bassetlaw Hospitals NHS Foundation Trust, 2015.

For peer review only - http://bmjopen.bmj.com/site/about/guidelines.xhtml

32. Guy's and St Thomas' NHS Foundation Trust. *The AMBER care bundle – a guide for patients, their relatives and carers*. Patient information leaflet, 2012.
33. Fritz Z, Fuld JP. Development of the Universal Form Of Treatment Options (UFTO) as an alternative to Do Not Attempt Cardiopulmonary Resuscitation (DNACPR) orders: a cross-disciplinary approach. *J Eval Clin Pract* 2015;21(1):109-117.
34. *UFTO: Universal Form of Treatment Options* (2015) Available at: www.ufto.org (Accessed 01 July 2015).
35. *Universal Form of Treatment Options: Scientific Background* (2015) Available at: http://www.ufto.org/background/scientific-background/ (Accessed: 01 July 2015).
36. Brimblecombe C, Crosbie D, Lim WK, *et al*. The Goals of Patient Care project: implementing a proactive approach to patient-centred decision-making. *Intern Med J* 2014;44(10):961-966.
37. Hickman SE, Nelson CA, Moss AH, *et al*. Use of the Physician Orders for Life-Sustaining Treatment (POLST) paradigm program in the hospice setting. *J Palliat Med* 2009;12(2):133-141.
38. Hockley J, Watson J, Oxenham D, *et al*. The integrated implementation of two end-of-life care tools in nursing care homes in the UK: an in-depth evaluation. *Palliat Med* 2010;24(8):828-838.
39. Kaldjian LC, Broderick A. Developing a policy for do not resuscitate orders within a framework of goals of care. *Jt Comm J Qual Patient Saf* 2011;37(1):11-19.
40. Mbriwa K, Limaye S. HOW CAN WE IMPROVE 'DO NOT ATTEMPT CARDIOPULMONARY RESUSCITATION' (DNACPR) AND END OF LIFE CARE (EOLC) DECISION MAKING? *Age and Ageing* 2014:43(suppl_1): i10.
41. Newport KB, Patel S, Lyckholm L, *et al*. The "PSOST": Providers' Signout for Scope of Treatment. *J Palliat Med* 2010;13(9):1055-1058.
42. Piers RD, Benoit DD, Schrauwen WJ, *et al*. Do-not-resuscitate decisions in a large tertiary hospital: differences between wards and results of a hospital-wide intervention. *Acta Clinica Belgica* 2014;66:2,116-122.
43. Stockdale C, Trivedi B, Jerome E, *et al*. Implementation of a combined Cardiopulmonary Resuscitation and Treatment Escalation Plan document in a District General Hospital. *BMJ Quality Improvement Reports* 2013;2(2):u202653. w201236.
44. Dahill M, Powter L, Garland L, et al. Improving documentation of treatment escalation decisions in acute care. BMJ quality improvement reports 2013;2(1):u200617. w201077.
45. Powter L, Dahill M, Garland L, *et al. Improving decision making and documentation of resuscitation and ceiling of treatment decisions*. Available at: http://www.acutemedicine.org.uk/wp-content/uploads/2012/10/aqi28%20-%20improving%20decision%20making%20and%20documentation%20of%20resuscitation%20and%20ceiling%20of%20treatment%20decisions.pdf (Accessed 27 June 2016).
46. Mercer MH. The death of DNR: Treatment escalation plans. (Letter to the editor). *BMJ* 2009;338:b2020.
47. Obolensky L, Mercer M. (2007) Replacing Do-Not-Resuscitate Forms with Treatment Escalation Plans. *The Intensive Care Society Annual Spring Meeting*.
48. Obolensky L, Mercer M. (2007) Patients' Experiences of Treatment Escalation Plans. *The Intensive Care Society Annual Spring Meeting.*
49. Karakusevic C. *Devon Treatment Escalation Plan & Resuscitation Decision Record (TEP).* Available at: http://www.macmillan.org.uk/documents/aboutus/health_professionals/ primarycare/showcasing/2013/treatment-escalation-and-resuscitation-devon.pdf (Accessed: 23 June 2016).
50. Gray C. *Treatment Escalation Plans (TEP) and Resuscitation Decision Records*. Torbay and Southern Devon Health and Care NHS Trust, 2014.

For peer review only - http://bmjopen.bmj.com/site/about/guidelines.xhtml

51. Care Quality Commission. *North Devon Healthcare NHS Trust End of Life Care Quality Report*. End of Life Care Quality Report, 2014.
52. Human S, Scott K. *Treatment Escalation Plan (TEP) Forms.* Rowcroft Hospice, 2013.
53. Devon LMC Newsletter. *Treatment Escalation Plans (TEP) – new update*. Newsletter article, August 2013.
54. Hill S, Mitchell P, Cooper J, *et al*. *New TEP form (Version 10) – Frequently Asked Questions*. Macmillan Cancer Support, St Luke's Hospice Plymouth, East and West Devon Clinical Commissioning Group.
55. Diamond A. *Briefing note on the use of Treatment Escalation Plans (TEPs).* North Devon Healthcare NHS Trust.
56. North Devon Healthcare NHS Trust. *Guidance for completing Treatment Escalation Plan and Resuscitation Decisions*. Available at: http://www.northdevonhealth.nhs.uk/wp-content/uploads /2015/07/V10-FINAL-Guidance-for-TEP-and-Flowchart.pdf (Accessed 21 March 2017).
57. Paes P, O'Neill C. Treatment escalation plans–a tool to aid end of life decision making? *BMJ Supportive & Palliative Care* 2012;2(Suppl 1):A60-A60.
58. Paes P. *Treatment Escalation Planning - Introducing systematic senior clinical decision making.* Available at: http://www.karenpicking.co.uk/images/Paul%20Paes%20-%20project %20poster.pdf (Accessed: 23 June 2016).
59. Hannah J. (2014) *One TEP at a time.... Treatment Escalation Plans in Medical High Dependency.* Available at: http://www.qihub.scot.nhs.uk/media/618301/ julie%20hannah.pdf (Accessed: 08 March 2017).
60. Thomson B, Kok K, Harrod E, *et al*. *Introducing Treatment Escalation Plans in Acute Medicine*. Available at: http://www.acutemedicine.org.uk/wp-content/uploads/2014/10/AQI-40-Introducing-Treatment-Escalation-Plans-in-Acute-Medicine-A-Quality-Improvement-Project.pdf (Accessed: 23 June 2016).
61. Over J. *Treatment Escalation Plan and Resuscitation Decision Record (in relation to the adult patient over 18 years)*. Royal Cornwall Hospitals NHS Trust, 2015.
62. West Suffolk NHS Foundation Trust Resuscitation Committee. *Escalation Plan and Resuscitation Status (EPARS) (including DNACPR).* West Suffolk NHS Foundation Trust, 2013.
63. Basildon and Thurrock University Hospitals NHS Foundation Trust Critical care. *Treatment Escalation Plan (TEP*). Basildon and Thurrock University Hospitals NHS Foundation Trust, 2014.
64. Downar J, Hawryluck L. What should we say when discussing "code status" and life support with a patient? A Delphi analysis. *J Palliat Med* 2010;13(2):185-195.
65. Bailey FA, Williams BR, Woodby LL, *et al*. Intervention to improve care at life's end in inpatient settings: the BEACON trial. *J Gen Intern Med* 2014;29(6):836-843.
66. Bookbinder M, Blank AE, Arney E, *et al*. Improving end-of-life care: development and pilot-test of a clinical pathway. *J Pain Symptom Manage* 2005;29(6):529-543.
67. Bernacki RE, Block SD; American College of Physicians High Value Care Task Force. Communication about serious illness care goals: a review and synthesis of best practices. *JAMA Intern Med* 2014;174(12):1994-2003.
68. Hoerger M, Epstein RM, Winters PC, *et al*. Values and options in cancer care (VOICE): study design and rationale for a patient-centered communication and decision-making intervention for physicians, patients with advanced cancer, and their caregivers. *BMC Cancer* 2013;13:188.
69. Markoczy L. Consensus formation during strategic change. *Strategic Management Journal* 2001;22(11):1013-1031.
70. Nugus P, Greenfield D, Travaglia J, *et al*. How and where clinicians exercise power: Interprofessional relations in health care. *Social science & medicine* 2010;71(5):898-909.

For peer review only - http://bmjopen.bmj.com/site/about/guidelines.xhtml

71. Richens Y, Rycroft-Malone J, Morrell C. Getting guidelines into practice: a literature review. *Nursing Standard* 2004;18(50):33-40.
72. Checkland PB. (1981) *Systems thinking, systems practice*. Chichester: John Wiley.
73. Stetler CB, Legro MW, Rycroft-Malone J, *et al*. Role of "external facilitation" in implementation of research findings: a qualitative evaluation of facilitation experiences in the Veterans Health Administration. *Implementation Science* 2006;1(1):1.
74. Locock L, Dopson S, Chambers D, *et al*. Understanding the role of opinion leaders in improving clinical effectiveness. *Social science & medicine* 2001;53(6):745-757.
75. Phillipson L, Goodenough B, Reis S, *et al*. Applying Knowledge Translation Concepts and Strategies in Dementia Care Education for Health Professionals: Recommendations From a Narrative Literature Review. *Journal of Continuing Education in the Health Professions* 2016;36(1):74-81.
76. Field RA, Fritz Z, Baker A, *et al*. Systematic review of interventions to improve appropriate use and outcomes associated with do-not-attempt-cardiopulmonary-resuscitation decisions. *Resuscitation* 2014;85(11):1418-1431.
77. National Institute for Health Research. *Patients and the public*. 2016. Available at: http://www.nihr.ac.uk/patients-and-public/ (Accessed 04 November 2016).
78. May C, Montori VM, Mair FS. We need minimally disruptive medicine. *BMJ* 2009;339:b2803.
79. Shippee ND, Shah ND, May CR, *et al*. Cumulative complexity: a functional, patient-centered model of patient complexity can improve research and practice. *Journal of clinical epidemiology* 2012;65(10):1041-1051.
80. Baker R, Camosso-Stefinovic J, Gillies C, *et al*. Tailored interventions to overcome identified barriers to change: effects on professional practice and health care outcomes. *The Cochrane Library* 2010.
81. Regnard C. Deciding right–the UKs first regional integration of advance care decisions. *BMJ Supportive & Palliative Care* 2012;2(Suppl 1):A53-A54.

For peer review only

For peer review only - http://bmjopen.bmj.com/site/about/guidelines.xhtml

Figure 1. Flow diagram of literature screening

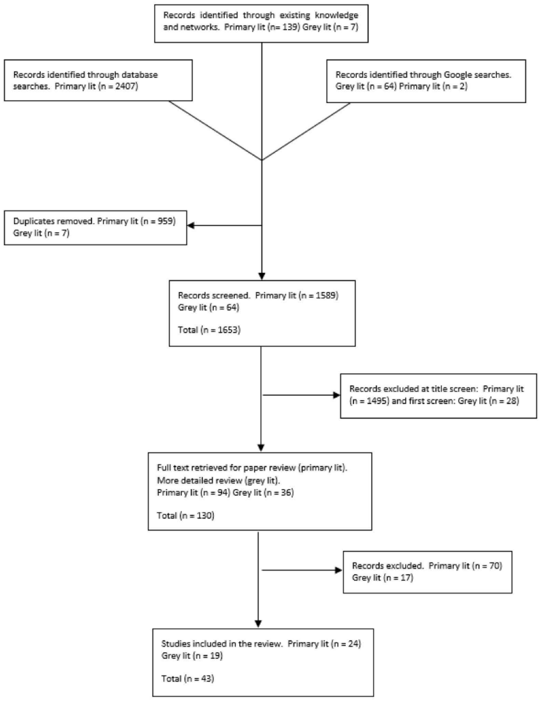

Figure 1: Flow diagram of literature screening

210x297mm (300 x 300 DPI)

Appendix 1: ***Primary literature search strategy***

**Combined key word searches**		**Number of results in MEDLINE**	**3rd key word added if results >200**
End of life care	Goals of care	(298) 12	Treatment decision making
End of life care	Ceiling of care	5	
End of life care	Ceiling of treatment	1	
End of life care	Treatment limitations	12	
End of life care	Resuscitation	(545) 11	Treatment decision making
End of life care	DNACPR	(859) 79	Goals of care
End of life care	Treatment escalation plans	0	
End of life care	Treatment decision making	68	
End of life care	Advance care plans	28	
End of life care	Supported decision making	8	
End of life care	End of life decisions	(624) 4	Treatment limitations for Medline, goals of care for CINAHL
End of life care	Treatment goals	56	
Rapid response	Goals of care	11	
Rapid response	Ceiling of care	0	
Rapid response	Ceiling of treatment	0	
Rapid response	Treatment limitations	5	
Rapid response	Resuscitation	(239) 2	Treatment limitations or End of life decisions
Rapid response	DNACPR	(549) 5	Palliative care
Rapid response	Treatment escalation plans	0	
Rapid response	Treatment decision making	4	
Rapid response	Advance care plans	0	
Rapid response	Supported decision making	0	
Rapid response	End of life decisions	1	
Rapid response	Treatment goals	5	
Medical emergency	Goals of care	53	
Medical emergency	Ceiling of care	0	
Medical emergency	Ceiling of treatment	2	
Medical emergency	Treatment limitations	8	
Medical emergency	Resuscitation	(6,041) 11	End of life decisions
Medical emergency	DNACPR	(2,291) 7	End of life decisions or Treatment goals
Medical emergency	Treatment escalation plans	0	
Medical emergency	Treatment decision making	26	
Medical emergency	Advance care plans	0	
Medical emergency	Supported decision making	4	
Medical emergency	End of life decisions	22	
Medical emergency	Treatment goals	17	
Critical care outreach team	Goals of care	0	
Critical care outreach team	Ceiling of care	0	
Critical care outreach team	Ceiling of treatment	0	
Critical care outreach team	Treatment limitations	0	
Critical care outreach team	Resuscitation	2	
Critical care outreach team	DNACPR	0	
Critical care outreach team	Treatment escalation plans	0	
Critical care outreach team	Treatment decision making	0	
Critical care outreach team	Advance care plans	0	
Critical care outreach team	Supported decision making	0	
Critical care outreach team	End of life decisions	0	

For peer review only

Critical care outreach team	Treatment goals	0	
Palliative care	Goals of care	(633) 27	End of life decisions or Treatment decision making
Palliative care	Ceiling of care	4	
Palliative care	Ceiling of treatment	0	
Palliative care	Treatment limitations	34	
Palliative care	Resuscitation	(599) 54	Goals of care
Palliative care	DNACPR	(2,232) 84	Goals of care
Palliative care	Treatment escalation plans	0	
Palliative care	Treatment decision making	146	
Palliative care	Advance care plans	12	
Palliative care	Supported decision making	3	
Palliative care	End of life decisions	(359) 28	Treatment decision making or Goals of care
Palliative care	Treatment goals	182	
Clinical deterioration	Goals of care	5	
Clinical deterioration	Ceiling of care	0	
Clinical deterioration	Ceiling of treatment	0	
Clinical deterioration	Treatment limitations	1	
Clinical deterioration	Resuscitation	67	
Clinical deterioration	DNACPR	220	N/A (adding 3rd term gave 0 results)
Clinical deterioration	Treatment escalation plans	0	
Clinical deterioration	Treatment decision making	3	
Clinical deterioration	Advance care plans	0	
Clinical deterioration	Supported decision making	1	
Clinical deterioration	End of life decisions	0	
Clinical deterioration	Treatment goals	7	
Treatment escalation plans	--------------------	10	
Advance care plans	--------------------	57	
Treatment decision making	Resuscitation	49	
Treatment limitations	Resuscitation	25	

For peer review only

For peer review only

For peer review only - http://bmjopen.bmj.com/site/about/guidelines.xhtml

Appendix 2: ***Data extraction tool***

ARTICLE DETAILS					
Study Title					
Primary Author					
Year					
Country					
Setting	Community/Population		Nursing home/Residential care		
	Hospital/Secondary		General Practice/Primary care		
	Other (specify):				
Study design					
Participant(s)					
What is the research objective?					
Is the study describing a new/existing decision or communication <u>tool</u>?	Yes	Name of tool:		No	
What are the decisions of interest being studied? (e.g. DNACPR/active medical care/palliative care)	DNACPR		Other (specify):		
	Active Medical Care				
	Supportive/Palliative Care				
What are the core components of the tools/intervention being studied?	Escalation levels/interventions		Record of who plan discussed was discussed		
	Resuscitation status		Indication of person recording discussion and decision		
	Document rationale for treatment decision and resuscitation status		Mental capacity assessment		
	Review/discontinuation date		Prompt to record completion in notes (relating to separate tool only)		
Who makes decisions (e.g. consultant, junior doctors) and who is involved in decision-making (e.g. patients					

For peer review only

and families)?				
What is the trigger to initiating the process of decision making and/or what factors are taken into consideration? (e.g in advance of deterioration/in crisis or emergency situation)	All patients within study setting		Selected patients within study setting	
**Coherence**<br><br>*Sense making work individually and collectively when faced with the problem of operationalizing an intervention*	1) Differentiation: Does the intervention differ, and if so, how is it different from current practices? What work has been undertaken to aid understanding of how the new intervention differs from current practice?			
	2) Communal specification: Do staff have a shared understanding of the purpose of the intervention? How has a shared understanding of the purpose of the intervention been built among staff within the organisation?			
	3) Individual specification: Do staff understand their specific tasks and responsibilities in relation to the intervention? What work has been done to ensure individual tasks and responsibilities are understood. Do individuals understand how the intervention will impact upon the nature of their work?			
	4) Internalization: Do staff understand the potential value of the intervention for their work? What work has been done to promote understanding of the value, benefits and importance of the new intervention among staff and the organisation as a whole?			
**Cognitive participation**<br><br>*Relational work people do to build and sustain a community of practice around a new intervention*	1) Initiation: Are there key individuals identified as those driving the intervention forward?			
	2) Enrolment: What work has been done to promote others to 'buy-in' and engage with the intervention, including training and education? Are people open to working with others in new ways to adopt the intervention?			
	3) Legitimation: Do staff believe that participating in the intervention is a legitimate part of their role? What work has been undertaken to ensure people believe it is right for them to be involved with the intervention?			

For peer review only

	4) Activation: Do people continue to support the intervention? What work has been undertaken to sustain the new intervention in practice and keep people supporting it's use?
**Collective action**<br><br>*The operational work that people do to enable new practices*	1) Interactional workability: Can the intervention be easily integrated into existing work practices? What work has been undertaken to allow the new intervention to integrate with existing staff and organisational practices?
	2) Relational integration: Do individuals feel confident in their own and others' abilities to use the intervention? What work has been undertaken to build knowledge and confidence in using the new intervention? Does the intervention impact upon working relationships and what work has been done to minimise this?
	3) Skill set workability: Has the work (associated with the intervention) been assigned to those with the most appropriate skills? What formed the decision behind how the division of labour was allocated? Has sufficient training given staff the skills to enable them to use the intervention?
	4) Contextual integration: What resources have been provided to support the new intervention in practice? Is the intervention adequately supported at managerial level?
**Reflective monitoring**<br><br>*The appraisal work that people do to assess and understand the ways a new intervention affects them and others around them*	1) Systemization: Has information been collected or are there plans to collect information to determine the usefulness of the intervention through feedback, audit or other means? Have the effects of the intervention been reported back to those involved?
	2) Communal appraisal: Is there communal agreement among staff as to the value of the intervention? Has the worth of the intervention been evaluated collaboratively in formal or informal groups?
	3) Individual appraisal: What is the effect of the intervention on an individuals' workload? Do individuals value the effect it has on their individual work?

For peer review only

	4) Reconfiguration: Has appraisal work lead to attempts to improve or modify the intervention?
Do patients and families understand the purpose of the intervention? What work has been done to increase their understanding?	
What is the work that patients have to do and do they involve others in the work (eg. family members)?	
Has feedback from patient/families been sought? Do they see the value of the intervention?	
What are the types of outcomes measured by the study?	
Other	

For peer review only

Appendix 3: ***The constructs and sub-constructs of NPT [5]***

Core Constructs		Sub-constructs	
**Coherence**	The work required to make sense of:<br>a) an intervention, both individually and collectively as a healthcare professional, clinical care team, service and/or organization.<br>b) what is required to individually and collectively implement the intervention (in this case a tool and/or process to support decision making regarding goals of care in the context of pathophysiological deterioration)	**Differentiation**	Understanding how a new set of practices are different from current practices
		**Communal Specification**	How people work together to build a shared understanding of the aims, objectives and expected benefits of a set of new practices
		**Individual Specification**	Work that will help individuals understand their specific tasks and responsibilities around a set of practices
		**Internalization**	Work to understand the value, benefits and importance of a set of practices
**Cognitive Participation**	The **relational work** required within a clinical care team, service and/or organization to establish and sustain the particular intervention	**Initiation**	Work to drive the new set of practices forward
		**Enrolment**	Organizing or reorganizing of participants in order to collectively contribute to the work involved in new practices
		**Legitimation**	The work of ensuring that participants believe it is right for them to be involved and that they can make a valid contribution
		**Activation**	Actions and procedures needed to sustain a practice and maintain participant involvement
**Collective Action**	The operational **work of organising and preparing the care environment, such as a particular service or organisation, to enact the new practices required to deliver the intervention**	**Interactional Workability**	The interactional work that people do with each other and with other elements of a set of practices, when they seek to operationalise them in everyday settings
		**Relational Integration**	The knowledge work that people do to build accountability and maintain confidence in a set of practices and in each other as they use them
		**Skill set**	The allocation work that underpins the division of

For peer review only

		**Workability**	labour that is built up around a set of practices
		**Contextual Integration**	Managing a set of practices through the allocation of different kinds of resources and the execution of protocols, policies and procedures
**Reflexive Monitoring**	The **appraisal work** that people do to assess the acceptability and utility of the intervention, as well as the outcomes of the intervention and its delivery on those who negotiate and enact it (clinicians, individual patients and their families/carers)	**Systemization**	The work of collecting information to determine the effectiveness and usefulness of a new set of practices
		**Communal appraisal**	Participants working together in formal collaboratives or in informal groups to evaluate the worth of a set of practices
		**Individual appraisal**	Participants in a new set of practices work as individuals to appraise its effects on them and the contexts in which they are set
		**Reconfiguration**	Appraisal work by individuals or groups may lead to attempts to redefine procedures or modify practices

For peer review only

Appendix 4: ***Distribution of themes by NPT sub-construct***

		**Themes**					
**NPT core construct**	**NPT sub-construct**	Input into development	Key clinical proponents	Training and education	Workability/ Functionality	Setting and context	Perceived value and appraisal
Coherence	Differentiation	■			■		
	Communal specification	■		■			■
	Individual specification			■			■
	Internalisation	■				■	■
Cognitive participation	Initiation	■	■			■	
	Enrolment	■	■	■	■	■	■
	Legitimation	■				■	■
	Activation		■	■		■	■
Collective action	Interactional workability	■		■	■	■	
	Relational integration	■		■	■	■	■
	Skill set workability		■	■	■	■	
	Contextual integration		■	■	■		■
Reflexive monitoring	Systemization			■			■
	Communal appraisal						■
	Individual appraisal				■	■	■
	Reconfiguration	■		■	■	■	■

For peer review only

PRISMA 2009 Checklist

Section/topic	#	Checklist item	Reported on page #
**TITLE**			
Title	1	Identify the report as a systematic review, meta-analysis, or both.	1
**ABSTRACT**			
Structured summary	2	Provide a structured summary including, as applicable: background; objectives; data sources; study eligibility criteria, participants, and interventions; study appraisal and synthesis methods; results; limitations; conclusions and implications of key findings; systematic review registration number.	2
**INTRODUCTION**			
Rationale	3	Describe the rationale for the review in the context of what is already known.	3-4
Objectives	4	Provide an explicit statement of questions being addressed with reference to participants, interventions, comparisons, outcomes, and study design (PICOS).	3
**METHODS**			
Protocol and registration	5	Indicate if a review protocol exists, if and where it can be accessed (e.g., Web address), and, if available, provide registration information including registration number.	N/A for scoping reviews
Eligibility criteria	6	Specify study characteristics (e.g., PICOS, length of follow-up) and report characteristics (e.g., years considered, language, publication status) used as criteria for eligibility, giving rationale.	5
Information sources	7	Describe all information sources (e.g., databases with dates of coverage, contact with study authors to identify additional studies) in the search and date last searched.	5
Search	8	Present full electronic search strategy for at least one database, including any limits used, such that it could be repeated.	5 & appendix 1
Study selection	9	State the process for selecting studies (i.e., screening, eligibility, included in systematic review, and, if applicable, included in the meta-analysis).	4-6
Data collection process	10	Describe method of data extraction from reports (e.g., piloted forms, independently, in duplicate) and any processes for obtaining and confirming data from investigators.	6
Data items	11	List and define all variables for which data were sought (e.g., PICOS, funding sources) and any assumptions and simplifications made.	6 & appendix 2
Risk of bias in individual studies	12	Describe methods used for assessing risk of bias of individual studies (including specification of whether this was done at the study or outcome level), and how this information is to be used in any data synthesis.	N/A in scoping

For peer review only - http://bmjopen.bmj.com/site/about/guidelines.xhtml

PRISMA 2009 Checklist

Section/topic	#	Checklist item	Reported on page #
			review
Summary measures	13	State the principal summary measures (e.g., risk ratio, difference in means).	6
Synthesis of results	14	Describe the methods of handling data and combining results of studies, if done, including measures of consistency (e.g., I^2) for each meta-analysis.	6

Section/topic	#	Checklist item	Reported on page #
Risk of bias across studies	15	Specify any assessment of risk of bias that may affect the cumulative evidence (e.g., publication bias, selective reporting within studies).	23
Additional analyses	16	Describe methods of additional analyses (e.g., sensitivity or subgroup analyses, meta-regression), if done, indicating which were pre-specified.	N/A
**RESULTS**			
Study selection	17	Give numbers of studies screened, assessed for eligibility, and included in the review, with reasons for exclusions at each stage, ideally with a flow diagram.	7 & figure 1
Study characteristics	18	For each study, present characteristics for which data were extracted (e.g., study size, PICOS, follow-up period) and provide the citations.	7-13
Risk of bias within studies	19	Present data on risk of bias of each study and, if available, any outcome level assessment (see item 12).	14-20
Results of individual studies	20	For all outcomes considered (benefits or harms), present, for each study: (a) simple summary data for each intervention group (b) effect estimates and confidence intervals, ideally with a forest plot.	14-20
Synthesis of results	21	Present results of each meta-analysis done, including confidence intervals and measures of consistency.	14-20
Risk of bias across studies	22	Present results of any assessment of risk of bias across studies (see Item 15).	23
Additional analysis	23	Give results of additional analyses, if done (e.g., sensitivity or subgroup analyses, meta-regression [see Item 16]).	14-20
**DISCUSSION**			
Summary of evidence	24	Summarize the main findings including the strength of evidence for each main outcome; consider their relevance to key groups (e.g., healthcare providers, users, and policy makers).	21-23
Limitations	25	Discuss limitations at study and outcome level (e.g., risk of bias), and at review-level (e.g., incomplete retrieval of identified research, reporting bias).	23-24
Conclusions	26	Provide a general interpretation of the results in the context of other evidence, and implications for future research.	24
**FUNDING**			

For peer review only

PRISMA 2009 Checklist

Funding	27	Describe sources of funding for the systematic review and other support (e.g., supply of data); role of funders for the systematic review.	24

From: Moher D, Liberati A, Tetzlaff J, Altman DG, The PRISMA Group (2009). Preferred Reporting Items for Systematic Reviews and Meta-Analyses: The PRISMA Statement. PLoS Med 6(6): e1000097. doi:10.1371/journal.pmed1000097

For more information, visit: **www.prisma-statement.org**.

For peer review only

For peer review only - http://bmjopen.bmj.com/site/about/guidelines.xhtml

Implementing communication and decision-making interventions directed at goals of care: a theory led scoping review

Journal:	*BMJ Open*
Manuscript ID	bmjopen-2017-017056.R1
Article Type:	Research
Date Submitted by the Author:	27-Jul-2017
Complete List of Authors:	Cummings, Amanda; University of Southampton Faculty of Health Sciences,<br>Lund, Susi; University of Southampton,<br>Campling, Natasha; University of Southampton Faculty of Health Sciences<br>May, Carl; University of Southampton, Faculty of Health Sciences<br>Richardson, Alison; University of Southampton, Faculty of Health Sciences<br>Myall, Michelle; University of Southampton, Faculty of Health Sciences
**Primary Subject Heading**:	Health services research
Secondary Subject Heading:	Communication, Health policy, Palliative care, Emergency medicine
Keywords:	Goals of Care, Ceilings of Care, Treatment Escalation Plans, Decision Making, Normalisation Process Theory, Implementation Science

SCHOLARONE™ Manuscripts

For peer review only

Implementing communication and decision-making interventions directed at goals of care: a theory led scoping review

Amanda Cummings[1,2], Susi Lund[1,2], Natasha Campling[1,2], Carl May[1,2,3], Alison Richardson[1,2,3], Michelle Myall[1,2]

1. Faculty of Health Sciences, University of Southampton, UK
2. NIHR CLAHRC Wessex, University of Southampton, UK
3. University Hospital Southampton NHS Foundation Trust, Southampton, UK

Corresponding author: Dr Amanda Cummings, Faculty of Health Sciences, University of Southampton, Building 67, University Road, Highfield, Southampton, SO17 1BJ
Tel: +44 (0)23 8059 8250; Fax +44 (0)23 8059 8308; Email: ac2a13@soton.ac.uk

Key words: goals of care; ceilings of care; treatment escalation plans; decision-making; implementation science; Normalisation Process Theory; scoping review

Word count: 4,667 (excluding tables and references)

For peer review only - http://bmjopen.bmj.com/site/about/guidelines.xhtml

ABSTRACT

Objectives: To identify the factors that promote and inhibit the implementation of interventions which improve communication and decision-making directed at goals of care in the event of acute clinical deterioration.

Design and methods: A scoping review was undertaken based on the methodological framework of Arksey and O'Malley for conducting this type of review. Searches were carried out in MEDLINE and CINAHL to identify peer reviewed papers and in Google to identify grey literature. Searches were limited to those published in the English language from 2000 onwards. Inclusion and exclusion criteria were applied, and only papers that had a specific focus on implementation in practice were selected. Data extracted were treated as qualitative and subjected to directed content analysis. A theory informed coding framework using Normalisation Process Theory (NPT) was applied to characterise and explain implementation processes.

Results: Searches identified 2,619 citations, 43 of which met the inclusion criteria. Analysis generated six themes fundamental to successful implementation of goals of care interventions: (1) input into development; (2) key clinical proponents; (3) training and education; (4) intervention workability and functionality; (5) setting and context; and (6) perceived value and appraisal.

Conclusions: A broad and diverse literature focusing on implementation of goals of care interventions was identified. Our review recognised these interventions as both complex and contentious in nature making their incorporation into routine clinical practice dependent on a number of factors. Implementing such interventions present challenges at individual, organisational and systems levels, which make them difficult to introduce and embed. We have identified a series of factors that influence successful implementation and our analysis has distilled key learning points, conceptualised as a set of propositions, we consider relevant to implementing other complex and contentious interventions.

Strengths and limitations of this study

- This paper outlines a scoping review of a broad and diverse literature, both published and grey, focusing on the implementation of goals of care interventions for patients facing clinical deterioration. However, because of its focus on implementation, not all examples of goals of care interventions could be included.
- Normalization Process Theory (NPT) was used to investigate and explain the successful implementation of interventions, and a theoretical approach has been applied to all stages of the review process.
- In a number of included papers information relating to implementation barriers was missing and there was bias towards the presentation of positive outcomes. This may reflect a reluctance to focus on challenges with study authors keen to exhibit and promote the benefits of interventions.
- The review lead to goals of care interventions being defined as 'contentious' with a moral purpose and value, and identified the elements and learnings that could be transferable to other examples of such interventions.
- We have proposed that contentious interventions consist of components at three levels. Across the literature reviewed the focus was on components at the individual level where negotiated decision-making between participants occurs. There was limited focus on the components which take place within and across organisations and the influence of system constraints. This has important limitations for our interpretation of data and analysis.

1. INTRODUCTION

For peer review only - http://bmjopen.bmj.com/site/about/guidelines.xhtml

In the event of a patient becoming acutely unwell, treatment and care decisions are recommended by clinicians. While these decisions are based on the clinical judgement of a healthcare professional they should also be bound by the preferences and wishes of the patient and their family. Processes and tools (referred to here as goals of care interventions), that provide a framework for discussing and documenting appropriate treatment options in the event of acute clinical deterioration, are paramount. These interventions aim to improve patient and family involvement, enabling exploration and understanding of the current clinical situation and facilitation of communication and negotiated decision-making about future treatment options [1, 2]. They offer a means for patients' preferences to be taken into account, improving communication and clarity across the wider clinical team.

We refer to goals of care in the event of acute deterioration, where different levels of treatment might be appropriate and range from full escalation in a critical care environment to symptom control measures [3]. Goals of care are currently referred to using various terms, including but not confined to ceilings of care, treatment escalation plans and treatment limitations. They exist in numerous formations including a specific, dedicated paper form, a narrative entry in a paper medical record and inclusion in an electronic patient record, and may be introduced as an extension of the 'do not attempt cardio-pulmonary resuscitation' (DNACPR) process. These interventions offer a system for recording recommended treatment and care, ideally including all components of the decision pathway and can be applied across different care settings. They require a process to be created and implemented that is recognised across organisations, takes account of socio-legal frameworks, such as the UK Mental Capacity Act (MCA) (2005) [4], and is designed to protect individuals who do not have capacity to make decisions about their care and treatment.

Goals of care interventions are complex and consist of multiple interacting components. The number and difficulty of behaviours required by those delivering or receiving such an intervention, variability of outcomes, and degree of flexibility and tailoring that is permitted, all contribute to making this a complex intervention [5]. As well as being complex, goals of care interventions are established as interactions and recording systems with a moral purpose and value. This means they may be *contentious* in practice because they contain elements which seek to routinise highly complex clinical skills, practice and different types of wisdom, in a context of uncertainty. Contentiousness can arise because the intervention relies on patient, family and clinician interactions, inter-clinician interactions (potentially across clinical settings and organisational boundaries), and societal and legal frameworks, such as the MCA (2005) and the European Convention on Human Rights (ECHR) (2002) [4, 6].

In spite of a growing body of literature describing the introduction and benefits of goals of care interventions, little is known about the factors which influence their successful implementation in clinical practice. Here implementation is defined as 'any deliberately initiated attempt to introduce new, or modify existing, patterns of action in health care or some other formal organizational setting. Deliberate initiation means that an intervention is: institutionally sanctioned; formally defined; consciously planned; and intended to lead to a changed outcome' [7]. As we have previously argued, "this is more than the adoption or diffusion of innovations" as effective implementation is about interventions being made workable and embedded in routine clinical practice [7].

For peer review only - http://bmjopen.bmj.com/site/about/guidelines.xhtml

Understanding and evaluating the implementation of complex interventions in practice remains a challenge for healthcare managers, policy makers and for those who enact them outside of formal research settings [8]. Furthermore, as interventions found to be effective in the context of health services research studies can fail to translate into meaningful healthcare outcomes across varying contexts [9] this makes understanding the reasons for failure or partial success even more essential. Implementation science, which promotes the integration of research findings and evidence into healthcare policy and practice, is increasingly recognised within health services research to make a key contribution to such knowledge. Comprehensive process evaluation of the implementation of healthcare interventions is increasingly important for future learning [10], as it enables understanding of transition from closed systems of highly structured research or service development projects into the real world of open systems healthcare delivery where they are operationalised [11, 12]. Learning from the available existing knowledge in this area can be used to inform healthcare practice change and contribute to the field of implementation science.

Having previously applied Normalisation Process Theory (NPT) to aid learning in the comparable context of advance care plans [13], we have used NPT to characterise and explain implementation processes [14]. NPT provides a set of tools to investigate and understand the processes through which interventions are operationalised in healthcare settings and incorporated into everyday practice [14-17].

In this paper, we present a scoping review of goals of care interventions which aims to: *identify the factors that promote and inhibit the implementation of interventions which improve communication and decision-making directed at goals of care.* Using goals of care interventions as an example, a secondary aim of this review is to: *characterise the components and consider the implications for implementation of, contentious interventions*.

2. DESIGN AND METHODS

A scoping review was the most appropriate methodology, given the need to extract and map principles from a diverse and broad body of evidence [18, 19].

We used Arksey and O'Malley's five stage framework for conducting scoping reviews which includes: identifying the research question, identifying relevant literature, selection, charting the data and collating, summarising and reporting the results [20]. This guided the scoping review and where necessary we developed more specific procedures to inform the review process. Levac et al's recommendations for refining the methodological application were also incorporated to increase rigour of the review process [21].

Inclusion and exclusion criteria

Literature was selected using specific inclusion and exclusion criteria (Table 1), and was included if it was considered to have a specific focus on implementation in practice. To identify all relevant literature on goals of care, grey literature was included and actively sought as part of the search strategy. Only papers published from the year 2000 onwards were included as those published earlier were unlikely to reflect current practice. Papers focusing solely on DNACPR orders were excluded due to their focus on only one

For peer review only - http://bmjopen.bmj.com/site/about/guidelines.xhtml

decisional element of goals of care. Existing evidence also suggests that DNACPR decisions are not always discussed with patients or families [22]. The combining of DNACPR decisions within wider goals of care interventions has been shown to improve clarity and communication, and the focus of this review is on implementing a process whereby goals of care are discussed [23]

Table 1. Inclusion and exclusion eligibility criteria

Inclusion criteria	Exclusion criteria
Literature describing implementation of interventions related to communication and decision-making around goals of care.<br>Studies with adult patients in hospital and community settings.<br>Studies involving end of life care, clinical deterioration and clinically uncertain outcomes.<br>Studies published in the English language.<br>Papers published between 2000-2015.<br>Qualitative and quantitative studies, including clinical trials and RCTs.<br>Published conference abstracts/conference related papers<br>Grey literature (limited to: policies, reports, research posters, patient/staff guidance, websites).	Papers not describing an intervention (process or tool).<br>Papers reporting treatment effectiveness.<br>Papers describing DNACPR orders only.<br>Studies in neonatal and paediatric settings.<br>Studies involving brain stem death.<br>Studies using biomedical data and drug trials.<br>Non-English language studies<br>Papers published before 1 January 2000.

Search strategy and information sources

The search strategy was designed to identify primary studies and other literature, both published and unpublished, which met the eligibility criteria. Separate searches were undertaken for primary and grey literature.

Primary literature searches

The primary literature search was carried out in two stages. The first stage involved an initial search of the bibliographic database MEDLINE using a preliminary key word search based on the terms of the topic, the text terms used in titles and abstracts, and index terms used to describe articles. Full details of the primary literature search strategy are outlined in appendix 1.

Terms identified in the titles and abstracts of relevant articles produced from stage 1 were used to develop further key words for the second stage of the literature search (see appendix 1). Dual combined key word searches were conducted in MEDLINE and CINAHL bibliographic databases, with a third key word added if searches produced >200 results. In addition, further literature were identified through existing knowledge and networks.

Grey literature searches

For peer review only - http://bmjopen.bmj.com/site/about/guidelines.xhtml

Google searches were conducted using terminology relating to known goals of care interventions identified in an earlier scoping exercise which mapped the use of forms for recording these decisions in the UK. The first 10 results were screened for relevance and further review. A further Google search focused on policies and guidance related to goals of care was undertaken and the first 50 results screened for relevance.

All screened, de-duplicated citations were imported into the bibliographic software management package Endnote. Searches were completed by August 2015. This was due to the imminence of a national programme of work which has led to the Recommended Summary Plan for Emergency Care and Treatment (RESPECT)[24]. Communication and decision-making around goals of care is a growing area of interest, and it is hoped that findings from this review can be used to inform the implementation of such a major advancement in the field.

Screening

An extensive screening process was undertaken. At the first stage of screening, articles were assessed by two independent reviewers (AC, CRM) based on the information provided in the title. Primary literature judged to be relevant after first screen, and which met the eligibility criteria, were obtained in full text. Articles for which there was disagreement between reviewers, were also obtained in full text. Full text articles were examined for adherence to the inclusion criteria and then screened (by AC, with input from CRM and MM, in cases of uncertainty or disagreement). Grey literature were title screened for relevance and a further full review examined adherence to inclusion criteria.

Quality assessment

This scoping review included a non-heterogeneous sample of primary and grey literature, which made it difficult to universally apply quality assessment criteria. As standard to most scoping reviews, we did not undertake formal quality assessment and excluded papers only on grounds of relevance. As a result the analytical focus of this review centred on a critique of relevance and contribution of the included literature and did not consider methodological quality.

Data extraction

In line with Arksey and O'Malley's method [20], data extraction (charting) was multi-staged. In the first stage we collected descriptive characteristics from each paper such as study design and setting. In the second stage, findings and discussion sections of included literature were extracted into a data extraction tool (see appendix 2) informed by NPT [15-17].

The data extraction tool was designed to chart specific details of the literature and to understand factors influencing implementation. The tool was piloted on a sample (n=5) of primary and grey literature, with subsequent amendments resulting in the final version. Data was extracted by independent reviewers (AC, SL, MB). Following Levac et al's recommendations, two reviewers (AC, MM) independently extracted data for a 30% sample of primary literature to ensure approaches to extraction were consistent with each other and with the research aims [21].

For peer review only - http://bmjopen.bmj.com/site/about/guidelines.xhtml

Data analysis

A two-stage analytical process was undertaken. During the first stage, data extracted were treated as qualitative data and analysed using directed content analysis [25]. A theory informed coding framework was developed using the four main constructs and sub-constructs of NPT [15, 16]. Data were identified and categorised to the constructs and sub-constructs of NPT, exploring barriers and facilitators to implementation.

The use of NPT as a theoretical framework followed its successful application in a number of different healthcare intervention reviews [13, 26-30]. NPT investigates and explains the successful operationalization of interventions: how they become part of everyday practice in healthcare settings. It embodies the different types of 'work' undertaken by individuals around implementing, embedding and integrating, and allows us to understand the social structures and contexts through which new interventions are operationalised [14-17]. In relation to implementation of goals of care interventions, definitions of the four core constructs and sub-constructs of NPT used in this review are outlined in appendix 3.

During the second stage, thematic analysis of the literature content was undertaken to identify, characterise and explain the factors that shape implementation of interventions that initiate communication and decision-making around goals of care. A final higher level of analytical interpretation followed, to characterise the components of contentious interventions, and generate transferable learning outcomes for their implementation.

3. RESULTS

Searches identified 2619 citations. Following de-duplication and relevance screening, 43 sources of literature (relating to 23 interventions), including 24 items of published literature (e.g. peer-reviewed papers, conference abstracts) and 19 items of grey literature (e.g. conference posters, patient information documents), met the inclusion criteria and were included for data extraction (see figure 1). Table 2 provides a summary of the characteristics of included literature, the range of interventions described and the decisions of interest addressed, including DNACPR, goals of active care, supportive or palliative care and those which are limited to communication guidelines only.

Insert Figure 1. Here

For peer review only - http://bmjopen.bmj.com/site/about/guidelines.xhtml

Table 2. Summary of included literature

Name and description of intervention	Decisions of interest: DNACPR	Decisions of interest: Ceilings of active care	Decisions of interest: Supportive/Palliative Care	Decisions of interest: Communication guidelines around goals of care	Author & Year	Country & Healthcare Setting	Literature Type	Study Design & Methods	Study Objective
AMBER care bundle<br><br>Care bundle consisting of a proforma and sticker.<br>Promotes consistent communication and care planning for patients identified as facing uncertain recovery.	X	X			Carey et al 2014[31]	UK Hospital (5 acute wards)	*Published peer-reviewed study*	Descriptive/Quantitative: Overview of development and implementation of AMBER, and data on clinical impact.	To describe the design, development and implementation of the AMBER care bundle and its impact on clinical service.
					Etkind et al 2014[32]	UK Hospital (5 acute wards)	*Published peer-reviewed study*	Quantitative: Retrospective electronic case note review of patients who died in wards where AMBER was implemented.	To evaluate application of the AMBER care bundle in a UK hospital and describe factors affecting its use.
					Morris et al 2011[33]	UK Hospital	*Published conference abstract*	Descriptive: Overview of AMBER and its implementation.	Learning points from implementation of the AMBER care bundle.
					Doncaster and Bassetlaw Hospitals NHS Foundation Trust 2015[34]	UK NHS Trust	*Grey literature*	Policy Document	Management of patients who are at end of life.
					Guy's and St Thomas' NHS Foundation Trust 2012[35]	UK NHS Trust	*Grey literature*	Patient Guidance	The AMBER care bundle – a guide for patients, their relatives and carers.
Universal Form Of Treatment Options (UFTO)	X	X	X		Fritz et al 2013[3]	UK Hospital	*Published peer-reviewed*	Prospective mixed method:	To determine whether the introduction UFTO reduces harms in

For peer review only - http://bmjopen.bmj.com/site/about/guidelines.xhtml

Form contextualising the CPR decision within overall treatment plans.<br>Includes documentation and discussion of care and treatment decisions.						(2 acute wards)	*study*	Case note review of DNACPR patients, qualitative interviews and observations .	patients in whom a DNACPR was made, and to understand the mechanisms for observed change.
					Fritz et al 2015[36]	UK Hospital (2 acute wards)	*Published peer-reviewed study*	Expert Consensus: Cross-disciplinary adapted Delphi method.	To describe a cross-disciplinary approach to developing a universal form of treatment options as an alternative to DNACPR orders.
					www.ufto.org Accessed: 01/07/15[37]	UK	*Grey literature*	Website	UFTO: Universal form of treatment options
					www.ufto.org/background/scientific-background Accessed 01/07/15[38]	UK	*Grey literature*	Website	UFTO: Scientific background
Goals of Patient Care Summary (GOPC)<br><br>Form to summarise goals of care. Serves as a guide for treatment decisions as the patient's condition improves, stabilises, or deteriorates.	X	X	X		Brimblecombe et al 2014[39]	Australia Hospital (adult medical inpatients)	*Published peer-reviewed study*	Quantitative: Cross-sectional review of medical inpatients and audit of inpatients requiring emergency medical review.	To review the implementation of the GOPC: assess GOPC uptake, quantify completion and review outcomes of the GOPC assessment.
Physician Orders for Life-Sustaining Treatment (POLST)<br><br>Form to ensure that patient wishes regarding life-sustaining treatments are honoured.<br>Converts treatment preferences into medical orders.	X	X	X		Hickman et al 2009[40]	USA Hospice (hospices in 3 US states)	*Published peer-reviewed study*	Descriptive/Quantitative: Survey of hospice staff members. Case note review of deceased patients at subsample of POLST-using hospices.	To evaluate use of POLST by hospice programs, attitudes of hospice staff toward POLST, the effect of POLST on the use of life-sustaining treatments, and the treatment options selected by patients.
Gold Standards Framework for Care Homes (GSFCH) and an adapted Liverpool Care Pathway for Care Homes (LCP)<br><br>The implementation of two end-of-life care tools: the GSFCH supportive/palliative care register	X	X	X		Hockley et al 2010[41]	UK Nursing Home (7 nursing homes in Scotland)	*Published peer-reviewed study*	Quantitative/In-depth Evaluation: Overview of implementation, retrospective case note review of deceased residents, and staff audit.	To evaluate the effectiveness of a high facilitation implementation on professional practices and residents outcomes.

For peer review only

For peer review only - http://bmjopen.bmj.com/site/about/guidelines.xhtml

and an adapted Liverpool Care Pathway for Care Homes.									
UIHC (University of Iowa Hospital and Clinics) Policy Regarding Do Not Resuscitate Orders and End of Life Discussions with Hospitalised Patients<br><br>DNR order policy placing CPR discussions within a framework of end of life discussions, and within a larger goal orientated context.	X	X	X		Kaldjian et al 2011[42]	USA Hospital (academic medical centre)	*Published peer-reviewed study*	Descriptive: Overview of the integration of goals of care into staff education and revision of hospital DNR policy. Literature review and survey of hospitalised adults.	To describe the background and rationale for DNR policy revision and development. To describe the policy itself and ongoing implementation efforts.
A revised DNACPR form, decision making tools and updated CPR guidelines	X	X	X		Mbriwa et al 2014[43]	UK Hospital (elderly and acute medical inpatients)	*Published conference abstract*	Quantitative: Prospective case note audit.	To assess improvement in CPR decision making and ceiling of care documentation/discussions since the introduction of a revised DNACPR form, decision making tools and guidelines.
Providers Signout for Scope of Treatment (PSOST)<br><br>A daily handover tool documenting resuscitation status, goals of care, desired and undesired interventions, in addition to standard handover information.	X	X	X		Newport et al 2010[44]	USA Palliative Care (inpatient unit)	*Published peer-reviewed study*	Descriptive: Overview of tool development, implementation and utility.	To describe the PSOST tool and evaluate its implementation and use in a palliative care unit.
An intervention informing clinicians of the laws on Patients' Rights and the importance of advanced care planning, alongside a new DNR form<br><br>Adapted DNR form, putting emphasis on the motivation, participants involved and communication of the decision-making process.	X	X			Piers et al 2011[45]	Belgium Hospital (adult inpatients)	*Published peer-reviewed study*	Quantitative: Prospective observational study of deceased inpatients pre and post-intervention. Cross-sectional study of patients admitted to non-intensive wards.	To compare the quality of end-of-life care and DNR decisions between hospital wards and after a hospital-wide intervention to improve advanced care planning.
Treatment Escalation Plan and Resuscitation Decision (TEP-CPR)<br><br>Treatment escalation plan tool, to be	X	X	X		Stockdale et al 2013[46]	UK Hospital (2 acute wards)	*Published peer-reviewed study*	Quantitative/Descriptive: Case note audit and staff survey, followed by overview of TEP-CPR	To develop and test a tool for escalation planning. To identify whether a TEP-CPR could reduce the number of patients receiving

For peer review only

For peer review only - http://bmjopen.bmj.com/site/about/guidelines.xhtml

included in the resuscitation form.								development. Staff survey of OOH treatment in patients with TEP-CPR and controls.	inappropriate treatments out of hours.
Ceiling of Treatment (COT) Form<br><br>Tool for improving decision making and documentation of treatment escalation decisions	X	X	X		Dahill et al 2013[47]	UK Hospital (elderly care wards)	*Published peer-reviewed study*	Quantitative/Descriptive: Overview of COT development and implementation. Pre and post-implementation case note review of treatment escalation decisions and staff feedback survey.	To develop, pilot and implement a tool to improve decision-making and the documentation of treatment escalation decisions.
					Powter et al 2012[48]	UK Hospital (elderly care wards)	*Grey literature*	Poster<br><br>Quantitative/Descriptive: Overview of COT development and implementation. Pre and post-pilot data on decision documentation and staff survey.	To improve decision making and documentation of CPR and ceiling of treatment decisions.
Treatment Escalation Plan (Devon)<br><br>Form outlining what treatment options would be appropriate if a patient were to become acutely unwell.	X	X			Obolensky et al 2010[1]	UK Hospital (2 acute T&O wards)	*Published peer-reviewed study*	Prospective quantitative and qualitative evaluation: Patient/relative interviews to evaluate the TEP process.	To evaluate patient and relative experiences of, and thoughts regarding, the Devon TEP.
					Mercer, M 2009[49]	UK Hospital	*Published letter*	Letter to the editor<br><br>Descriptive: Overview of TEP process.	The death of DNR: Treatment escalation plans.
					Obolensky et al 2007[50]	UK Hospital	*Published Conference abstract*	Descriptive/Quantitative: Overview of TEP process and staff survey.	To evaluate initial impact of the Devon TEP from a clinical staff perspective.
					Obolensky et al 2007[51]	UK Hospital	*Published Conference abstract*	Descriptive/Quantitative: Overview of TEP process and patient survey.	To evaluate patient experiences of the Devon TEP.
					Karakusevic, C [52]	UK Hospital (acute	*Grey literature*	Poster<br><br>Descriptive:	To evaluate implementation of the Devon TEP.

For peer review only

						hospital)		Overview of background to development and TEP implementation.	
					Torbay and Southern Devon Health and Care NHS Trust 2014[53]	UK NHS Trust	*Grey literature*	Policy Document	Treatment escalation plans (TEP) and resuscitation decision records policy.
					Care Quality Commission (CQC) 2014[54]	UK NHS Trust	*Grey literature*	CQC Report	Northern Devon healthcare NHS trust, end of life care quality report.
					Rowcroft Hospice 2013[55]	UK Hospice	*Grey literature*	Patient Guidance	Treatment escalation plan (TEP) forms, public information document.
					Devon Local Medical Committee 2013[56]	UK	*Grey literature*	Staff Guidance	Treatment escalation plans (TEP) – newsletter update article.
					Macmillan Cancer Support, St Luke's Hospice Plymouth & North, East and West Devon CCG[57]	UK Hospice	*Grey literature*	Staff Guidance	New TEP form (version 10) – Frequently asked questions document.
					North Devon Healthcare NHS Trust[58]	UK NHS Trust	*Grey literature*	Staff Guidance	Staff briefing note on the use of treatment escalation plans (TEPs).
					NHS Improving Quality Team 2014[59]	UK	*Grey literature*	Staff Guidance	Guidance for completing treatment escalation plan and resuscitation decisions.
Treatment Escalation Plan (TEP)<br><br>Tool for planning the care of a patient at risk of deterioration.	X	X	X		Paes, P et al 2012[60]	UK Hospital (6 acute specialties)	*Published conference abstract*	Descriptive/Quantitative: Pilot, audit and staff feedback survey.	To evaluate staff feedback and decision-making outcomes before and after TEP introduction.
					Paes, P 2012[61]	UK Hospital (6 acute wards & communit	*Grey literature*	Poster<br><br>Descriptive/Quantitative: Overview of TEP implementation and next	To pilot TEP and evaluate staff feedback, OOH decision-making/discussions and TEP completion outcomes. To determine whether and how, TEP

For peer review only - http://bmjopen.bmj.com/site/about/guidelines.xhtml

						y hospital)		steps. Case note review audit before and after TEP pilot, and staff survey.	should be introduced across the trust.
HDU Specific Treatment Escalation Plan<br><br>Plan for ceiling of treatment and escalation level decision on every patient within 24hrs.	X	X			Hannah, J 2014[62]	UK Hospital (medical HDU)	*Grey literature*	Poster<br><br>Descriptive: Overview of TEP development, implementation and next steps, including informal staff feedback.	To describe and evaluate the process of TEP development and its implementation in a medical high dependency unit.
Treatment Escalation Plan Proforma	X	X	X		Thomson et al 2014[63]	UK Hospital (acute medicine)	*Grey literature*	Poster<br><br>Quantitative: Case note review audit before and after TEP pilot, and staff survey.	To pilot TEP and evaluate staff feedback and DNACPR/TEP documentation outcomes.
Cornwall Treatment Escalation Plan and Resuscitation Decision Record	*X*	*X*			Royal Cornwall Hospitals NHS Trust 2015[64]	UK NHS Trust	*Grey literature*	Policy Document	Treatment escalation plan & resuscitation decision record policy.
Escalation plan and resuscitation status (EPARS)<br><br>Form amalgamating escalation plan and CPR decisions.	X	X	X		West Suffolk NHS Foundation Trust 2013[65]	UK NHS Trust	*Grey literature*	Policy Document	Trust policy and procedure: Escalation plan and resuscitation status (EPARS) (including DNACPR).
Treatment Escalation Plan (TEP)	X	X			Basildon and Thurrock University Hospitals NHS Foundation Trust 2014[66]	UK NHS Trust	*Grey literature*	Patient Guidance	Treatment escalation plan (TEP) patient information.
Content guidelines for discussions of CPR and life-sustaining therapy<br><br>Guidelines for the content of a discussion about resuscitation or goals of care.	X	X		X	Downar et al 2010[67]	Canada Hospital (12 physicians)	*Published peer-reviewed study*	Expert Consensus: Delphi method to develop content guidelines	To develop content guidelines for discussions of CPR and life-sustaining therapy.
A palliative care treatment plan intervention			X		Bailey et al 2014[68]	USA Hospital	*Published peer-reviewed*	Descriptive/Quantitative: Overview of intervention	To evaluate the effectiveness of a multi-modal intervention strategy

For peer review only

Best practice guidance supported by a staff education programme, electronic decision support tool and paper educational tools.						(inpatient units at 6 veteran medical centres)	*study*	and its multi-centre implementation. Pre and post-implementation case note review of EOL care in deceased patients.	to improve the processes and quality of end-of-life care in acute inpatient settings.
The Palliative Care for Advanced Disease (PCAD) pathway<br><br>To improve palliative care for inpatients who are expected to die from advanced disease. Clinical pathway including:<br>- Inter-disciplinary care pathway<br>- Nurses documentation flowsheet<br>- Physicians order sheet with guidelines for symptom management			X		Bookbinder et al 2005[69]	USA Hospital (3 inpatient units)	*Published peer-reviewed study*	Descriptive/Quantitative: Overview of intervention, its development and implementation. Case note review of care outcomes in patients who died before and during implementation, in both intervention and control units.	To describe the development and quality improvement strategy for the implementation of PCAD. To pilot and evaluate the utility of PCAD.
Serious illness conversation guide<br><br>Guide describing key elements to be addressed in patient conversations about serious illness care goals.			X	X	Bernacki et al 2014[70]	USA	*Published peer-reviewed study*	Literature review and synthesis of best practices in conversations about serious illness care goals.	To provide clinicians with practical, evidence-based advice (in the form of a serious illness conversation guide).
The VOICE (Values and Options in Cancer Care) intervention<br><br>Education and coaching communication intervention.<br>To facilitate communication and decision making among oncologists, patients and caregivers.				X	Hoerger et al 2013[71]	USA Oncology Services	*Published peer-reviewed study*	Descriptive: Overview of intervention and its planned implementation as part of an RCT.	To describe the study design and rationale for a patient-centred communication and decision-making intervention for physicians, patients with advanced cancer, and their caregivers

For peer review only

For peer review only - http://bmjopen.bmj.com/site/about/guidelines.xhtml

Thematic analysis of literature content suggested there were six common themes fundamental to the successful implementation of goals of care interventions: input into development, key clinical proponents, training and education, intervention workability and functionality, setting and context, perceived value and appraisal. These are outlined below. The links between the sub-constructs of NPT and these identified themes are outlined in table 3. A summary of results by NPT construct are presented in appendix 4 as a summary and appraisal of the literature.

Input into development

The involvement of clinical staff in development of interventions facilitated the identification of current shortfalls in practice, understanding purpose, and shaping novelty, accessibility and utility of design [36, 41, 46, 47, 71]. Developmental input also assisted in promoting 'buy-in', legitimization and confidence in the intervention [31, 36, 41, 44-48, 62, 67]. While some literature focused on the importance of input from senior, specialist individuals [31, 41, 46, 67], including those within implementation settings [46, 47, 69], for intervention design, multi-disciplinary collaboration in the development process was common across papers [3, 31, 36, 38, 46-48, 52, 62, 65, 67, 69], and helpful for gaining collective knowledge from all potential users [31, 36, 47, 48, 62]. In one paper, individuals who had expressed disagreement with one intervention were actively sought for involvement at each stage of development [36], highlighting how inclusion in development can contribute to overcoming barriers of individual resistance. Reconfiguration of forms and procedures were incorporated in the development and implementation process in a number of initiatives [31, 36, 46, 47, 69]. The benefit of this approach was that it allowed for assessment of factors such as usability, clarity and safety, incorporating feedback into further iterations [36, 46, 69].

Key clinical proponents

Key clinical proponents were assigned to orchestrate and lead implementation within specific sites or settings [41, 54, 68, 69]. They were key resources for attending and cascading training as well as overseeing changes, embedding cultural change and providing mentorship [33, 41, 54, 68, 69]. Their direct contact with intervention instigators appeared important in orientating and maintaining momentum of local leadership, including training and facilitation [41, 68, 69]. Similarly, support from individual senior managers in ensuring compliance with training and interventions, promoting sustained use, as well as acting as catalysts for initial implementation was valuable [41, 54, 64, 68, 69, 71]. Trust level managerial support was also evident through incorporation into policy and protocol [34, 42, 47, 53, 64, 65] and approval from directors [48, 62]. Managerial backing could be influential for organisational change, although in reality, engagement with the management of healthcare organizations was recognised by some for its complexity [41, 69].

Training and education

Training and education encouraged 'buy-in' [3, 31, 36, 41, 42, 48, 53, 54, 68, 69], promoted understanding of tasks and responsibilities [31, 36, 42, 48, 68, 69] and facilitated a shared understanding of purpose [42, 52, 68]. Staff often negatively perceived the increased training requirement [36], inclusion within established working practices [42, 48, 68, 69] and feasibility of incorporation into workload [69, 71] were important considerations. Repeated sessions were used to train rotational/part-time staff, address staff turnover and enable sustained engagement [31, 47, 48, 69]. Tailored education based on evidence and

For peer review only - http://bmjopen.bmj.com/site/about/guidelines.xhtml

feedback facilitated the most efficacious training, especially since misunderstanding of responsibilities could result in ineffective or reduced use [36, 39, 41, 54, 69]. Training in the application of skills to the intervention was fundamental [64, 68, 69] and without this, regardless of skill level, the completion, interpretation and application of interventions in practice could be inconsistent [40, 52, 54]. Addressing communication skills was highlighted as important in a number of papers [31, 33, 39, 45, 71]. Individuals responsible for training could ensure that as far as possible all staff received necessary training [64, 68, 69]. The provision of guides, algorithms and interactive materials facilitated skill development [68, 71], and patient and carer information materials helped to sustain practice around newly implemented interventions [34-36, 42, 47, 51, 53, 55, 64-66, 68, 69].

Intervention workability and functionality

A frequently adopted approach included using existing programs or frameworks (local or otherwise) as the basis for new intervention development [40, 41, 43-46, 69]. Literature suggested healthcare practices can flex to incorporate interventions designed as part of, or alongside, existing processes [3, 31, 38, 39, 46, 64, 68]. This approach was often more acceptable to staff, promoting incorporation into practice, adherence and behavioural change [3, 38, 39, 46]. Conversely, lack of transferability across healthcare settings was viewed as a barrier to implementation [40, 44]. The workability of interventions was important, with the integral use of guidelines and prompts facilitating intervention utility and accessibility [1, 3, 31, 32, 36, 39, 40, 42, 44, 47-49, 60-65, 67-71]. The use of stickers and brightly coloured forms for insertion into patient medical records promoted easy recognition of interventions [31, 40]. For many, successful incorporation into working practices appeared to rely upon the paper format of tools, their design and usability [1, 36, 49, 60, 64, 65].

Setting and context

Implementation often occurred within limited clinical settings, and the direct relevance of the specialism (to the intervention) was highlighted as important for promoting its value [32, 44-46, 60, 61, 63, 69]. Subsequent diffusion from implementation setting to hospital-wide acceptance was demonstrated in relation to a number of interventions [1, 31, 36, 69]. Despite this, multiple barriers and enablers to change were identified including: transferability across primary and secondary care settings [40, 64], staff turnover and management stability [41], availability of staff for updates, counter signatories to meet completion timeframes [39, 40, 44], time for communication processes [46], robustness of implementation sites [41, 69], and the difficulty in transforming clinician attitudes [45, 62, 69].

The clinical complexity and unpredictability surrounding the patients for whom the interventions were targeted often affected successful or opportune application [32, 46]. Utility was improved where deterioration was predictable [31, 32, 46], yet recognition of deterioration was on occasion inherently difficult [32]. Clinical uncertainty meant that at times interventions were not fully utilised for the patients they were designed to support [31, 46].

Perceived value and appraisal

Staff perceptions and the degree of alignment with the intended purpose of interventions, were infrequently reported [36, 62, 69]. Staff valuing the intervention from the outset had important implications for effective application [36, 39, 42, 46] and perceived utility, existing supportive local policy

For peer review only - http://bmjopen.bmj.com/site/about/guidelines.xhtml

and inclusive collaborative development were all highlighted as facilitators [36, 38, 39, 41, 46, 62, 65, 67, 69, 71]. At an organisational level, responsiveness to national guidance and policy was likely to promote value [3, 41, 65]. Understanding objectives early on in the implementation pathway was key, as misunderstandings were not uncommon [31, 39, 52], and could lead to reduced or incomplete application of interventions [31, 39].

Following implementation, appraisal of intervention value was frequent [3, 40, 41, 46-48, 50, 60, 61, 63, 69], often demonstrating communal agreement centred around positive impact on working practices [3, 33, 40, 41, 44, 46-48, 50, 60-62, 69]. Increased workload was expressed as a concern by staff, mainly relating to the completion of tools and the need for patient or family discussions [1, 3, 44, 60, 61, 69]. On appraisal, interventions were perceived to be a worthwhile investment due to patient benefit and the improved clarity and time saved, as a result of decisions and discussions taking place earlier in the care trajectory [1, 3, 61]. A number of papers recommended or proposed outcome measures relating to patient and relative experience [3, 31, 40, 47, 48, 62, 69, 71], but only a small number reported utilising such measures [1, 33, 49, 51].

For peer review only

For peer review only

For peer review only - http://bmjopen.bmj.com/site/about/guidelines.xhtml

Table 3. Distribution of themes by NPT construct

NPT Core Construct [16]	NPT Sub-Construct and Questions [16]	Themes					
		Input into development	Key clinical proponents	Training and education	Workability/ Functionality	Setting and context	Perceived value and appraisal
Coherence	Differentiation<br>- Does the intervention differ, & if so, how is it different from current practices?<br>- What work has been undertaken to aid understanding of how the new intervention differs from current practice?	■			■		
	Communal specification<br>- Do staff have a shared understanding of the purpose of the intervention?<br>- How has a shared understanding of the purpose of the intervention been built among staff within the organisation?	■		■			■
	Individual specification<br>- Do staff understand their specific tasks & responsibilities in relation to the intervention? What work has been done to ensure they are understood?<br>- Do individuals understand how the intervention will impact upon the nature of their work?			■			■
	Internalisation<br>- Do staff understand the potential value of the intervention for their work?<br>- What work has been done to promote understanding of the value, benefits & importance of the new intervention among staff & the organisation as a whole?	■				■	■
Cognitive participation	Initiation<br>- Are there key individuals identified as those driving the intervention forward?	■	■			■	
	Enrolment<br>- What work has been done to promote others to 'buy-in' & engage with the intervention, including training & education?<br>- Are people open to working with others in new ways to adopt the intervention?	■	■	■	■	■	■
	Legitimation<br>- Do staff believe that participating in the intervention is a legitimate part of their role?<br>- What work has been undertaken to ensure people believe it is right for them to be involved with the intervention?	■				■	■
	Activation<br>- Do people continue to support the intervention?<br>- What work has been undertaken to sustain the new intervention in practice & keep people supporting its use?		■	■		■	■
Collective action	Interactional workability<br>- Can the intervention be easily integrated into existing work practices?<br>- What work has been undertaken to allow the new intervention to integrate with existing staff & organisational practices?	■		■	■	■	
	Relational integration<br>- Do individuals feel confident in their own & others' abilities to use the intervention?<br>- What work has been undertaken to build knowledge & confidence in using the new intervention?<br>- Does the intervention impact upon working relationships & what work has been done to minimise this?	■		■	■	■	■
	Skill set workability<br>- Has the work (associated with the intervention) been assigned to those with the most appropriate skills?<br>- What formed the decision behind how the division of labour was allocated?<br>- Has sufficient training given staff the skills to enable them to use the intervention?		■	■	■	■	
	Contextual integration<br>- What resources have been provided to support the new intervention in practice?<br>- Is the intervention adequately supported at managerial level?		■	■	■		■
Reflexive monitoring	Systemization<br>- Has information been collected or are there plans to collect information to determine the usefulness of the intervention through feedback, audit or other means?			■			■

For peer review only

	- Have the effects of the intervention been reported back to those involved?						
	Communal appraisal<br>- Is there communal agreement among staff as to the value of the intervention?<br>- Has the worth of the intervention been evaluated collaboratively in formal or informal groups?						
	Individual appraisal<br>- What is the effect of the intervention on an individuals' workload?<br>- Do individuals value the effect it has on their individual work?						
	Reconfiguration<br>- Has appraisal work lead to attempts to improve or modify the intervention?						

For peer review only

For peer review only - http://bmjopen.bmj.com/site/about/guidelines.xhtml

4. DISCUSSION

In this review we have identified a broad and diverse literature focusing on the implementation of goals of care interventions. Findings from this review suggest these interventions are both complex and contentious in nature, and as such it is conceivable that what we have learnt here applies to other contentious interventions and processes, for example discharge planning.

Our analysis has led us to characterise the elements that constitute contentious interventions. Using the example of goals of care interventions, we propose that these interventions consist of three components that intersect at three different levels in a system of negotiated interactions:

1. Negotiated decision making between clinicians, patients and family members, which is localised and characterised by its meaning for the individual.
2. The organisational procedure and collective system of making negotiated decisions.
3. The socio-legal constraints of taking account of preferences (including consent and capacity) that define the parameters.

Review findings point to negotiated interaction processes taking place at between individuals. This mediates a set of procedures about how decisions should be made: a set of expectations about what procedures should be done, what negotiations are possible, and what these look like within and across organisations. Socio-legal conceptualisation involves how organisations must deal with these preferences. All of these continuous components interact and affect a patient's care trajectory.

From the six themes which emerged from our analysis, we have generated transferable learning outcomes for the implementation of contentious interventions. These are described below as a series of propositions relevant to goals of care, which we contend may apply across contentious interventions as a whole. These propositions are:

Individuals resist interventions that replicate the work of existing practices.

The value of incorporating interventions into working practices has previously been described [8] and lies in minimising disruption. The adoption of existing intervention formats and integral guidelines was popular, and may act to improve successful compliance and integration through increased staff familiarity and confidence. However there is a fine line, as individuals resist interventions that replicate the work of existing practices [47, 69]. Interventions have to be easily differentiated from other practices to be valued. There needs to be clarity regarding the practices that are discontinued, preventing unnecessary duplication, and clear identification of the benefits of the new intervention. Evidence from this review suggests that high visibility methods (for example a sticker or coloured background) are valued for quick identification and time saving in clinical practice. These are features unique to the paper based nature of tools. Technological infrastructure facilitating electronic access to one record in all contexts will enhance the likelihood of widespread embedding across organisations.

For peer review only - http://bmjopen.bmj.com/site/about/guidelines.xhtml

Contentious interventions are difficult to integrate in environments where there is clinical unpredictability and uncertainty.

Interventions aimed at improving care for patients facing uncertainty can be difficult to integrate due to the very nature of complexity that exists for these patients and their clinicians. Usually the application of skills and techniques introduce order into such situations, with clinicians seeking scripts to work to. However, clinical uncertainty can render these scripts ineffectual, leading to uncertain outcomes. In light of this, studies in this review suggest the importance of the intervention being delivered by a clinician who has an established relationship with the patient and/or knows their situation well [42, 44]. While this represents the ideal, in the reality of clinical practice and a highly pressurised health service, this may prove difficult to achieve.

Legitimacy is established where individuals build a shared understanding of purpose that enables them to attribute value to the intervention.

The use of senior clinicians and managers in the development process is recognised for building a shared vision [72]. However, the potential for authoritative dominance of certain clinicians is recognised [73], and efforts must be made to enable active contributions from key members of junior staff. A high degree of clinical ownership is recognised as important for successful implementation [74]. This is more likely to be achieved if all clinicians, irrespective of seniority, believe they have a role to play and are making a valid contribution [75]. Key clinical proponents are essential in building legitimacy, successful and sustained implementation, especially in the context of organisational instability [76, 77].

Training and education provide the framework for individuals to understand the value of what they do.

Training and education has an important role in facilitating a shared understanding of purpose, addressing both moral and technical aspects, which are important for building value in the intervention. Training and education serves to facilitate this, fostering clarity of value in the intervention.

The importance of training and education is not a new concept. However, there is a lack of agreement regarding optimal delivery methods, which may relate to a need for training to fit within the clinical context. Findings from the review point to the need for multi-modal educational programmes, repetition and timeliness of training, but this should be considered in the context of staff and financial resource implications [78, 79].

Appraisal work is critically important because it is how individuals value the intervention.

The importance of sharing appraisal outcomes, conveying purpose and value to others is evident, as value is often not realised until successful implementation is achieved. However, reporting of this in the literature was limited [47, 54]. In particular, evaluation of patient and family perspectives was notably absent in the papers included in this review. We suggest their inclusion is crucial for imputing value into interventions which seek to include them [80]. Surveys suggest that clinicians will foresee advantages and disadvantages in any proposed intervention [36, 46]. Therefore, sharing appraisal work, which addresses the positives and negatives, may be beneficial in providing a more balanced view of an intervention's value.

For peer review only - http://bmjopen.bmj.com/site/about/guidelines.xhtml

The transfer of contentious interventions to other settings is problematic.

The initial setting can influence implementation success and subsequent transfer to other locations. In our review there is limited evidence of transfer at scale, over multiple geographical sites [68]. There are multiple barriers through which interventions need to filter, including specialisms, care locations, and structural and cultural factors. In addition, groups of clinicians hold different values, skills and knowledge. This introduces problems of discretion, interpretation and enactment within different contexts and may limit scale up and transferability. While not widely addressed by the papers in this review, wider implementation literature suggests the importance of involving patients and caregivers when transferring and implementing interventions beyond formal clinical settings [81, 82].

In summary, improving decision-making around goals of care and ensuring patient preferences are taken into account requires a better understanding of the implementation processes and factors that promote or impede implementation. We have used the exemplar of goals of care interventions to elucidate propositions in relation to the successful implementation of contentious interventions, where success refers to the routine incorporation and embedding of intervention components into everyday practice. We believe these propositions to be transferable and generalizable beyond the remit of goals of care, to other contentious (and complex) healthcare interventions.

5. CONCLUSION

Findings from the review show that whilst such interventions are variable in design and use, there are a series of collective factors that influence successful implementation into routine clinical practice. We recommend that those seeking to introduce goals of care and other contentious interventions, consider the different facets of NPT, and use knowledge of this to develop implementation strategies. The contentious nature of these interventions means that their incorporation into everyday practice is dependent on a number of factors. Building a shared understanding of purpose that enables participants to attribute value to the intervention which is key, and both training and education and appraisal work play an important role in this process. Identifying clinical proponents who are able to, not only drive, but positively influence implementation is essential. Implementing complex and contentious interventions presents challenges which operate at an individual, organisational and systems level. It is these interaction level processes which make an intervention contentious, as well as making it challenging to introduce and embed. Success is more likely to occur, be established and sustained, if due attention is paid to the processes that facilitate operationalisation in the healthcare settings in which implementation occurs.

ACKNOWLEDGEMENTS

This review was funded by the National Institute for Health Research (NIHR) Collaboration for Leadership in Applied Health Research and Care Wessex. The views expressed are those of the authors and not necessarily those of the NHS, the NIHR or Department of Health. The authors would like to thank Dr Mark Banting for his assistance with data extraction in the initial phase of the review.

AUTHOR CONTRIBUTIONS

MM, CM and AR designed the review. AC performed searches. AC, and CM screened titles and abstracts and full paper screening was carried out by AC, MM and CM. Data extraction was performed by AC, MM

For peer review only - http://bmjopen.bmj.com/site/about/guidelines.xhtml

and SL. AC and MM drafted the manuscript with assistance from SL, CM, NC and AR. All authors critically reviewed the manuscript for intellectual content and approved the final version of the paper. AC is guarantor.

FUNDING

This work was supported by the National Institute for Health Research Collaboration for Leadership in Applied Health Research and Care (NIHR CLAHRC) Wessex which is a partnership between Wessex NHS organisations and partners and the University of Southampton. Funders had no role in study design, data collection and analysis, decision to publish or preparation of the manuscript.

DISCLAIMER

The views expressed are those of the author(s) and not necessarily those of the NHS, the NIHR or the Department of Health.

COMPETING INTERESTS

The authors declare no competing interests.

DATA SHARING STATEMENT

No additional data are available.

For peer review only

For peer review only - http://bmjopen.bmj.com/site/about/guidelines.xhtml

REFERENCES

1. Obolensky L, Clark T, Matthew G, Mercer M. A patient and relative centred evaluation of treatment escalation plans: a replacement for the do-not-resuscitate process. *J Med Ethics* 2010;36(9):518-520.
2. Findlay GP, Shotton H, Kelly K, *et al*. *Time to intervene? A review of patients who underwent cardiopulmonary resuscitation as a result of an in-hospital cardiorespiratory arrest.* A report by the National Confidential Enquiry into Patient Outcome and Death, 2012.
3. Fritz Z, Malyon A, Frankau JM, *et al*. The Universal Form of Treatment Options (UFTO) as an alternative to Do Not Attempt Cardiopulmonary Resuscitation (DNACPR) orders: a mixed methods evaluation of the effects on clinical practice and patient care. *PloS one* 2013;8(9):e70977.
4. Nicholson TR, Cutter W, Hotopf M. Assessing mental capacity: The mental capacity act. *BMJ* 2008;336(7639):322-325.
5. Craig P, Dieppe P, Macintyre S, *et al*. Developing and evaluating complex interventions: the new Medical Research Council guidance. *BMJ* 2008;337:a1655.
6. Council of Europe (1950) *European Convention for the Protection of Human Rights and Fundamental Freedoms, as amended by Protocols Nos. 11 and 14,* November 4 (ets, no. 5). Available at: http://www.refworld.org/docid/3ae6b3b04.html (Accessed: 08 March 2017).
7. May C, Finch T, Mair F, et al. Understanding the implementation of complex interventions in health care: the normalization process model. *BMC health services research* 2007;7(1):148.
8. May C. Agency and implementation: understanding the embedding of healthcare innovations in practice. *Social science & medicine* 2013;78:26-33.
9. Damschroder LJ, Aron DC, Keith RE, *et al*. Fostering implementation of health services research findings into practice: a consolidated framework for advancing implementation science. *Implementation science* 2009;4(1):1.
10. Moore GF, Audrey S, Barker M, *et al*. Process evaluation of complex interventions: Medical Research Council guidance. *BMJ* 2015;350:h1258.
11. May CR, Johnson M, Finch T. Implementation, context and complexity. *Implementation Science* 2016;11(1):141.
12. May C. Mobilizing modern facts: health technology assessment and the politics of evidence. *Sociology of Health & Illness* 2006;28(5):513-32.
13. Lund S, Richardson A, May C. Barriers to advance care planning at the end of life: an explanatory systematic review of implementation studies. *PLoS One* 2015;10(2):e0116629.
14. May C, Finch T. Implementing, embedding, and integrating practices: an outline of normalization process theory. *Sociology* 2009;43(3):535-554.
15. May CR, Mair F, Finch T, *et al*. Development of a theory of implementation and integration: Normalization Process Theory. *Implementation Science* 2009;4(1):1.
16. May C, Rapley T, Mair FS, *et al*. *Normalization Process Theory On-line Users' Manual, Toolkit and NoMAD instrument*. 2015. Available at: http://www.normalizationprocess.org.
17. Finch TL, Girling M, May CR, *et al*. *NoMAD: Implementation measure based on Normalization Process Theory*. [Measurement instrument]. 2015. Retrieved from http://www.normalizationprocess.org.
18. Pham MT, Rajić A, Greig JD *et al*. A scoping review of scoping reviews: advancing the approach and enhancing the consistency. *Research synthesis methods* 2014;5(4):371-385.
19. Davis K, Drey N, Gould D. What are scoping studies? A review of the nursing literature. *International journal of nursing studies* 2009;46(10):1386-1400.

For peer review only - http://bmjopen.bmj.com/site/about/guidelines.xhtml

20. Arksey H, O'Malley L. Scoping studies: towards a methodological framework. *International journal of social research methodology* 2005;8(1):19-32.
21. Levac D, Colquhoun H, O'Brien KK. Scoping studies: advancing the methodology. *Implementation Science* 2010;5(1):1.
22. Fritz Z, Cork N, Dodd A, *et al.* DNACPR decisions: challenging and changing practice in the wake of the Tracey judgment. *Clinical Medicine* 2014;14(6):571-576.
23. Perkins GD, Griffiths F, Slowther A-M, *et al.* Do-not-attempt-cardiopulmonary-resuscitation decisions: an evidence synthesis. *Health Services and Delivery Research* 2016;4(11).
24. *RESPECT: Recommended Summary Plan for Emergency Care and Treatment* (2017) Available at: www.respectprocess.org.uk (Accessed 05 July 2017).
25. Hsieh H-F, Shannon SE. Three approaches to qualitative content analysis. *Qualitative health research* 2005;15(9):1277-1288.
26. Watson R, Parr J, Joyce C, *et al*. Models of transitional care for young people with complex health needs: a scoping review. *Child Care Hlth Dev* 2011;37(6):780-791.
27. Johnson MJ, May CR. Promoting professional behaviour change in healthcare: what interventions work, and why? A theory-led overview of systematic reviews. *BMJ open* 2015;5(9):e008592.
28. Gallacher K, Morrison D, Jani B, *et al*. Uncovering treatment burden as a key concept for stroke care: a systematic review of qualitative research. *PLoS Med* 2013;10(6):e1001473.
29. Mair FS, May C, O'Donnell C, *et al*. Factors that promote or inhibit the implementation of e-health systems: an explanatory systematic review. *Bulletin of the World Health Organization* 2012;90(5):357-364.
30. May C, Sibley A, Hunt K. The nursing work of hospital-based clinical practice guideline implementation: an explanatory systematic review using Normalisation Process Theory. *International journal of nursing studies* 2014;51(2):289-299.
31. Carey I, Shouls S, Bristowe K, *et al*. Improving care for patients whose recovery is uncertain. The AMBER care bundle: design and implementation. *BMJ supportive & palliative care* 2015;5(1):12-18.
32. Etkind SN, Karno J, Edmonds PM, et al. Supporting patients with uncertain recovery: the use of the AMBER care bundle in an acute hospital. *BMJ supportive & palliative care* 2015;5(1):95-98.
33. Morris M, Briant L, Chidgey-Clark J, *et al*. Bringing in care planning conversations for patients whose recovery is uncertain: learning from the AMBER care bundle. *BMJ supportive & palliative care* 2011;1(1):72-72.
34. Nutt S, Lanaghan K. *Management of Patients who are End of Life*. Doncaster and Bassetlaw Hospitals NHS Foundation Trust, 2015.
35. Guy's and St Thomas' NHS Foundation Trust. *The AMBER care bundle – a guide for patients, their relatives and carers*. Patient information leaflet, 2012.
36. Fritz Z, Fuld JP. Development of the Universal Form Of Treatment Options (UFTO) as an alternative to Do Not Attempt Cardiopulmonary Resuscitation (DNACPR) orders: a cross-disciplinary approach. *J Eval Clin Pract* 2015;21(1):109-117.
37. *UFTO: Universal Form of Treatment Options* (2015) Available at: www.ufto.org (Accessed 01 July 2015).
38. *Universal Form of Treatment Options: Scientific Background* (2015) Available at: http://www.ufto.org/background/scientific-background/ (Accessed: 01 July 2015).
39. Brimblecombe C, Crosbie D, Lim WK, *et al*. The Goals of Patient Care project: implementing a proactive approach to patient-centred decision-making. *Intern Med J* 2014;44(10):961-966.
40. Hickman SE, Nelson CA, Moss AH, *et al*. Use of the Physician Orders for Life-Sustaining Treatment (POLST) paradigm program in the hospice setting. *J Palliat Med* 2009;12(2):133-141.

For peer review only - http://bmjopen.bmj.com/site/about/guidelines.xhtml

41. Hockley J, Watson J, Oxenham D, *et al*. The integrated implementation of two end-of-life care tools in nursing care homes in the UK: an in-depth evaluation. *Palliat Med* 2010;24(8):828-838.
42. Kaldjian LC, Broderick A. Developing a policy for do not resuscitate orders within a framework of goals of care. *Jt Comm J Qual Patient Saf* 2011;37(1):11-19.
43. Mbriwa K, Limaye S. HOW CAN WE IMPROVE 'DO NOT ATTEMPT CARDIOPULMONARY RESUSCITATION' (DNACPR) AND END OF LIFE CARE (EOLC) DECISION MAKING? *Age and Ageing* 2014:43(suppl_1): i10.
44. Newport KB, Patel S, Lyckholm L, *et al*. The "PSOST": Providers' Signout for Scope of Treatment. *J Palliat Med* 2010;13(9):1055-1058.
45. Piers RD, Benoit DD, Schrauwen WJ, *et al*. Do-not-resuscitate decisions in a large tertiary hospital: differences between wards and results of a hospital-wide intervention. *Acta Clinica Belgica* 2014;66:2,116-122.
46. Stockdale C, Trivedi B, Jerome E, *et al*. Implementation of a combined Cardiopulmonary Resuscitation and Treatment Escalation Plan document in a District General Hospital. *BMJ Quality Improvement Reports* 2013;2(2):u202653. w201236.
47. Dahill M, Powter L, Garland L, et al. Improving documentation of treatment escalation decisions in acute care. BMJ quality improvement reports 2013;2(1):u200617. w201077.
48. Powter L, Dahill M, Garland L, *et al. Improving decision making and documentation of resuscitation and ceiling of treatment decisions*. Available at: http://www.acutemedicine.org.uk/wp-content/uploads/2012/10/aqi28%20-%20improving%20decision%20making%20and%20documentation%20of%20resuscitation%20and%20ceiling%20of%20treatment%20decisions.pdf (Accessed 27 June 2016).
49. Mercer MH. The death of DNR: Treatment escalation plans. (Letter to the editor). *BMJ* 2009;338:b2020.
50. Obolensky L, Mercer M. (2007) Replacing Do-Not-Resuscitate Forms with Treatment Escalation Plans. *The Intensive Care Society Annual Spring Meeting*.
51. Obolensky L, Mercer M. (2007) Patients' Experiences of Treatment Escalation Plans. *The Intensive Care Society Annual Spring Meeting.*
52. Karakusevic C. *Devon Treatment Escalation Plan & Resuscitation Decision Record (TEP).* Available at: http://www.macmillan.org.uk/documents/aboutus/health_professionals/primarycare/showcasing/2013/treatment-escalation-and-resuscitation-devon.pdf (Accessed: 23 June 2016).
53. Gray C. *Treatment Escalation Plans (TEP) and Resuscitation Decision Records*. Torbay and Southern Devon Health and Care NHS Trust, 2014.
54. Care Quality Commission. *North Devon Healthcare NHS Trust End of Life Care Quality Report.* End of Life Care Quality Report, 2014.
55. Human S, Scott K. *Treatment Escalation Plan (TEP) Forms.* Rowcroft Hospice, 2013.
56. Devon LMC Newsletter. *Treatment Escalation Plans (TEP) – new update*. Newsletter article, August 2013.
57. Hill S, Mitchell P, Cooper J, *et al. New TEP form (Version 10) – Frequently Asked Questions*. Macmillan Cancer Support, St Luke's Hospice Plymouth, East and West Devon Clinical Commissioning Group.
58. Diamond A. *Briefing note on the use of Treatment Escalation Plans (TEPs).* North Devon Healthcare NHS Trust.
59. North Devon Healthcare NHS Trust. *Guidance for completing Treatment Escalation Plan and Resuscitation Decisions*. Available at: http://www.northdevonhealth.nhs.uk/wp-content/uploads/2015/07/V10-FINAL-Guidance-for-TEP-and-Flowchart.pdf (Accessed 21 March 2017).

For peer review only - http://bmjopen.bmj.com/site/about/guidelines.xhtml

60. Paes P, O'Neill C. Treatment escalation plans–a tool to aid end of life decision making? *BMJ Supportive & Palliative Care* 2012;2(Suppl 1):A60-A60.
61. Paes P. *Treatment Escalation Planning - Introducing systematic senior clinical decision making.* Available at: http://www.karenpicking.co.uk/images/Paul%20Paes%20-%20project %20poster.pdf (Accessed: 23 June 2016).
62. Hannah J. (2014) *One TEP at a time.... Treatment Escalation Plans in Medical High Dependency.* Available at: http://www.qihub.scot.nhs.uk/media/618301/ julie%20hannah.pdf (Accessed: 08 March 2017).
63. Thomson B, Kok K, Harrod E, *et al*. *Introducing Treatment Escalation Plans in Acute Medicine*. Available at: http://www.acutemedicine.org.uk/wp-content/uploads/2014/10/AQI-40-Introducing-Treatment-Escalation-Plans-in-Acute-Medicine-A-Quality-Improvement-Project.pdf (Accessed: 23 June 2016).
64. Over J. *Treatment Escalation Plan and Resuscitation Decision Record (in relation to the adult patient over 18 years)*. Royal Cornwall Hospitals NHS Trust, 2015.
65. West Suffolk NHS Foundation Trust Resuscitation Committee. *Escalation Plan and Resuscitation Status (EPARS) (including DNACPR).* West Suffolk NHS Foundation Trust, 2013.
66. Basildon and Thurrock University Hospitals NHS Foundation Trust Critical care. *Treatment Escalation Plan (TEP*). Basildon and Thurrock University Hospitals NHS Foundation Trust, 2014.
67. Downar J, Hawryluck L. What should we say when discussing "code status" and life support with a patient? A Delphi analysis. *J Palliat Med* 2010;13(2):185-195.
68. Bailey FA, Williams BR, Woodby LL, *et al*. Intervention to improve care at life's end in inpatient settings: the BEACON trial. *J Gen Intern Med* 2014;29(6):836-843.
69. Bookbinder M, Blank AE, Arney E, *et al*. Improving end-of-life care: development and pilot-test of a clinical pathway. *J Pain Symptom Manage* 2005;29(6):529-543.
70. Bernacki RE, Block SD; American College of Physicians High Value Care Task Force. Communication about serious illness care goals: a review and synthesis of best practices. *JAMA Intern Med* 2014;174(12):1994-2003.
71. Hoerger M, Epstein RM, Winters PC, *et al*. Values and options in cancer care (VOICE): study design and rationale for a patient-centered communication and decision-making intervention for physicians, patients with advanced cancer, and their caregivers. *BMC Cancer* 2013;13:188.
72. Markoczy L. Consensus formation during strategic change. *Strategic Management Journal* 2001;22(11):1013-1031.
73. Nugus P, Greenfield D, Travaglia J, *et al*. How and where clinicians exercise power: Interprofessional relations in health care. *Social science & medicine* 2010;71(5):898-909.
74. Richens Y, Rycroft-Malone J, Morrell C. Getting guidelines into practice: a literature review. *Nursing Standard* 2004;18(50):33-40.
75. Checkland PB. (1981) *Systems thinking, systems practice*. Chichester: John Wiley.
76. Stetler CB, Legro MW, Rycroft-Malone J, *et al*. Role of "external facilitation" in implementation of research findings: a qualitative evaluation of facilitation experiences in the Veterans Health Administration. *Implementation Science* 2006;1(1):1.
77. Locock L, Dopson S, Chambers D, *et al*. Understanding the role of opinion leaders in improving clinical effectiveness. *Social science & medicine* 2001;53(6):745-757.
78. Phillipson L, Goodenough B, Reis S, *et al*. Applying Knowledge Translation Concepts and Strategies in Dementia Care Education for Health Professionals: Recommendations From a Narrative Literature Review. *Journal of Continuing Education in the Health Professions* 2016;36(1):74-81.

For peer review only - http://bmjopen.bmj.com/site/about/guidelines.xhtml

79. Field RA, Fritz Z, Baker A, *et al*. Systematic review of interventions to improve appropriate use and outcomes associated with do-not-attempt-cardiopulmonary-resuscitation decisions. *Resuscitation* 2014;85(11):1418-1431.

80. National Institute for Health Research. *Patients and the public*. 2016. Available at: http://www.nihr.ac.uk/patients-and-public/ (Accessed 04 November 2016).

81. May C, Montori VM, Mair FS. We need minimally disruptive medicine. *BMJ* 2009;339:b2803.

82. Shippee ND, Shah ND, May CR, *et al*. Cumulative complexity: a functional, patient-centered model of patient complexity can improve research and practice. *Journal of clinical epidemiology* 2012;65(10):1041-1051.

For peer review only

For peer review only - http://bmjopen.bmj.com/site/about/guidelines.xhtml

Figure 1. Flow diagram of literature screening

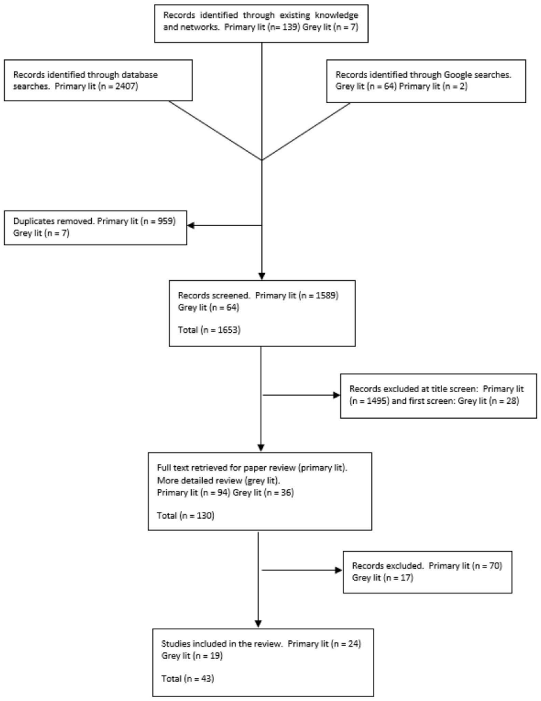

210x297mm (300 x 300 DPI)

Appendix 1: ***Primary literature search strategy***

Combined key word searches		Number of results in MEDLINE	3rd key word added if results >200
End of life care	Goals of care	(298) 12	Treatment decision making
End of life care	Ceiling of care	5	
End of life care	Ceiling of treatment	1	
End of life care	Treatment limitations	12	
End of life care	Resuscitation	(545) 11	Treatment decision making
End of life care	DNACPR	(859) 79	Goals of care
End of life care	Treatment escalation plans	0	
End of life care	Treatment decision making	68	
End of life care	Advance care plans	28	
End of life care	Supported decision making	8	
End of life care	End of life decisions	(624) 4	Treatment limitations for Medline, goals of care for CINAHL
End of life care	Treatment goals	56	
Rapid response	Goals of care	11	
Rapid response	Ceiling of care	0	
Rapid response	Ceiling of treatment	0	
Rapid response	Treatment limitations	5	
Rapid response	Resuscitation	(239) 2	Treatment limitations or End of life decisions
Rapid response	DNACPR	(549) 5	Palliative care
Rapid response	Treatment escalation plans	0	
Rapid response	Treatment decision making	4	
Rapid response	Advance care plans	0	
Rapid response	Supported decision making	0	
Rapid response	End of life decisions	1	
Rapid response	Treatment goals	5	
Medical emergency	Goals of care	53	
Medical emergency	Ceiling of care	0	
Medical emergency	Ceiling of treatment	2	
Medical emergency	Treatment limitations	8	
Medical emergency	Resuscitation	(6,041) 11	End of life decisions
Medical emergency	DNACPR	(2,291) 7	End of life decisions or Treatment goals
Medical emergency	Treatment escalation plans	0	
Medical emergency	Treatment decision making	26	
Medical emergency	Advance care plans	0	

For peer review only

Medical emergency	Supported decision making	4	
Medical emergency	End of life decisions	22	
Medical emergency	Treatment goals	17	
Critical care outreach team	Goals of care	0	
Critical care outreach team	Ceiling of care	0	
Critical care outreach team	Ceiling of treatment	0	
Critical care outreach team	Treatment limitations	0	
Critical care outreach team	Resuscitation	2	
Critical care outreach team	DNACPR	0	
Critical care outreach team	Treatment escalation plans	0	
Critical care outreach team	Treatment decision making	0	
Critical care outreach team	Advance care plans	0	
Critical care outreach team	Supported decision making	0	
Critical care outreach team	End of life decisions	0	
Critical care outreach team	Treatment goals	0	
Palliative care	Goals of care	(633) 27	End of life decisions or Treatment decision making
Palliative care	Ceiling of care	4	
Palliative care	Ceiling of treatment	0	
Palliative care	Treatment limitations	34	
Palliative care	Resuscitation	(599) 54	Goals of care
Palliative care	DNACPR	(2,232) 84	Goals of care
Palliative care	Treatment escalation plans	0	
Palliative care	Treatment decision making	146	
Palliative care	Advance care plans	12	
Palliative care	Supported decision making	3	
Palliative care	End of life decisions	(359) 28	Treatment decision making or Goals of care
Palliative care	Treatment goals	182	
Clinical deterioration	Goals of care	5	
Clinical deterioration	Ceiling of care	0	
Clinical deterioration	Ceiling of treatment	0	
Clinical deterioration	Treatment limitations	1	
Clinical deterioration	Resuscitation	67	

For peer review only

Clinical deterioration	DNACPR	220	N/A (adding 3rd term gave 0 results)
Clinical deterioration	Treatment escalation plans	0	
Clinical deterioration	Treatment decision making	3	
Clinical deterioration	Advance care plans	0	
Clinical deterioration	Supported decision making	1	
Clinical deterioration	End of life decisions	0	
Clinical deterioration	Treatment goals	7	
Treatment escalation plans	--------------------	10	
Advance care plans	--------------------	57	
Treatment decision making	Resuscitation	49	
Treatment limitations	Resuscitation	25	

For peer review only

Appendix 2: *Data extraction tool*

ARTICLE DETAILS					
Study Title					
Primary Author					
Year					
Country					
Setting	Community/Population		Nursing home/Residential care		
	Hospital/Secondary		General Practice/Primary care		
	Other (specify):				
Study design					
Participant(s)					
What is the research objective?					
Is the study describing a new/existing decision or communication tool?	Yes	Name of tool:		No	
What are the decisions of interest being studied? (e.g. DNACPR/active medical care/palliative care)	DNACPR		Other (specify):		
	Active Medical Care				
	Supportive/Palliative Care				
What are the core components of the tools/intervention being studied?	Escalation levels/interventions		Record of who plan discussed was discussed		
	Resuscitation status		Indication of person recording discussion and decision		
	Document rationale for treatment decision and resuscitation status		Mental capacity assessment		
	Review/discontinuation date		Prompt to record completion in notes (relating to separate tool only)		

For peer review only

Who makes decisions (e.g. consultant, junior doctors) and who is involved in decision-making (e.g. patients and families)?				
What is the trigger to initiating the process of decision making and/or what factors are taken into consideration? (e.g in advance of deterioration/in crisis or emergency situation)	All patients within study setting		Selected patients within study setting	
**Coherence**<br><br>*Sense making work individually and collectively when faced with the problem of operationalizing an intervention*	1) Differentiation: Does the intervention differ, and if so, how is it different from current practices? What work has been undertaken to aid understanding of how the new intervention differs from current practice?			
	2) Communal specification: Do staff have a shared understanding of the purpose of the intervention? How has a shared understanding of the purpose of the intervention been built among staff within the organisation?			
	3) Individual specification: Do staff understand their specific tasks and responsibilities in relation to the intervention? What work has been done to ensure individual tasks and responsibilities are understood. Do individuals understand how the intervention will impact upon the nature of their work?			
	4) Internalization: Do staff understand the potential value of the intervention for their work? What work has been done to promote understanding of the value, benefits and importance of the new intervention among staff and the organisation as a whole?			
**Cognitive participation**<br><br>*Relational work people do to build and sustain a community of practice around a new intervention*	1) Initiation: Are there key individuals identified as those driving the intervention forward?			
	2) Enrolment: What work has been done to promote others to ‘buy-in’ and engage with the intervention, including training and education? Are people open to working with others in new ways to adopt the intervention?			

For peer review only

	3) Legitimation: Do staff believe that participating in the intervention is a legitimate part of their role? What work has been undertaken to ensure people believe it is right for them to be involved with the intervention?
	4) Activation: Do people continue to support the intervention? What work has been undertaken to sustain the new intervention in practice and keep people supporting it's use?
**Collective action**<br><br>*The operational work that people do to enable new practices*	1) Interactional workability: Can the intervention be easily integrated into existing work practices? What work has been undertaken to allow the new intervention to integrate with existing staff and organisational practices?
	2) Relational integration: Do individuals feel confident in their own and others' abilities to use the intervention? What work has been undertaken to build knowledge and confidence in using the new intervention? Does the intervention impact upon working relationships and what work has been done to minimise this?
	3) Skill set workability: Has the work (associated with the intervention) been assigned to those with the most appropriate skills? What formed the decision behind how the division of labour was allocated? Has sufficient training given staff the skills to enable them to use the intervention?
	4) Contextual integration: What resources have been provided to support the new intervention in practice? Is the intervention adequately supported at managerial level?
**Reflective monitoring**<br><br>*The appraisal work that people do to assess and understand the ways a new intervention affects them and others around them*	1) Systemization: Has information been collected or are there plans to collect information to determine the usefulness of the intervention through feedback, audit or other means? Have the effects of the intervention been reported back to those involved?
	2) Communal appraisal: Is there communal agreement among staff as to the value of the intervention? Has the worth of the intervention been evaluated collaboratively in formal or informal groups?

For peer review only

	3) Individual appraisal: What is the effect of the intervention on an individuals' workload? Do individuals value the effect it has on their individual work?
	4) Reconfiguration: Has appraisal work lead to attempts to improve or modify the intervention?
Do patients and families understand the purpose of the intervention? What work has been done to increase their understanding?	
What is the work that patients have to do and do they involve others in the work (eg. family members)?	
Has feedback from patient/families been sought? Do they see the value of the intervention?	
What are the types of outcomes measured by the study?	
Other	

For peer review only

Appendix 3: The constructs and sub-constructs of NPT [16]

Core Constructs		Sub-constructs	
**Coherence**	The work required to make sense of: a) an intervention, both individually and collectively as a healthcare professional, clinical care team, service and/or organization. b) what is required to individually and collectively implement the intervention (in this case a tool and/or process to support decision making regarding goals of care in the context of pathophysiological deterioration)	**Differentiation**	Understanding how a new set of practices are different from current practices
		**Communal Specification**	How people work together to build a shared understanding of the aims, objectives and expected benefits of a set of new practices
		**Individual Specification**	Work that will help individuals understand their specific tasks and responsibilities around a set of practices
		**Internalization**	Work to understand the value, benefits and importance of a set of practices
**Cognitive Participation**	The relational work required within a clinical care team, service and/or organization to establish and sustain the particular intervention	**Initiation**	Work to drive the new set of practices forward
		**Enrolment**	Organizing or reorganizing of participants in order to collectively contribute to the work involved in new practices
		**Legitimation**	The work of ensuring that participants believe it is right for them to be involved and that they can make a valid contribution
		**Activation**	Actions and procedures needed to sustain a practice and maintain participant involvement
**Collective Action**	The operational work of organising and preparing the care environment, such as a	**Interactional Workability**	The interactional work that people do with each other and with other elements of a set of practices, when they seek to operationalise them in everyday settings

For peer review only

	particular service or organisation, to enact the new practices required to deliver the intervention	**Relational Integration**	The knowledge work that people do to build accountability and maintain confidence in a set of practices and in each other as they use them
		**Skill set Workability**	The allocation work that underpins the division of labour that is built up around a set of practices
		**Contextual Integration**	Managing a set of practices through the allocation of different kinds of resources and the execution of protocols, policies and procedures
**Reflexive Monitoring**	The appraisal work that people do to assess the acceptability and utility of the intervention, as well as the outcomes of the intervention and its delivery on those who negotiate and enact it (clinicians, individual patients and their families/carers)	**Systemization**	The work of collecting information to determine the effectiveness and usefulness of a new set of practices
		**Communal appraisal**	Participants working together in formal collaboratives or in informal groups to evaluate the worth of a set of practices
		**Individual appraisal**	Participants in a new set of practices work as individuals to appraise its effects on them and the contexts in which they are set
		**Reconfiguration**	Appraisal work by individuals or groups may lead to attempts to redefine procedures or modify practices

For peer review only

Appendix 4: *Summary of findings by NPT construct*

Core NPT Construct [16]	NPT Sub-Construct and Questions [16]	Key Findings	Identified Themes
Coherence	*Differentiation*<br>- Does the intervention differ, & if so, how is it different from current practices?<br>- What work has been undertaken to aid understanding of how the new intervention differs from current practice?	- High clarity & description of differentiation, & where relevant, explanation of aspects that were similar to or based upon existing practice: based upon existing national programmes of work [40, 44], frameworks [41] or guidelines [69]; established from the format of locally used tools [44], adapted from local trusts [46] or consisted of adaptations to pre-existing interventions [43, 45, 46]; entirely new interventions developed [1, 3, 31, 39, 50-52, 67], usually resulting from extensive scoping work [31, 36, 67].<br>- Interventions were developed to replace or complement existing practices or tools [1, 3, 33, 39, 41, 43, 46, 50-52].<br>- To understand how & why interventions should differ from current practices the following were drawn upon: case note reviews [46]; staff interviews [36]; scoping of published literature & guidelines [42, 67, 69]; review of local practices & contexts [31, 36, 47, 48, 61, 68].	Workability/functionality<br>Input into development
	Communal specification<br>- Do staff have a shared understanding of the purpose of the intervention?<br>- How has a shared understanding of the purpose of the intervention been built among staff within the organisation?	- Number of common themes highlighted as the key purposes of interventions: reducing unnecessary or inappropriate treatment [39, 44, 65]; enabling consideration of goals of care [31, 39, 42], guiding treatment planning & decision making [31, 39, 67, 71]; improving communication & discussions between patients & clinicians [31, 39, 40, 65, 71]; respecting patient wishes & ensuring appropriate care [31, 40, 44, 53]; improving the management of uncertainty & advanced planning [31, 65]; guiding smooth transitions of care out-of-hours & between different clinical settings [44, 46, 53, 62, 64]; improving documentation, accessibility & awareness of treatment plans [44, 46, 53, 62, 65]; providing uniformity & continuity of care [44, 53, 62, 64, 65].<br>- Background evidence & scoping work has the secondary purpose of informing staff as to the rationale & purpose of interventions [3, 31, 38, 42, 43, 46-48, 53, 67, 70].<br>- Multi-disciplinary collaboration in both development [3, 36, 46-48, 52] & education provision [42, 52, 68].<br>- Accessing alternative education programmes can act as a barrier to understanding purpose [69].<br>- Staff perception of purpose only minimally described [36, 62, 69], & some evidence of staff misunderstanding purpose [31, 39, 52].	Perceived value & appraisal<br>Input into development<br>Training & education
	Individual Specification<br>- Do staff understand their specific tasks & responsibilities in relation to the intervention? What work has been done to ensure they are understood?<br>- Do individuals understand how the intervention will impact upon the nature of their work?	- Importance of training [36, 48, 52, 68] & tailored education [36, 39] in facilitating staff understanding of tasks & responsibilities.<br>- Lack of training resulting in differing interpretations of an individuals' responsibilities for the same intervention [52].<br>- Lack of understanding of responsibilities or discomfort with proposed tasks leading to ineffective or reduced use [39-41, 54, 69].<br>- Methods of training delivery: incorporation into induction programmes [48]; simulated clinical scenarios [36]; presentations & meetings [68]; provision of material resources [36, 68].	Training & education<br>Perceived value & appraisal
	Internalization<br>- Do staff understand the potential value of the intervention for their work?<br>- What work has been done to promote understanding of the value, benefits &	- Importance of implementation staff understanding the value of an intervention [36, 39, 42, 46].<br>- Foreseen advantages included prompting consideration of more forms of treatment & reducing the negative connotations of DNACPR [36, 46].<br>- Foreseen disadvantages included potential for staff confusion, committing patients to overtreatment & potential patient distress [36].<br>- Implementation area appears important in promoting value, & more challenging in specialities less used to practicing the concepts involved in the intervention [45, 69].	Perceived value & appraisal<br>Setting & context

	importance of the new intervention among staff & the organisation as a whole?	- Staff surveys [36, 46, 62, 71] & collaborative development [36, 38, 65, 67, 69] generated consensus as to the intervention's importance, & promoted value, often based on problems encountered in current practice [46, 71].<br>- National guidance & policy were drawn upon to highlight current inadequacies in practice interventions were designed to address [3, 41, 65]. Established local policy influenced the value attributed to an intervention [41].	Input into development
Cognitive Participation	*Initiation*<br>- Are there key individuals identified as those driving the intervention forward?	- Majority of interventions piloted or implemented within limited settings or specific services [32, 44, 46, 60, 61, 63], often with input into the design from staff within these settings [44, 46, 63].<br>- Key clinical proponents [41, 54, 69] or multidisciplinary teams [68] were used to liaise & orchestrate implementation over multiple sites.<br>- Interventionist/facilitator contact important in orientating & maintaining momentum of local leadership [41, 68, 69].<br>- Input into design & development from: specialists within relevant specialties [31, 41, 46, 67] & multidisciplinary contributors, gaining collective knowledge from all potential users [31, 36, 47, 48, 62].<br>- Senior clinicians or management responsible for compliance with new interventions [54, 64, 69].	Key clinical proponents<br>Input into development<br>Setting & context
	Enrolment<br>- What work has been done to promote others to 'buy-in' & engage with the intervention, including training & education?<br>- Are people open to working with others in new ways to adopt the intervention?	- Training & education encouraged 'buy-in' [3, 31, 36, 41, 42, 48, 53, 54, 68, 69]: incorporated into established working practices [42, 48, 68, 69]; led by senior clinicians from relevant specialties [31, 41, 69]; consisted of multiple modalities [31, 41, 42, 68, 69].<br>- Timeliness of training delivery: prior to implementation [3, 36]; alongside implementation [41, 42, 53, 54, 68, 69]; repeated training [31, 69].<br>- Participation in development shown to promote 'buy-in' [36, 41, 45, 46, 48, 67]. Different techniques were adopted: anonymity of input [45, 67]; individual input in isolation, or prior to group consultation [36, 45, 46, 67]; actively involving those opposed to the intervention [36]; seeking opinions from staff experienced in the subject area targeted by the intervention [31, 36, 46, 67]; input into the intervention design [46, 47, 69] & implementation process [41, 46] from staff/specialists working within implementation settings.<br>- Difficulty in transforming clinician attitudes can act as a barrier to engagement [45].	Workability/functionality<br>Key clinical proponents<br>Training & education<br>Perceived value & appraisal<br>Input into development<br>Setting & context
	Legitimation<br>- Do staff believe that participating in the intervention is a legitimate part of their role?<br>- What work has been undertaken to ensure people believe it is right for them to be involved with the intervention?	- Legitimation was important, but its extent among clinicians varied [43, 61, 69].<br>- Facilitators of legitimation: prior use of the intervention [41]; perceived usefulness of the intervention [60, 61]; opportunities to provide input or suggest modifications to the intervention, irrespective of seniority [36, 46, 47, 67].<br>- Legality appeared as both a barrier & facilitator to legitimation [45, 46]: participation related to a sense of legal duty as opposed to behaviour change [45] & concern for potential legal ramifications were a barrier for clinicians [46].	Perceived value & appraisal<br>Input into development<br>Setting & context
	Activation<br>- Do people continue to support the intervention?<br>- What work has been undertaken to sustain the new intervention in practice & keep people supporting its use?	- Evidence of diffusion of uptake & cultural change across settings [1, 31, 36, 68, 69].<br>- Follow-up resources (including guidelines & policy), & mentorship facilitated implementation & continued support of interventions [34, 42, 47, 53, 64, 65, 68, 69].<br>- Consideration given to specialty & robustness of implementation site [41, 69].<br>- Some evidence of poor completion or failure to honour interventions [40, 52].	Setting & context<br>Key clinical proponents<br>Training & education<br>Perceived value & appraisal
Collective action	*Interactional Workability*<br>- Can the intervention be easily integrated into existing work practices?<br>- What work has been undertaken to allow the new intervention to integrate with existing staff & organisational practices?	- Ability to integrate with existing working practices recognised as important [31, 36, 68].<br>- Facilitators to integration: integration as part of, or in conjunction with, existing practices [3, 31, 38, 39, 46, 64, 68]; usefulness, pragmatism & accessibility of intervention [1, 3, 36, 44, 47-49, 60, 64, 65, 71]; involvement of users in design & piloting, allowing for amendments to facilitate utility [36, 41, 46, 47]; format of intervention based on existing design [44, 46]; transferability across healthcare settings [40, 44, 64].<br>- Barriers to integration: logistical & staffing barriers including staff turnover, management changes & staff availability to complete interventions [39-41, 44]; replication of existing work [47, 69]; clinical complexity [32, 46].	Workability/functionality<br>Input into development<br>Setting & context<br>Training & education

For peer review only

		- Interactional workability of training was an important consideration [69, 71].<br>- Electronic medical health records (EMR): for some, configuration of interventions within EMR facilitated implementation [42, 68], whilst others described poor navigation of interventions within EMR [36, 52].	
	Relational Integration<br>- Do individuals feel confident in their own & others' abilities to use the intervention?<br>- What work has been undertaken to build knowledge & confidence in using the new intervention?<br>- Does the intervention impact upon working relationships & what work has been done to minimise this?	- Circulation of interventions during development/early implementation appeared important for building confidence in their use [31, 36, 44, 52, 62] & allowed for amendments to facilitate understanding [52].<br>- Low confidence in particular aspects of interventions: capacity decisions & determining goals of care [39, 52].<br>- Reduced confidence in others: the ability of clinicians from other specialties to make decisions [36]; potential prematurity of decisions [36]; evidence of interventions not being followed due to disagreement by on-call clinicians [46].<br>- Increased confidence in others: clinicians keen to involve other clinicians & nurses expressed their confidence in others [36].<br>- Evidence of better teamwork & communication especially out-of-hours, driven by improved clarity & consistency of decisions [3, 31, 38, 44].<br>- Multi-modal format of interventions consisting of tools, prompts, algorithms & guidelines, invoke confidence in their application [1, 3, 31, 32, 36, 39, 40, 44, 48, 61-63, 67-70].<br>- Multidisciplinary educational programmes help build knowledge & confidence across disciplines [68, 69].	Workability/functionality<br>Input into development<br>Perceived value & appraisal<br>Setting & context<br>Training & education
	Skill set Workability<br>- Has the work (associated with the intervention) been assigned to those with the most appropriate skills?<br>- What formed the decision behind how the division of labour was allocated?<br>- Has sufficient training given staff the skills to enable them to use the intervention?	- Division of work based on level & nature of responsibility, among nurses, senior & junior clinicians [1, 3, 31-33, 36, 39-46, 67-71].<br>- Delegation often controlled for as the minimum level of clinicians permitted to complete or authorise was specified [1, 3, 36, 42, 46, 48, 62-65, 69].<br>- Colleagues should delegate if they do not have adequate skills [1], but concern was apparent if staff delegated to lacked the necessary experience/skills [42].<br>- Training & education was utilised [31, 39, 41, 68, 69], proposed [70, 71], or identified as deficient [40, 45], in facilitating staff development of skills, & application of skills to the processes involved in using interventions. The following were identified as important: skills training [39, 40, 42, 45, 52, 68, 69, 71]; communication skills (highlighted as difficult, important to clinicians & requiring development) [31, 33, 39, 45, 71]; other skills including: assessment & shared understanding of goals of care [1, 31, 33, 39, 45, 71]; training in application of skills to intervention [40, 52, 54, 64, 68, 69]; having someone in a position of responsibility for training execution/attendance [64, 68, 69]; different educational materials used alongside training programmes [68, 71]; value of not over-assuming existing skillset [52].<br>- Different types of training to teach relevant skills [31, 33, 68, 69]: teaching rounds [31, 68, 69] & formal group sessions [31, 69].<br>- Consideration of experience versus skill, & the interplay of external patient & organisational factors [42, 44].	Setting & context<br>Workability/functionality<br>Training & education<br>Key clinical proponents

For peer review only

	Contextual Integration<br>- What resources have been provided to support the new intervention in practice?<br>- Is the intervention adequately supported at managerial level?	- Patient & carer information leaflets are important [35, 36, 42, 51, 55, 66] & encourage shared communication [38, 64].<br>- A variety of staff guidance resources & updates used: newsletter updates, frequently asked questions documents, briefing notes, guidebooks, pocket cards, checklists, guidelines, frameworks, DVDs, prompt lists, algorithms, procedural leaflets [36, 42, 56-59, 64, 65, 68-70].<br>- A number of interventions also functioned as guidelines, with the use of flowcharts, prompts & checklists [1, 3, 31, 32, 36, 39, 40, 42, 44, 48, 61-63, 67-70], described as encouraging full & accurate completion [31, 42, 70].<br>- Joint patient & staff resources (poster & brochure) [36, 42].<br>- Guidelines incorporated in trust policy documents [36, 42, 64, 65, 68-70].<br>- Evidence from training scenarios used to shape information guidance, in order to address misconceptions evident during training [36].<br>- Guidance was distributed: alongside training sessions [71]; in staff inductions or updates [56, 64]; in local policy documents [65]; during or alongside implementation [36, 57-59, 68, 69]; as part of the intervention itself [69, 70].<br>- Websites: UFTO intervention has its own website providing access to resources, background to development, guidance for use & updates [37, 38]. One study refers to a palliative care website as a resource for implementation [69].<br>- Policy & protocol: three intervention specific policy documents [53, 64, 65]; one end of life care policy document referring to the intervention [34]; most relate to resuscitation & escalation planning [42, 47, 53, 64, 65], but also include trust induction policies [64] & EOLC policies [34].<br>- Evidence of managerial acceptance: approval from heads of department & medical directors [48, 62]; nationwide implementation [31]; incorporation into policy & protocol [34, 42, 47, 53, 64, 65].<br>- Managerial support acts as a catalyst to instigating & maintaining implementation [41, 68, 71].<br>- Levels of contact with management, & influence of management culture recognised [41, 69].	Training & education<br>Workability/functionality<br>Perceived value & appraisal<br>Key clinical proponents
Reflexive Monitoring	*Systemization*<br>- Has information been collected or are there plans to collect information to determine the usefulness of the intervention through feedback, audit or other means?<br>- Have the effects of the intervention been reported back to those involved?	- All describe or propose the collection of information relating to measures of usefulness [1, 3, 31-33, 38-43, 45-52, 54, 60-63, 68, 69].<br>- Data collection is proposed in studies where interventions have not yet been implemented [36, 44, 67, 70, 71].<br>- Different data collection methods: medical record audit [32, 39-41, 43, 46, 60, 61, 63, 68, 69], in the majority executed pre & post implementation, or in comparison with control groups [41, 43, 60, 61, 63, 68, 69]; staff surveys or interviews, often used in combination with medical record audit [3, 40, 41, 46-48, 50, 60, 61, 63, 69]; direct ward based observation [3]; patient interviews [1, 51].<br>- Different outcomes assessed: clinical impact of interventions (e.g. patient deaths) [3, 31, 32, 61]; clinical factors (e.g. illness trajectory) related to the use of interventions [32]; changes in end of life care processes, behaviours & outcomes (e.g. opioid prescriptions) [45, 46, 68, 69]; rates of completion, adherence & compliance with new interventions [31, 39-41, 43, 45, 46, 61, 69], & specifically with regard to documentation [39, 47, 48, 63]; new staff knowledge of care principles associated with the intervention [69]; attitudes toward the intervention [40, 63]; perceived effects of the intervention on practice [41, 50]; practicalities of the intervention [60, 61].<br>- Specific measures: study specific set of evaluation tools to measure feasibility [69] & validated measure to assess harms [3].<br>- Patient & family feedback outcomes are limited in scope & number [1, 3, 31, 33, 40, 47-49, 51, 62, 69, 71]: many recommend or propose the measure of patient & family experience [3, 31, 40, 47, 48, 62, 69, 71], some include measures of patient & family experience [1, 33, 49, 51]. Although limited, feedback was positive & demonstrated preferences for new interventions over previous processes.	Perceived value & appraisal<br>Training & education

For peer review only

		- Reporting the effects of the intervention to those involved: research publication [1, 3, 31-33, 38-43, 45-47, 49-51, 54, 60, 68, 69]; shared on intervention website [3, 38]; results communicated to promote shared learning [54]; shared with consultant panels for improved insight [47].	
	Communal appraisal<br>- Is there communal agreement among staff as to the value of the intervention?<br>- Has the worth of the intervention been evaluated collaboratively in formal or informal groups?	- One study evaluated staff perceptions in a collaborative setting, including focus groups & leadership meetings [69].<br>- Perceived value was evaluated via interviews, audits & questionnaires [3, 40, 41, 46-48, 50, 60, 61, 63, 69].<br>- The importance of individual & group methods in gauging attitudes was described, yet their relative efficiency was not explored [40, 41, 69].<br>- Communal staff appraisal may allow for a greater demonstration of impact than medical record audit data [69].<br>- Many demonstrated communal agreement as to the high level of value that staff attributed to an intervention [3, 40, 41, 47, 48, 50, 60-62, 69].<br>- Assessment of worth related to the intended (& perceived) purpose of interventions [31, 36, 39, 40, 42, 44, 46, 53, 62, 64, 65, 67, 69, 71].<br>- Only one study described negative staff perceptions [40].	Perceived value & appraisal
	Individual appraisal<br>- What is the effect of the intervention on an individuals' workload?<br>- Do individuals value the effect it has on their individual work?	- High value attributed to the intervention by staff, due to positive impact on working practices: reduces negative associations with DNACPR due to its universal application [3] or focus on positive treatment actions [50]; prevents unwanted resuscitation or escalation of care & ensures appropriate level of care [40, 44, 46, 47, 50]; provides a mechanism for forward planning: assessing, communicating & honouring patient preferences [3, 40]; increases staff confidence with, & improves clarity of, patient care plans [40, 44, 47, 50, 62]; improves team working & communication, especially out-of-hours [33, 46, 62]; improves ease & motivation of decision-making processes [44, 47, 60-62].<br>- Perceived & noted increases in workload were a concern for staff. However, it was generally perceived to be a worthwhile investment due to clarity, patient benefit & time saved later on [1, 3, 44, 60, 61, 69].	Workability/functionality<br>Perceived value & appraisal<br>Setting & context
	Reconfiguration<br>- Has appraisal work lead to attempts to improve or modify the intervention?	- Importance of appraisal & reconfiguration emphasised by evidence of its occurrence [31, 32, 36, 39, 41, 42, 46, 47, 51, 61, 62, 69].<br>- Reconfiguration part of the implementation process: adoption of PDSA (plan, do, study, act) cycles [46, 47]; piloting the intervention prior to roll out (with feedback, evaluation & subsequent amendments) [36]; early testing & adaptations [31]; use of sequential role out to amend implementation processes based on gathered insight [69].<br>- Appraisal & feedback led to suggested [69] & actual modifications [31, 36, 39, 46, 47, 61]: refinement of identification or trigger questions [31, 69]; ease of use, layout & clarity [36, 47]; inclusion & clarity of review dates [36, 46]; inclusion of free text boxes [36, 46].<br>- Appraisal led to further study or proposed new areas of study [32, 39, 61, 62, 69]: additional research to determine impact on specific outcomes [39]; feasibility of interventions over longer timeframes [69] & across different settings [32, 61]; studies to address distinct but associated problems identified during the appraisal work (e.g. lack of clinician presence at family meetings) [32, 62, 69].<br>- Appraisal also led to recognition of the need for, & subsequent development of, new patient information materials & services [51, 69], proposals for staff support services [41] & the revision & inclusion of interventions in local & trust policy [42, 47, 61].<br>- In two studies, appraisal demonstrated no desired impact, leading to the proposal or development of alternative interventions [43, 45].	Perceived value & appraisal<br>Input into development<br>Workability/functionality<br>Setting & context<br>Training & education

For peer review only

PRISMA 2009 Checklist

Section/topic	#	Checklist item	Reported on page #
**TITLE**			
Title	1	Identify the report as a systematic review, meta-analysis, or both.	1
**ABSTRACT**			
Structured summary	2	Provide a structured summary including, as applicable: background; objectives; data sources; study eligibility criteria, participants, and interventions; study appraisal and synthesis methods; results; limitations; conclusions and implications of key findings; systematic review registration number.	2
**INTRODUCTION**			
Rationale	3	Describe the rationale for the review in the context of what is already known.	3-4
Objectives	4	Provide an explicit statement of questions being addressed with reference to participants, interventions, comparisons, outcomes, and study design (PICOS).	4
**METHODS**			
Protocol and registration	5	Indicate if a review protocol exists, if and where it can be accessed (e.g., Web address), and, if available, provide registration information including registration number.	N/A for scoping reviews
Eligibility criteria	6	Specify study characteristics (e.g., PICOS, length of follow-up) and report characteristics (e.g., years considered, language, publication status) used as criteria for eligibility, giving rationale.	4-5
Information sources	7	Describe all information sources (e.g., databases with dates of coverage, contact with study authors to identify additional studies) in the search and date last searched.	5-6
Search	8	Present full electronic search strategy for at least one database, including any limits used, such that it could be repeated.	5 & appendix 1
Study selection	9	State the process for selecting studies (i.e., screening, eligibility, included in systematic review, and, if applicable, included in the meta-analysis).	4-6
Data collection process	10	Describe method of data extraction from reports (e.g., piloted forms, independently, in duplicate) and any processes for obtaining and confirming data from investigators.	6
Data items	11	List and define all variables for which data were sought (e.g., PICOS, funding sources) and any assumptions and simplifications made.	6 & appendix 2
Risk of bias in individual studies	12	Describe methods used for assessing risk of bias of individual studies (including specification of whether this was done at the study or outcome level), and how this information is to be used in any data synthesis.	N/A in scoping

For peer review only

PRISMA 2009 Checklist

			review
Summary measures	13	State the principal summary measures (e.g., risk ratio, difference in means).	7
Synthesis of results	14	Describe the methods of handling data and combining results of studies, if done, including measures of consistency (e.g., I^2) for each meta-analysis.	7

Section/topic	#	Checklist item	Reported on page #
Risk of bias across studies	15	Specify any assessment of risk of bias that may affect the cumulative evidence (e.g., publication bias, selective reporting within studies).	2
Additional analyses	16	Describe methods of additional analyses (e.g., sensitivity or subgroup analyses, meta-regression), if done, indicating which were pre-specified.	N/A
**RESULTS**			
Study selection	17	Give numbers of studies screened, assessed for eligibility, and included in the review, with reasons for exclusions at each stage, ideally with a flow diagram.	7 & figure 1
Study characteristics	18	For each study, present characteristics for which data were extracted (e.g., study size, PICOS, follow-up period) and provide the citations.	8-14
Risk of bias within studies	19	Present data on risk of bias of each study and, if available, any outcome level assessment (see item 12).	15-19 & appendix 4
Results of individual studies	20	For all outcomes considered (benefits or harms), present, for each study: (a) simple summary data for each intervention group (b) effect estimates and confidence intervals, ideally with a forest plot.	15-19 & appendix 4
Synthesis of results	21	Present results of each meta-analysis done, including confidence intervals and measures of consistency.	15-19 & appendix 4
Risk of bias across studies	22	Present results of any assessment of risk of bias across studies (see Item 15).	2
Additional analysis	23	Give results of additional analyses, if done (e.g., sensitivity or subgroup analyses, meta-regression [see Item 16]).	15-19 & appendix 4
**DISCUSSION**			

For peer review only

PRISMA 2009 Checklist

Summary of evidence	24	Summarize the main findings including the strength of evidence for each main outcome; consider their relevance to key groups (e.g., healthcare providers, users, and policy makers).	20-22
Limitations	25	Discuss limitations at study and outcome level (e.g., risk of bias), and at review-level (e.g., incomplete retrieval of identified research, reporting bias).	2 & 20-22
Conclusions	26	Provide a general interpretation of the results in the context of other evidence, and implications for future research.	22
**FUNDING**			
Funding	27	Describe sources of funding for the systematic review and other support (e.g., supply of data); role of funders for the systematic review.	23

From: Moher D, Liberati A, Tetzlaff J, Altman DG, The PRISMA Group (2009). Preferred Reporting Items for Systematic Reviews and Meta-Analyses: The PRISMA Statement. PLoS Med 6(6): e1000097. doi:10.1371/journal.pmed1000097

For more information, visit: www.prisma-statement.org.

For peer review only

BMJ Open

Implementing communication and decision-making interventions directed at goals of care: a theory led scoping review

Journal:	*BMJ Open*
Manuscript ID	bmjopen-2017-017056.R2
Article Type:	Research
Date Submitted by the Author:	10-Aug-2017
Complete List of Authors:	Cummings, Amanda; University of Southampton Faculty of Health Sciences,<br>Lund, Susi; University of Southampton,<br>Campling, Natasha; University of Southampton Faculty of Health Sciences<br>May, Carl; University of Southampton, Faculty of Health Sciences<br>Richardson, Alison; University of Southampton, Faculty of Health Sciences<br>Myall, Michelle; University of Southampton, Faculty of Health Sciences
**Primary Subject Heading**:	Health services research
Secondary Subject Heading:	Communication, Health policy, Palliative care, Emergency medicine
Keywords:	Goals of Care, Ceilings of Care, Treatment Escalation Plans, Decision Making, Normalisation Process Theory, Implementation Science

SCHOLARONE™
Manuscripts

For peer review only

Implementing communication and decision-making interventions directed at goals of care: a theory led scoping review

Amanda Cummings[1,2], Susi Lund[1,2], Natasha Campling[1,2], Carl May[1,2,3], Alison Richardson[1,2,3], Michelle Myall[1,2]

1. Faculty of Health Sciences, University of Southampton, UK
2. NIHR CLAHRC Wessex, University of Southampton, UK
3. University Hospital Southampton NHS Foundation Trust, Southampton, UK

Corresponding author: Dr Amanda Cummings, Faculty of Health Sciences, University of Southampton, Building 67, University Road, Highfield, Southampton, SO17 1BJ
Tel: +44 (0)23 8059 8250; Fax +44 (0)23 8059 8308; Email: ac2a13@soton.ac.uk

Key words: goals of care; ceilings of care; treatment escalation plans; decision-making; implementation science; Normalisation Process Theory; scoping review

Word count: 4,667 (excluding tables and references)

For peer review only

ABSTRACT

Objectives: To identify the factors that promote and inhibit the implementation of interventions which improve communication and decision-making directed at goals of care in the event of acute clinical deterioration.

Design and methods: A scoping review was undertaken based on the methodological framework of Arksey and O'Malley for conducting this type of review. Searches were carried out in MEDLINE and CINAHL to identify peer reviewed papers and in Google to identify grey literature. Searches were limited to those published in the English language from 2000 onwards. Inclusion and exclusion criteria were applied, and only papers that had a specific focus on implementation in practice were selected. Data extracted were treated as qualitative and subjected to directed content analysis. A theory informed coding framework using Normalisation Process Theory (NPT) was applied to characterise and explain implementation processes.

Results: Searches identified 2,619 citations, 43 of which met the inclusion criteria. Analysis generated six themes fundamental to successful implementation of goals of care interventions: (1) input into development; (2) key clinical proponents; (3) training and education; (4) intervention workability and functionality; (5) setting and context; and (6) perceived value and appraisal.

Conclusions: A broad and diverse literature focusing on implementation of goals of care interventions was identified. Our review recognised these interventions as both complex and contentious in nature making their incorporation into routine clinical practice dependent on a number of factors. Implementing such interventions present challenges at individual, organisational and systems levels, which make them difficult to introduce and embed. We have identified a series of factors that influence successful implementation and our analysis has distilled key learning points, conceptualised as a set of propositions, we consider relevant to implementing other complex and contentious interventions.

Strengths and limitations of this study

- This paper outlines a scoping review of a broad and diverse literature, both published and grey, focusing on the implementation of goals of care interventions for patients facing clinical deterioration. However, because of its focus on implementation, not all examples of goals of care interventions could be included.
- Normalization Process Theory (NPT) was used to investigate and explain the successful implementation of interventions, and a theoretical approach has been applied to all stages of the review process.
- In a number of included papers information relating to implementation barriers was missing and there was bias towards the presentation of positive outcomes. This may reflect a reluctance to focus on challenges with study authors keen to exhibit and promote the benefits of interventions.
- The review lead to goals of care interventions being defined as 'contentious' with a moral purpose and value, and identified the elements and learnings that could be transferable to other examples of such interventions.
- We have proposed that contentious interventions consist of components at three levels. Across the literature reviewed the focus was on components at the individual level where negotiated decision-making between participants occurs. There was limited focus on the components which take place within and across organisations and the influence of system constraints. This has important limitations for our interpretation of data and analysis.

1. INTRODUCTION

For peer review only - http://bmjopen.bmj.com/site/about/guidelines.xhtml

In the event of a patient becoming acutely unwell, treatment and care decisions are recommended by clinicians. While these decisions are based on the clinical judgement of a healthcare professional they should also be bound by the preferences and wishes of the patient and their family. Processes and tools (referred to here as goals of care interventions), that provide a framework for discussing and documenting appropriate treatment options in the event of acute clinical deterioration, are paramount. These interventions aim to improve patient and family involvement, enabling exploration and understanding of the current clinical situation and facilitation of communication and negotiated decision-making about future treatment options [1, 2]. They offer a means for patients' preferences to be taken into account, improving communication and clarity across the wider clinical team.

We refer to goals of care in the event of acute deterioration, where different levels of treatment might be appropriate and range from full escalation in a critical care environment to symptom control measures [3]. Goals of care are currently referred to using various terms, including but not confined to ceilings of care, treatment escalation plans and treatment limitations. They exist in numerous formations including a specific, dedicated paper form, a narrative entry in a paper medical record and inclusion in an electronic patient record, and may be introduced as an extension of the 'do not attempt cardio-pulmonary resuscitation' (DNACPR) process. These interventions offer a system for recording recommended treatment and care, ideally including all components of the decision pathway and can be applied across different care settings. They require a process to be created and implemented that is recognised across organisations, takes account of socio-legal frameworks, such as the UK Mental Capacity Act (MCA) (2005) [4], and is designed to protect individuals who do not have capacity to make decisions about their care and treatment.

Goals of care interventions are complex and consist of multiple interacting components. The number and difficulty of behaviours required by those delivering or receiving such an intervention, variability of outcomes, and degree of flexibility and tailoring that is permitted, all contribute to making this a complex intervention [5]. As well as being complex, goals of care interventions are established as interactions and recording systems with a moral purpose and value. This means they may be *contentious* in practice because they contain elements which seek to routinise highly complex clinical skills, practice and different types of wisdom, in a context of uncertainty. Contentiousness can arise because the intervention relies on patient, family and clinician interactions, inter-clinician interactions (potentially across clinical settings and organisational boundaries), and societal and legal frameworks, such as the MCA (2005) and the European Convention on Human Rights (ECHR) (2002) [4, 6].

In spite of a growing body of literature describing the introduction and benefits of goals of care interventions, little is known about the factors which influence their successful implementation in clinical practice. Here implementation is defined as 'any deliberately initiated attempt to introduce new, or modify existing, patterns of action in health care or some other formal organizational setting. Deliberate initiation means that an intervention is: institutionally sanctioned; formally defined; consciously planned; and intended to lead to a changed outcome' [7]. As we have previously argued, "this is more than the adoption or diffusion of innovations" as effective implementation is about interventions being made workable and embedded in routine clinical practice [7].

For peer review only - http://bmjopen.bmj.com/site/about/guidelines.xhtml

Understanding and evaluating the implementation of complex interventions in practice remains a challenge for healthcare managers, policy makers and for those who enact them outside of formal research settings [8]. Furthermore, as interventions found to be effective in the context of health services research studies can fail to translate into meaningful healthcare outcomes across varying contexts [9] this makes understanding the reasons for failure or partial success even more essential. Implementation science, which promotes the integration of research findings and evidence into healthcare policy and practice, is increasingly recognised within health services research to make a key contribution to such knowledge. Comprehensive process evaluation of the implementation of healthcare interventions is increasingly important for future learning [10], as it enables understanding of transition from closed systems of highly structured research or service development projects into the real world of open systems healthcare delivery where they are operationalised [11, 12]. Learning from the available existing knowledge in this area can be used to inform healthcare practice change and contribute to the field of implementation science.

Having previously applied Normalisation Process Theory (NPT) to aid learning in the comparable context of advance care plans [13], we have used NPT to characterise and explain implementation processes [14]. NPT provides a set of tools to investigate and understand the processes through which interventions are operationalised in healthcare settings and incorporated into everyday practice [14-17].

In this paper, we present a scoping review of goals of care interventions which aims to: *identify the factors that promote and inhibit the implementation of interventions which improve communication and decision-making directed at goals of care.* Using goals of care interventions as an example, a secondary aim of this review is to: *characterise the components and consider the implications for implementation of, contentious interventions*.

2. DESIGN AND METHODS

A scoping review was the most appropriate methodology, given the need to extract and map principles from a diverse and broad body of evidence [18, 19].

We used Arksey and O'Malley's five stage framework for conducting scoping reviews which includes: identifying the research question, identifying relevant literature, selection, charting the data and collating, summarising and reporting the results [20]. This guided the scoping review and where necessary we developed more specific procedures to inform the review process. Levac et al's recommendations for refining the methodological application were also incorporated to increase rigour of the review process [21].

Inclusion and exclusion criteria

Literature was selected using specific inclusion and exclusion criteria (Table 1), and was included if it was considered to have a specific focus on implementation in practice. To identify all relevant literature on goals of care, grey literature was included and actively sought as part of the search strategy. Only papers published from the year 2000 onwards were included as those published earlier were unlikely to reflect current practice. Papers focusing solely on DNACPR orders were excluded due to their focus on only one

For peer review only - http://bmjopen.bmj.com/site/about/guidelines.xhtml

decisional element of goals of care. Existing evidence also suggests that DNACPR decisions are not always discussed with patients or families [22]. The combining of DNACPR decisions within wider goals of care interventions has been shown to improve clarity and communication, and the focus of this review is on implementing a process whereby goals of care are discussed [23]

Table 1. Inclusion and exclusion eligibility criteria

Inclusion criteria	Exclusion criteria
Literature describing implementation of interventions related to communication and decision-making around goals of care.<br>Studies with adult patients in hospital and community settings.<br>Studies involving end of life care, clinical deterioration and clinically uncertain outcomes.<br>Studies published in the English language.<br>Papers published between 2000-2015.<br>Qualitative and quantitative studies, including clinical trials and RCTs.<br>Published conference abstracts/conference related papers<br>Grey literature (limited to: policies, reports, research posters, patient/staff guidance, websites).	Papers not describing an intervention (process or tool).<br>Papers reporting treatment effectiveness.<br>Papers describing DNACPR orders only.<br>Studies in neonatal and paediatric settings.<br>Studies involving brain stem death.<br>Studies using biomedical data and drug trials.<br>Non-English language studies<br>Papers published before 1 January 2000.

For peer review only

Search strategy and information sources

The search strategy was designed to identify primary studies and other literature, both published and unpublished, which met the eligibility criteria. Separate searches were undertaken for primary and grey literature.

Primary literature searches

The primary literature search was carried out in two stages. The first stage involved an initial search of the bibliographic database MEDLINE using a preliminary key word search based on the terms of the topic, the text terms used in titles and abstracts, and index terms used to describe articles. Full details of the primary literature search strategy are outlined in appendix 1.

Terms identified in the titles and abstracts of relevant articles produced from stage 1 were used to develop further key words for the second stage of the literature search (see appendix 1). Dual combined key word searches were conducted in MEDLINE and CINAHL bibliographic databases, with a third key word added if searches produced >200 results. In addition, further literature were identified through existing knowledge and networks.

Grey literature searches

For peer review only - http://bmjopen.bmj.com/site/about/guidelines.xhtml

Google searches were conducted using terminology relating to known goals of care interventions identified in an earlier scoping exercise which mapped the use of forms for recording these decisions in the UK. The first 10 results were screened for relevance and further review. A further Google search focused on policies and guidance related to goals of care was undertaken and the first 50 results screened for relevance.

All screened, de-duplicated citations were imported into the bibliographic software management package Endnote. Searches were completed by August 2015. This was due to the imminence of a national programme of work which has led to the Recommended Summary Plan for Emergency Care and Treatment (RESPECT)[24]. Communication and decision-making around goals of care is a growing area of interest, and it is hoped that findings from this review can be used to inform the implementation of such a major advancement in the field.

For peer review only

Screening

An extensive screening process was undertaken. At the first stage of screening, articles were assessed by two independent reviewers (AC, CRM) based on the information provided in the title. Primary literature judged to be relevant after first screen, and which met the eligibility criteria, were obtained in full text. Articles for which there was disagreement between reviewers, were also obtained in full text. Full text articles were examined for adherence to the inclusion criteria and then screened (by AC, with input from CRM and MM, in cases of uncertainty or disagreement). Grey literature were title screened for relevance and a further full review examined adherence to inclusion criteria.

Quality assessment

This scoping review included a non-heterogeneous sample of primary and grey literature, which made it difficult to universally apply quality assessment criteria. As standard to most scoping reviews, we did not undertake formal quality assessment and excluded papers only on grounds of relevance. As a result the analytical focus of this review centred on a critique of relevance and contribution of the included literature and did not consider methodological quality.

Data extraction

In line with Arksey and O'Malley's method [20], data extraction (charting) was multi-staged. In the first stage we collected descriptive characteristics from each paper such as study design and setting. In the second stage, findings and discussion sections of included literature were extracted into a data extraction tool (see appendix 2) informed by NPT [15-17].

The data extraction tool was designed to chart specific details of the literature and to understand factors influencing implementation. The tool was piloted on a sample (n=5) of primary and grey literature, with subsequent amendments resulting in the final version. Data was extracted by independent reviewers (AC, SL, MB). Following Levac et al's recommendations, two reviewers (AC, MM) independently extracted data for a 30% sample of primary literature to ensure approaches to extraction were consistent with each other and with the research aims [21].

For peer review only - http://bmjopen.bmj.com/site/about/guidelines.xhtml

Data analysis

A two-stage analytical process was undertaken. During the first stage, data extracted were treated as qualitative data and analysed using directed content analysis [25]. A theory informed coding framework was developed using the four main constructs and sub-constructs of NPT [15, 16]. Data were identified and categorised to the constructs and sub-constructs of NPT, exploring barriers and facilitators to implementation.

The use of NPT as a theoretical framework followed its successful application in a number of different healthcare intervention reviews [13, 26-30]. NPT investigates and explains the successful operationalization of interventions: how they become part of everyday practice in healthcare settings. It embodies the different types of 'work' undertaken by individuals around implementing, embedding and integrating, and allows us to understand the social structures and contexts through which new interventions are operationalised [14-17]. In relation to implementation of goals of care interventions, definitions of the four core constructs and sub-constructs of NPT used in this review are outlined in appendix 3.

During the second stage, thematic analysis of the literature content was undertaken to identify, characterise and explain the factors that shape implementation of interventions that initiate communication and decision-making around goals of care. A final higher level of analytical interpretation followed, to characterise the components of contentious interventions, and generate transferable learning outcomes for their implementation.

3. RESULTS

Searches identified 2619 citations. Following de-duplication and relevance screening, 43 sources of literature (relating to 23 interventions), including 24 items of published literature (e.g. peer-reviewed papers, conference abstracts) and 19 items of grey literature (e.g. conference posters, patient information documents), met the inclusion criteria and were included for data extraction (see figure 1). Table 2 provides a summary of the characteristics of included literature, the range of interventions described and the decisions of interest addressed, including DNACPR, goals of active care, supportive or palliative care and those which are limited to communication guidelines only.

Insert Figure 1. Here

For peer review only - http://bmjopen.bmj.com/site/about/guidelines.xhtml

For peer review only

Table 2. Summary of included literature

Name and description of intervention	Decisions of interest				Author & Year	Country & Healthcare Setting	Literature Type	Study Design & Methods	Study Objective
	DNACPR	Ceilings of active care	Supportive/Palliative Care	Communication guidelines around goals of care					
AMBER care bundle<br><br>Care bundle consisting of a proforma and sticker.<br>Promotes consistent communication and care planning for patients identified as facing uncertain recovery.	X	X			Carey et al 2014[31]	UK Hospital (5 acute wards)	*Published peer-reviewed study*	Descriptive/Quantitative: Overview of development and implementation of AMBER, and data on clinical impact.	To describe the design, development and implementation of the AMBER care bundle and its impact on clinical service.
					Etkind et al 2014[32]	UK Hospital (5 acute wards)	*Published peer-reviewed study*	Quantitative: Retrospective electronic case note review of patients who died in wards where AMBER was implemented.	To evaluate application of the AMBER care bundle in a UK hospital and describe factors affecting its use.
					Morris et al 2011[33]	UK Hospital	*Published conference abstract*	Descriptive: Overview of AMBER and its implementation.	Learning points from implementation of the AMBER care bundle.
					Doncaster and Bassetlaw Hospitals NHS Foundation Trust 2015[34]	UK NHS Trust	*Grey literature*	Policy Document	Management of patients who are at end of life.
					Guy's and St Thomas' NHS Foundation Trust 2012[35]	UK NHS Trust	*Grey literature*	Patient Guidance	The AMBER care bundle – a guide for patients, their relatives and carers.
Universal Form Of Treatment Options (UFTO)	X	X	X		Fritz et al 2013[3]	UK Hospital	*Published peer-reviewed*	Prospective mixed method:	To determine whether the introduction UFTO reduces harms in

For peer review only - http://bmjopen.bmj.com/site/about/guidelines.xhtml

Form contextualising the CPR decision within overall treatment plans.<br>Includes documentation and discussion of care and treatment decisions.						(2 acute wards)	*study*	Case note review of DNACPR patients, qualitative interviews and observations .	patients in whom a DNACPR was made, and to understand the mechanisms for observed change.
					Fritz et al 2015[36]	UK Hospital (2 acute wards)	*Published peer-reviewed study*	Expert Consensus: Cross-disciplinary adapted Delphi method.	To describe a cross-disciplinary approach to developing a universal form of treatment options as an alternative to DNACPR orders.
					www.ufto.org Accessed: 01/07/15[37]	UK	*Grey literature*	Website	UFTO: Universal form of treatment options
					www.ufto.org/background/scientific-background Accessed 01/07/15[38]	UK	*Grey literature*	Website	UFTO: Scientific background
Goals of Patient Care Summary (GOPC)<br><br>Form to summarise goals of care. Serves as a guide for treatment decisions as the patient's condition improves, stabilises, or deteriorates.	X	X	X		Brimblecombe et al 2014[39]	Australia Hospital (adult medical inpatients)	*Published peer-reviewed study*	Quantitative: Cross-sectional review of medical inpatients and audit of inpatients requiring emergency medical review.	To review the implementation of the GOPC: assess GOPC uptake, quantify completion and review outcomes of the GOPC assessment.
Physician Orders for Life-Sustaining Treatment (POLST)<br><br>Form to ensure that patient wishes regarding life-sustaining treatments are honoured.<br>Converts treatment preferences into medical orders.	X	X	X		Hickman et al 2009[40]	USA Hospice (hospices in 3 US states)	*Published peer-reviewed study*	Descriptive/Quantitative: Survey of hospice staff members. Case note review of deceased patients at subsample of POLST-using hospices.	To evaluate use of POLST by hospice programs, attitudes of hospice staff toward POLST, the effect of POLST on the use of life-sustaining treatments, and the treatment options selected by patients.
Gold Standards Framework for Care Homes (GSFCH) and an adapted Liverpool Care Pathway for Care Homes (LCP)<br><br>The implementation of two end-of-life care tools: the GSFCH supportive/palliative care register	X	X	X		Hockley et al 2010[41]	UK Nursing Home (7 nursing homes in Scotland)	*Published peer-reviewed study*	Quantitative/In-depth Evaluation: Overview of implementation, retrospective case note review of deceased residents, and staff audit.	To evaluate the effectiveness of a high facilitation implementation on professional practices and residents outcomes.

For peer review only

For peer review only - http://bmjopen.bmj.com/site/about/guidelines.xhtml

and an adapted Liverpool Care Pathway for Care Homes.									
UIHC (University of Iowa Hospital and Clinics) Policy Regarding Do Not Resuscitate Orders and End of Life Discussions with Hospitalised Patients<br><br>DNR order policy placing CPR discussions within a framework of end of life discussions, and within a larger goal orientated context.	X	X	X		Kaldjian et al 2011[42]	USA Hospital (academic medical centre)	*Published peer-reviewed study*	Descriptive: Overview of the integration of goals of care into staff education and revision of hospital DNR policy. Literature review and survey of hospitalised adults.	To describe the background and rationale for DNR policy revision and development. To describe the policy itself and ongoing implementation efforts.
A revised DNACPR form, decision making tools and updated CPR guidelines	X	X	X		Mbriwa et al 2014[43]	UK Hospital (elderly and acute medical inpatients)	*Published conference abstract*	Quantitative: Prospective case note audit.	To assess improvement in CPR decision making and ceiling of care documentation/discussions since the introduction of a revised DNACPR form, decision making tools and guidelines.
Providers Signout for Scope of Treatment (PSOST)<br><br>A daily handover tool documenting resuscitation status, goals of care, desired and undesired interventions, in addition to standard handover information.	X	X	X		Newport et al 2010[44]	USA Palliative Care (inpatient unit)	*Published peer-reviewed study*	Descriptive: Overview of tool development, implementation and utility.	To describe the PSOST tool and evaluate its implementation and use in a palliative care unit.
An intervention informing clinicians of the laws on Patients' Rights and the importance of advanced care planning, alongside a new DNR form<br><br>Adapted DNR form, putting emphasis on the motivation, participants involved and communication of the decision-making process.	X	X			Piers et al 2011[45]	Belgium Hospital (adult inpatients)	*Published peer-reviewed study*	Quantitative: Prospective observational study of deceased inpatients pre and post-intervention. Cross-sectional study of patients admitted to non-intensive wards.	To compare the quality of end-of-life care and DNR decisions between hospital wards and after a hospital-wide intervention to improve advanced care planning.
Treatment Escalation Plan and Resuscitation Decision (TEP-CPR)<br><br>Treatment escalation plan tool, to be	X	X	X		Stockdale et al 2013[46]	UK Hospital (2 acute wards)	*Published peer-reviewed study*	Quantitative/Descriptive: Case note audit and staff survey, followed by overview of TEP-CPR	To develop and test a tool for escalation planning. To identify whether a TEP-CPR could reduce the number of patients receiving

For peer review only

For peer review only - http://bmjopen.bmj.com/site/about/guidelines.xhtml

included in the resuscitation form.								development. Staff survey of OOH treatment in patients with TEP-CPR and controls.	inappropriate treatments out of hours.
Ceiling of Treatment (COT) Form<br><br>Tool for improving decision making and documentation of treatment escalation decisions	X	X	X		Dahill et al 2013[47]	UK Hospital (elderly care wards)	*Published peer-reviewed study*	Quantitative/Descriptive: Overview of COT development and implementation. Pre and post-implementation case note review of treatment escalation decisions and staff feedback survey.	To develop, pilot and implement a tool to improve decision-making and the documentation of treatment escalation decisions.
					Powter et al 2012[48]	UK Hospital (elderly care wards)	*Grey literature*	Poster<br><br>Quantitative/Descriptive: Overview of COT development and implementation. Pre and post-pilot data on decision documentation and staff survey.	To improve decision making and documentation of CPR and ceiling of treatment decisions.
Treatment Escalation Plan (Devon)<br><br>Form outlining what treatment options would be appropriate if a patient were to become acutely unwell.	X	X			Obolensky et al 2010[1]	UK Hospital (2 acute T&O wards)	*Published peer-reviewed study*	Prospective quantitative and qualitative evaluation: Patient/relative interviews to evaluate the TEP process.	To evaluate patient and relative experiences of, and thoughts regarding, the Devon TEP.
					Mercer, M 2009[49]	UK Hospital	*Published letter*	Letter to the editor<br><br>Descriptive: Overview of TEP process.	The death of DNR: Treatment escalation plans.
					Obolensky et al 2007[50]	UK Hospital	*Published Conference abstract*	Descriptive/Quantitative: Overview of TEP process and staff survey.	To evaluate initial impact of the Devon TEP from a clinical staff perspective.
					Obolensky et al 2007[51]	UK Hospital	*Published Conference abstract*	Descriptive/Quantitative: Overview of TEP process and patient survey.	To evaluate patient experiences of the Devon TEP.
					Karakusevic, C [52]	UK Hospital (acute	*Grey literature*	Poster<br><br>Descriptive:	To evaluate implementation of the Devon TEP.

For peer review only

						hospital)		Overview of background to development and TEP implementation.	
					Torbay and Southern Devon Health and Care NHS Trust 2014[53]	UK NHS Trust	*Grey literature*	Policy Document	Treatment escalation plans (TEP) and resuscitation decision records policy.
					Care Quality Commission (CQC) 2014[54]	UK NHS Trust	*Grey literature*	CQC Report	Northern Devon healthcare NHS trust, end of life care quality report.
					Rowcroft Hospice 2013[55]	UK Hospice	*Grey literature*	Patient Guidance	Treatment escalation plan (TEP) forms, public information document.
					Devon Local Medical Committee 2013[56]	UK	*Grey literature*	Staff Guidance	Treatment escalation plans (TEP) – newsletter update article.
					Macmillan Cancer Support, St Luke's Hospice Plymouth & North, East and West Devon CCG[57]	UK Hospice	*Grey literature*	Staff Guidance	New TEP form (version 10) – Frequently asked questions document.
					North Devon Healthcare NHS Trust[58]	UK NHS Trust	*Grey literature*	Staff Guidance	Staff briefing note on the use of treatment escalation plans (TEPs).
					NHS Improving Quality Team 2014[59]	UK	*Grey literature*	Staff Guidance	Guidance for completing treatment escalation plan and resuscitation decisions.
Treatment Escalation Plan (TEP) <br>Tool for planning the care of a patient at risk of deterioration.	X	X	X		Paes, P et al 2012[60]	UK Hospital (6 acute specialties)	*Published conference abstract*	Descriptive/Quantitative: Pilot, audit and staff feedback survey.	To evaluate staff feedback and decision-making outcomes before and after TEP introduction.
					Paes, P 2012[61]	UK Hospital (6 acute wards & communit	*Grey literature*	Poster <br>Descriptive/Quantitative: Overview of TEP implementation and next	To pilot TEP and evaluate staff feedback, OOH decision-making/discussions and TEP completion outcomes. To determine whether and how, TEP

						y hospital)		steps. Case note review audit before and after TEP pilot, and staff survey.	should be introduced across the trust.
HDU Specific Treatment Escalation Plan<br><br>Plan for ceiling of treatment and escalation level decision on every patient within 24hrs.	X	X			Hannah, J 2014[62]	UK Hospital (medical HDU)	*Grey literature*	Poster<br><br>Descriptive: Overview of TEP development, implementation and next steps, including informal staff feedback.	To describe and evaluate the process of TEP development and its implementation in a medical high dependency unit.
Treatment Escalation Plan Proforma	X	X	X		Thomson et al 2014[63]	UK Hospital (acute medicine)	*Grey literature*	Poster<br><br>Quantitative: Case note review audit before and after TEP pilot, and staff survey.	To pilot TEP and evaluate staff feedback and DNACPR/TEP documentation outcomes.
Cornwall Treatment Escalation Plan and Resuscitation Decision Record	*X*	*X*			Royal Cornwall Hospitals NHS Trust 2015[64]	UK NHS Trust	*Grey literature*	Policy Document	Treatment escalation plan & resuscitation decision record policy.
Escalation plan and resuscitation status (EPARS)<br><br>Form amalgamating escalation plan and CPR decisions.	X	X	X		West Suffolk NHS Foundation Trust 2013[65]	UK NHS Trust	*Grey literature*	Policy Document	Trust policy and procedure: Escalation plan and resuscitation status (EPARS) (including DNACPR).
Treatment Escalation Plan (TEP)	X	X			Basildon and Thurrock University Hospitals NHS Foundation Trust 2014[66]	UK NHS Trust	*Grey literature*	Patient Guidance	Treatment escalation plan (TEP) patient information.
Content guidelines for discussions of CPR and life-sustaining therapy<br><br>Guidelines for the content of a discussion about resuscitation or goals of care.	X	X		X	Downar et al 2010[67]	Canada Hospital (12 physicians)	*Published peer-reviewed study*	Expert Consensus: Delphi method to develop content guidelines	To develop content guidelines for discussions of CPR and life-sustaining therapy.
A palliative care treatment plan intervention			X		Bailey et al 2014[68]	USA Hospital	*Published peer-reviewed*	Descriptive/Quantitative: Overview of intervention	To evaluate the effectiveness of a multi-modal intervention strategy

For peer review only

For peer review only - http://bmjopen.bmj.com/site/about/guidelines.xhtml

Best practice guidance supported by a staff education programme, electronic decision support tool and paper educational tools.						(inpatient units at 6 veteran medical centres)	*study*	and its multi-centre implementation. Pre and post-implementation case note review of EOL care in deceased patients.	to improve the processes and quality of end-of-life care in acute inpatient settings.
The Palliative Care for Advanced Disease (PCAD) pathway<br><br>To improve palliative care for inpatients who are expected to die from advanced disease. Clinical pathway including:<br>- Inter-disciplinary care pathway<br>- Nurses documentation flowsheet<br>- Physicians order sheet with guidelines for symptom management			X		Bookbinder et al 2005[69]	USA Hospital (3 inpatient units)	*Published peer-reviewed study*	Descriptive/Quantitative: Overview of intervention, its development and implementation. Case note review of care outcomes in patients who died before and during implementation, in both intervention and control units.	To describe the development and quality improvement strategy for the implementation of PCAD. To pilot and evaluate the utility of PCAD.
Serious illness conversation guide<br><br>Guide describing key elements to be addressed in patient conversations about serious illness care goals.			X	X	Bernacki et al 2014[70]	USA	*Published peer-reviewed study*	Literature review and synthesis of best practices in conversations about serious illness care goals.	To provide clinicians with practical, evidence-based advice (in the form of a serious illness conversation guide).
The VOICE (Values and Options in Cancer Care) intervention<br><br>Education and coaching communication intervention.<br>To facilitate communication and decision making among oncologists, patients and caregivers.				X	Hoerger et al 2013[71]	USA Oncology Services	*Published peer-reviewed study*	Descriptive: Overview of intervention and its planned implementation as part of an RCT.	To describe the study design and rationale for a patient-centred communication and decision-making intervention for physicians, patients with advanced cancer, and their caregivers

For peer review only

For peer review only - http://bmjopen.bmj.com/site/about/guidelines.xhtml

Thematic analysis of literature content suggested there were six common themes fundamental to the successful implementation of goals of care interventions: input into development, key clinical proponents, training and education, intervention workability and functionality, setting and context, perceived value and appraisal. These are outlined below. The links between the sub-constructs of NPT and these identified themes are outlined in table 3. A summary of results by NPT construct are presented in appendix 4 as a summary and appraisal of the literature.

Input into development

The involvement of clinical staff in development of interventions facilitated the identification of current shortfalls in practice, understanding purpose, and shaping novelty, accessibility and utility of design [36, 41, 46, 47, 71]. Developmental input also assisted in promoting 'buy-in', legitimization and confidence in the intervention [31, 36, 41, 44-48, 62, 67]. While some literature focused on the importance of input from senior, specialist individuals [31, 41, 46, 67], including those within implementation settings [46, 47, 69], for intervention design, multi-disciplinary collaboration in the development process was common across papers [3, 31, 36, 38, 46-48, 52, 62, 65, 67, 69], and helpful for gaining collective knowledge from all potential users [31, 36, 47, 48, 62]. In one paper, individuals who had expressed disagreement with one intervention were actively sought for involvement at each stage of development [36], highlighting how inclusion in development can contribute to overcoming barriers of individual resistance. Reconfiguration of forms and procedures were incorporated in the development and implementation process in a number of initiatives [31, 36, 46, 47, 69]. The benefit of this approach was that it allowed for assessment of factors such as usability, clarity and safety, incorporating feedback into further iterations [36, 46, 69].

Key clinical proponents

Key clinical proponents were assigned to orchestrate and lead implementation within specific sites or settings [41, 54, 68, 69]. They were key resources for attending and cascading training as well as overseeing changes, embedding cultural change and providing mentorship [33, 41, 54, 68, 69]. Their direct contact with intervention instigators appeared important in orientating and maintaining momentum of local leadership, including training and facilitation [41, 68, 69]. Similarly, support from individual senior managers in ensuring compliance with training and interventions, promoting sustained use, as well as acting as catalysts for initial implementation was valuable [41, 54, 64, 68, 69, 71]. Trust level managerial support was also evident through incorporation into policy and protocol [34, 42, 47, 53, 64, 65] and approval from directors [48, 62]. Managerial backing could be influential for organisational change, although in reality, engagement with the management of healthcare organizations was recognised by some for its complexity [41, 69].

Training and education

Training and education encouraged 'buy-in' [3, 31, 36, 41, 42, 48, 53, 54, 68, 69], promoted understanding of tasks and responsibilities [31, 36, 42, 48, 68, 69] and facilitated a shared understanding of purpose [42, 52, 68]. Staff often negatively perceived the increased training requirement [36], inclusion within established working practices [42, 48, 68, 69] and feasibility of incorporation into workload [69, 71] were important considerations. Repeated sessions were used to train rotational/part-time staff, address staff turnover and enable sustained engagement [31, 47, 48, 69]. Tailored education based on evidence and

For peer review only - http://bmjopen.bmj.com/site/about/guidelines.xhtml

feedback facilitated the most efficacious training, especially since misunderstanding of responsibilities could result in ineffective or reduced use [36, 39, 41, 54, 69]. Training in the application of skills to the intervention was fundamental [64, 68, 69] and without this, regardless of skill level, the completion, interpretation and application of interventions in practice could be inconsistent [40, 52, 54]. Addressing communication skills was highlighted as important in a number of papers [31, 33, 39, 45, 71]. Individuals responsible for training could ensure that as far as possible all staff received necessary training [64, 68, 69]. The provision of guides, algorithms and interactive materials facilitated skill development [68, 71], and patient and carer information materials helped to sustain practice around newly implemented interventions [34-36, 42, 47, 51, 53, 55, 64-66, 68, 69].

Intervention workability and functionality

A frequently adopted approach included using existing programs or frameworks (local or otherwise) as the basis for new intervention development [40, 41, 43-46, 69]. Literature suggested healthcare practices can flex to incorporate interventions designed as part of, or alongside, existing processes [3, 31, 38, 39, 46, 64, 68]. This approach was often more acceptable to staff, promoting incorporation into practice, adherence and behavioural change [3, 38, 39, 46]. Conversely, lack of transferability across healthcare settings was viewed as a barrier to implementation [40, 44]. The workability of interventions was important, with the integral use of guidelines and prompts facilitating intervention utility and accessibility [1, 3, 31, 32, 36, 39, 40, 42, 44, 47-49, 60-65, 67-71]. The use of stickers and brightly coloured forms for insertion into patient medical records promoted easy recognition of interventions [31, 40]. For many, successful incorporation into working practices appeared to rely upon the paper format of tools, their design and usability [1, 36, 49, 60, 64, 65].

Setting and context

Implementation often occurred within limited clinical settings, and the direct relevance of the specialism (to the intervention) was highlighted as important for promoting its value [32, 44-46, 60, 61, 63, 69]. Subsequent diffusion from implementation setting to hospital-wide acceptance was demonstrated in relation to a number of interventions [1, 31, 36, 69]. Despite this, multiple barriers and enablers to change were identified including: transferability across primary and secondary care settings [40, 64], staff turnover and management stability [41], availability of staff for updates, counter signatories to meet completion timeframes [39, 40, 44], time for communication processes [46], robustness of implementation sites [41, 69], and the difficulty in transforming clinician attitudes [45, 62, 69].

The clinical complexity and unpredictability surrounding the patients for whom the interventions were targeted often affected successful or opportune application [32, 46]. Utility was improved where deterioration was predictable [31, 32, 46], yet recognition of deterioration was on occasion inherently difficult [32]. Clinical uncertainty meant that at times interventions were not fully utilised for the patients they were designed to support [31, 46].

Perceived value and appraisal

Staff perceptions and the degree of alignment with the intended purpose of interventions, were infrequently reported [36, 62, 69]. Staff valuing the intervention from the outset had important implications for effective application [36, 39, 42, 46] and perceived utility, existing supportive local policy

For peer review only - http://bmjopen.bmj.com/site/about/guidelines.xhtml

and inclusive collaborative development were all highlighted as facilitators [36, 38, 39, 41, 46, 62, 65, 67, 69, 71]. At an organisational level, responsiveness to national guidance and policy was likely to promote value [3, 41, 65]. Understanding objectives early on in the implementation pathway was key, as misunderstandings were not uncommon [31, 39, 52], and could lead to reduced or incomplete application of interventions [31, 39].

Following implementation, appraisal of intervention value was frequent [3, 40, 41, 46-48, 50, 60, 61, 63, 69], often demonstrating communal agreement centred around positive impact on working practices [3, 33, 40, 41, 44, 46-48, 50, 60-62, 69]. Increased workload was expressed as a concern by staff, mainly relating to the completion of tools and the need for patient or family discussions [1, 3, 44, 60, 61, 69]. On appraisal, interventions were perceived to be a worthwhile investment due to patient benefit and the improved clarity and time saved, as a result of decisions and discussions taking place earlier in the care trajectory [1, 3, 61]. A number of papers recommended or proposed outcome measures relating to patient and relative experience [3, 31, 40, 47, 48, 62, 69, 71], but only a small number reported utilising such measures [1, 33, 49, 51].

For peer review only

For peer review only - http://bmjopen.bmj.com/site/about/guidelines.xhtml

For peer review only - http://bmjopen.bmj.com/site/about/guidelines.xhtml

Table 3. Distribution of themes by NPT construct

NPT Core Construct [16]	NPT Sub-Construct and Questions [16]	Themes: Input into development	Key clinical proponents	Training and education	Workability/ Functionality	Setting and context	Perceived value and appraisal
Coherence	Differentiation<br>- Does the intervention differ, & if so, how is it different from current practices?<br>- What work has been undertaken to aid understanding of how the new intervention differs from current practice?	■			■		
	Communal specification<br>- Do staff have a shared understanding of the purpose of the intervention?<br>- How has a shared understanding of the purpose of the intervention been built among staff within the organisation?	■		■			■
	Individual specification<br>- Do staff understand their specific tasks & responsibilities in relation to the intervention? What work has been done to ensure they are understood?<br>- Do individuals understand how the intervention will impact upon the nature of their work?			■			■
	Internalisation<br>- Do staff understand the potential value of the intervention for their work?<br>- What work has been done to promote understanding of the value, benefits & importance of the new intervention among staff & the organisation as a whole?	■				■	■
Cognitive participation	Initiation<br>- Are there key individuals identified as those driving the intervention forward?	■	■			■	
	Enrolment<br>- What work has been done to promote others to 'buy-in' & engage with the intervention, including training & education?<br>- Are people open to working with others in new ways to adopt the intervention?	■	■	■	■	■	■
	Legitimation<br>- Do staff believe that participating in the intervention is a legitimate part of their role?<br>- What work has been undertaken to ensure people believe it is right for them to be involved with the intervention?	■				■	■
	Activation<br>- Do people continue to support the intervention?<br>- What work has been undertaken to sustain the new intervention in practice & keep people supporting its use?		■	■		■	■
Collective action	Interactional workability<br>- Can the intervention be easily integrated into existing work practices?<br>- What work has been undertaken to allow the new intervention to integrate with existing staff & organisational practices?	■		■	■	■	
	Relational integration<br>- Do individuals feel confident in their own & others' abilities to use the intervention?<br>- What work has been undertaken to build knowledge & confidence in using the new intervention?<br>- Does the intervention impact upon working relationships & what work has been done to minimise this?	■		■	■	■	■
	Skill set workability<br>- Has the work (associated with the intervention) been assigned to those with the most appropriate skills?<br>- What formed the decision behind how the division of labour was allocated?<br>- Has sufficient training given staff the skills to enable them to use the intervention?		■	■	■	■	
	Contextual integration<br>- What resources have been provided to support the new intervention in practice?<br>- Is the intervention adequately supported at managerial level?		■	■	■		■
Reflexive monitoring	Systemization<br>- Has information been collected or are there plans to collect information to determine the usefulness of the intervention through feedback, audit or other means?			■			■

For peer review only

For peer review only - http://bmjopen.bmj.com/site/about/guidelines.xhtml

	- Have the effects of the intervention been reported back to those involved?						
	Communal appraisal<br>- Is there communal agreement among staff as to the value of the intervention?<br>- Has the worth of the intervention been evaluated collaboratively in formal or informal groups?						
	Individual appraisal<br>- What is the effect of the intervention on an individuals' workload?<br>- Do individuals value the effect it has on their individual work?						
	Reconfiguration<br>- Has appraisal work lead to attempts to improve or modify the intervention?						

For peer review only

For peer review only - http://bmjopen.bmj.com/site/about/guidelines.xhtml

4. DISCUSSION

In this review we have identified a broad and diverse literature focusing on the implementation of goals of care interventions. Findings from this review confirm these interventions are both complex and contentious in nature, and as such it is conceivable that what we have learnt here applies to other contentious interventions and processes, for example discharge planning.

Our analysis has led us to characterise the elements that constitute contentious interventions. Using the example of goals of care interventions, we propose that these interventions consist of three components that intersect at three different levels in a system of negotiated interactions:

1. Negotiated decision making between clinicians, patients and family members, which is localised and characterised by its meaning for the individual.
2. The organisational procedure and collective system of making negotiated decisions.
3. The socio-legal constraints of taking account of preferences (including consent and capacity) that define the parameters.

Review findings point to negotiated interaction processes taking place at between individuals. This mediates a set of procedures about how decisions should be made: a set of expectations about what procedures should be done, what negotiations are possible, and what these look like within and across organisations. Socio-legal conceptualisation involves how organisations must deal with these preferences. All of these continuous components interact and affect a patient's care trajectory.

From the six themes which emerged from our analysis, we have generated transferable learning outcomes for the implementation of contentious interventions. These are described below as a series of propositions relevant to goals of care, which we contend may apply across contentious interventions as a whole. These propositions are:

Individuals resist interventions that replicate the work of existing practices.

The value of incorporating interventions into working practices has previously been described [8] and lies in minimising disruption. The adoption of existing intervention formats and integral guidelines was popular, and may act to improve successful compliance and integration through increased staff familiarity and confidence. However there is a fine line, as individuals resist interventions that replicate the work of existing practices [47, 69]. Interventions have to be easily differentiated from other practices to be valued. There needs to be clarity regarding the practices that are discontinued, preventing unnecessary duplication, and clear identification of the benefits of the new intervention. Evidence from this review suggests that high visibility methods (for example a sticker or coloured background) are valued for quick identification and time saving in clinical practice. These are features unique to the paper based nature of tools. Technological infrastructure facilitating electronic access to one record in all contexts will enhance the likelihood of widespread embedding across organisations.

For peer review only - http://bmjopen.bmj.com/site/about/guidelines.xhtml

Contentious interventions are difficult to integrate in environments where there is clinical unpredictability and uncertainty.

Interventions aimed at improving care for patients facing uncertainty can be difficult to integrate due to the very nature of complexity that exists for these patients and their clinicians. Usually the application of skills and techniques introduce order into such situations, with clinicians seeking scripts to work to. However, clinical uncertainty can render these scripts ineffectual, leading to uncertain outcomes. In light of this, studies in this review suggest the importance of the intervention being delivered by a clinician who has an established relationship with the patient and/or knows their situation well [42, 44]. While this represents the ideal, in the reality of clinical practice and a highly pressurised health service, this may prove difficult to achieve.

Legitimacy is established where individuals build a shared understanding of purpose that enables them to attribute value to the intervention.

The use of senior clinicians and managers in the development process is recognised for building a shared vision [72]. However, the potential for authoritative dominance of certain clinicians is recognised [73], and efforts must be made to enable active contributions from key members of junior staff. A high degree of clinical ownership is recognised as important for successful implementation [74]. This is more likely to be achieved if all clinicians, irrespective of seniority, believe they have a role to play and are making a valid contribution [75]. Key clinical proponents are essential in building legitimacy, successful and sustained implementation, especially in the context of organisational instability [76, 77].

Training and education provide the framework for individuals to understand the value of what they do.

Training and education has an important role in facilitating a shared understanding of purpose, addressing both moral and technical aspects, which are important for building value in the intervention. Training and education serves to facilitate this, fostering clarity of value in the intervention.

The importance of training and education is not a new concept. However, there is a lack of agreement regarding optimal delivery methods, which may relate to a need for training to fit within the clinical context. Findings from the review point to the need for multi-modal educational programmes, repetition and timeliness of training, but this should be considered in the context of staff and financial resource implications [78, 79].

Appraisal work is critically important because it is how individuals value the intervention.

The importance of sharing appraisal outcomes, conveying purpose and value to others is evident, as value is often not realised until successful implementation is achieved. However, reporting of this in the literature was limited [47, 54]. In particular, evaluation of patient and family perspectives was notably absent in the papers included in this review. We suggest their inclusion is crucial for imputing value into interventions which seek to include them [80]. Surveys suggest that clinicians will foresee advantages and disadvantages in any proposed intervention [36, 46]. Therefore, sharing appraisal work, which addresses the positives and negatives, may be beneficial in providing a more balanced view of an intervention's value.

For peer review only - http://bmjopen.bmj.com/site/about/guidelines.xhtml

The transfer of contentious interventions to other settings is problematic.

The initial setting can influence implementation success and subsequent transfer to other locations. In our review there is limited evidence of transfer at scale, over multiple geographical sites [68]. There are multiple barriers through which interventions need to filter, including specialisms, care locations, and structural and cultural factors. In addition, groups of clinicians hold different values, skills and knowledge. This introduces problems of discretion, interpretation and enactment within different contexts and may limit scale up and transferability. While not widely addressed by the papers in this review, wider implementation literature suggests the importance of involving patients and caregivers when transferring and implementing interventions beyond formal clinical settings [81, 82].

In summary, improving decision-making around goals of care and ensuring patient preferences are taken into account requires a better understanding of the implementation processes and factors that promote or impede implementation. We have used the exemplar of goals of care interventions to elucidate propositions in relation to the successful implementation of contentious interventions, where success refers to the routine incorporation and embedding of intervention components into everyday practice. We believe these propositions to be transferable and generalizable beyond the remit of goals of care, to other contentious (and complex) healthcare interventions.

5. CONCLUSION

Findings from the review show that whilst such interventions are variable in design and use, there are a series of collective factors that influence successful implementation into routine clinical practice. We recommend that those seeking to introduce goals of care and other contentious interventions, consider the different facets of NPT, and use knowledge of this to develop implementation strategies. The contentious nature of these interventions means that their incorporation into everyday practice is dependent on a number of factors. Building a shared understanding of purpose that enables participants to attribute value to the intervention which is key, and both training and education and appraisal work play an important role in this process. Identifying clinical proponents who are able to, not only drive, but positively influence implementation is essential. Implementing complex and contentious interventions presents challenges which operate at an individual, organisational and systems level. It is these interaction level processes which make an intervention contentious, as well as making it challenging to introduce and embed. Success is more likely to occur, be established and sustained, if due attention is paid to the processes that facilitate operationalisation in the healthcare settings in which implementation occurs.

ACKNOWLEDGEMENTS

This review was funded by the National Institute for Health Research (NIHR) Collaboration for Leadership in Applied Health Research and Care Wessex. The views expressed are those of the authors and not necessarily those of the NHS, the NIHR or Department of Health. The authors would like to thank Dr Mark Banting for his assistance with data extraction in the initial phase of the review.

AUTHOR CONTRIBUTIONS

MM, CM and AR designed the review. AC performed searches. AC, and CM screened titles and abstracts and full paper screening was carried out by AC, MM and CM. Data extraction was performed by AC, MM

For peer review only - http://bmjopen.bmj.com/site/about/guidelines.xhtml

and SL. AC and MM drafted the manuscript with assistance from SL, CM, NC and AR. All authors critically reviewed the manuscript for intellectual content and approved the final version of the paper. AC is guarantor.

FUNDING

This work was supported by the National Institute for Health Research Collaboration for Leadership in Applied Health Research and Care (NIHR CLAHRC) Wessex which is a partnership between Wessex NHS organisations and partners and the University of Southampton. Funders had no role in study design, data collection and analysis, decision to publish or preparation of the manuscript.

DISCLAIMER

The views expressed are those of the author(s) and not necessarily those of the NHS, the NIHR or the Department of Health.

COMPETING INTERESTS

The authors declare no competing interests.

DATA SHARING STATEMENT

No additional data are available.

For peer review only

REFERENCES

1. Obolensky L, Clark T, Matthew G, Mercer M. A patient and relative centred evaluation of treatment escalation plans: a replacement for the do-not-resuscitate process. *J Med Ethics* 2010;36(9):518-520.
2. Findlay GP, Shotton H, Kelly K, *et al*. *Time to intervene? A review of patients who underwent cardiopulmonary resuscitation as a result of an in-hospital cardiorespiratory arrest.* A report by the National Confidential Enquiry into Patient Outcome and Death, 2012.
3. Fritz Z, Malyon A, Frankau JM, *et al*. The Universal Form of Treatment Options (UFTO) as an alternative to Do Not Attempt Cardiopulmonary Resuscitation (DNACPR) orders: a mixed methods evaluation of the effects on clinical practice and patient care. *PloS one* 2013;8(9):e70977.
4. Nicholson TR, Cutter W, Hotopf M. Assessing mental capacity: The mental capacity act. *BMJ* 2008;336(7639):322-325.
5. Craig P, Dieppe P, Macintyre S, *et al*. Developing and evaluating complex interventions: the new Medical Research Council guidance. *BMJ* 2008;337:a1655.
6. Council of Europe (1950) *European Convention for the Protection of Human Rights and Fundamental Freedoms, as amended by Protocols Nos. 11 and 14,* November 4 (ets, no. 5). Available at: http://www.refworld.org/docid/3ae6b3b04.html (Accessed: 08 March 2017).
7. May C, Finch T, Mair F, et al. Understanding the implementation of complex interventions in health care: the normalization process model. *BMC health services research* 2007;7(1):148.
8. May C. Agency and implementation: understanding the embedding of healthcare innovations in practice. *Social science & medicine* 2013;78:26-33.
9. Damschroder LJ, Aron DC, Keith RE, *et al*. Fostering implementation of health services research findings into practice: a consolidated framework for advancing implementation science. *Implementation science* 2009;4(1):1.
10. Moore GF, Audrey S, Barker M, *et al*. Process evaluation of complex interventions: Medical Research Council guidance. *BMJ* 2015;350:h1258.
11. May CR, Johnson M, Finch T. Implementation, context and complexity. *Implementation Science* 2016;11(1):141.
12. May C. Mobilizing modern facts: health technology assessment and the politics of evidence. *Sociology of Health & Illness* 2006;28(5):513-32.
13. Lund S, Richardson A, May C. Barriers to advance care planning at the end of life: an explanatory systematic review of implementation studies. *PLoS One* 2015;10(2):e0116629.
14. May C, Finch T. Implementing, embedding, and integrating practices: an outline of normalization process theory. *Sociology* 2009;43(3):535-554.
15. May CR, Mair F, Finch T, *et al*. Development of a theory of implementation and integration: Normalization Process Theory. *Implementation Science* 2009;4(1):1.
16. May C, Rapley T, Mair FS, *et al*. *Normalization Process Theory On-line Users' Manual, Toolkit and NoMAD instrument*. 2015. Available at: http://www.normalizationprocess.org.
17. Finch TL, Girling M, May CR, *et al*. *NoMAD: Implementation measure based on Normalization Process Theory*. [Measurement instrument]. 2015. Retrieved from http://www.normalizationprocess.org.
18. Pham MT, Rajić A, Greig JD *et al*. A scoping review of scoping reviews: advancing the approach and enhancing the consistency. *Research synthesis methods* 2014;5(4):371-385.
19. Davis K, Drey N, Gould D. What are scoping studies? A review of the nursing literature. *International journal of nursing studies* 2009;46(10):1386-1400.

For peer review only - http://bmjopen.bmj.com/site/about/guidelines.xhtml

20. Arksey H, O'Malley L. Scoping studies: towards a methodological framework. *International journal of social research methodology* 2005;8(1):19-32.
21. Levac D, Colquhoun H, O'Brien KK. Scoping studies: advancing the methodology. *Implementation Science* 2010;5(1):1.
22. Fritz Z, Cork N, Dodd A, *et al.* DNACPR decisions: challenging and changing practice in the wake of the Tracey judgment. *Clinical Medicine* 2014;14(6):571-576.
23. Perkins GD, Griffiths F, Slowther A-M, *et al.* Do-not-attempt-cardiopulmonary-resuscitation decisions: an evidence synthesis. *Health Services and Delivery Research* 2016;4(11).
24. *RESPECT: Recommended Summary Plan for Emergency Care and Treatment* (2017) Available at: www.respectprocess.org.uk (Accessed 05 July 2017).
25. Hsieh H-F, Shannon SE. Three approaches to qualitative content analysis. *Qualitative health research* 2005;15(9):1277-1288.
26. Watson R, Parr J, Joyce C, *et al*. Models of transitional care for young people with complex health needs: a scoping review. *Child Care Hlth Dev* 2011;37(6):780-791.
27. Johnson MJ, May CR. Promoting professional behaviour change in healthcare: what interventions work, and why? A theory-led overview of systematic reviews. *BMJ open* 2015;5(9):e008592.
28. Gallacher K, Morrison D, Jani B, *et al*. Uncovering treatment burden as a key concept for stroke care: a systematic review of qualitative research. *PLoS Med* 2013;10(6):e1001473.
29. Mair FS, May C, O'Donnell C, *et al*. Factors that promote or inhibit the implementation of e-health systems: an explanatory systematic review. *Bulletin of the World Health Organization* 2012;90(5):357-364.
30. May C, Sibley A, Hunt K. The nursing work of hospital-based clinical practice guideline implementation: an explanatory systematic review using Normalisation Process Theory. *International journal of nursing studies* 2014;51(2):289-299.
31. Carey I, Shouls S, Bristowe K, *et al*. Improving care for patients whose recovery is uncertain. The AMBER care bundle: design and implementation. *BMJ supportive & palliative care* 2015;5(1):12-18.
32. Etkind SN, Karno J, Edmonds PM, et al. Supporting patients with uncertain recovery: the use of the AMBER care bundle in an acute hospital. *BMJ supportive & palliative care* 2015;5(1):95-98.
33. Morris M, Briant L, Chidgey-Clark J, *et al*. Bringing in care planning conversations for patients whose recovery is uncertain: learning from the AMBER care bundle. *BMJ supportive & palliative care* 2011;1(1):72-72.
34. Nutt S, Lanaghan K. *Management of Patients who are End of Life*. Doncaster and Bassetlaw Hospitals NHS Foundation Trust, 2015.
35. Guy's and St Thomas' NHS Foundation Trust. *The AMBER care bundle – a guide for patients, their relatives and carers*. Patient information leaflet, 2012.
36. Fritz Z, Fuld JP. Development of the Universal Form Of Treatment Options (UFTO) as an alternative to Do Not Attempt Cardiopulmonary Resuscitation (DNACPR) orders: a cross-disciplinary approach. *J Eval Clin Pract* 2015;21(1):109-117.
37. *UFTO: Universal Form of Treatment Options* (2015) Available at: www.ufto.org (Accessed 01 July 2015).
38. *Universal Form of Treatment Options: Scientific Background* (2015) Available at: http://www.ufto.org/background/scientific-background/ (Accessed: 01 July 2015).
39. Brimblecombe C, Crosbie D, Lim WK, *et al*. The Goals of Patient Care project: implementing a proactive approach to patient-centred decision-making. *Intern Med J* 2014;44(10):961-966.
40. Hickman SE, Nelson CA, Moss AH, *et al*. Use of the Physician Orders for Life-Sustaining Treatment (POLST) paradigm program in the hospice setting. *J Palliat Med* 2009;12(2):133-141.

For peer review only - http://bmjopen.bmj.com/site/about/guidelines.xhtml

41. Hockley J, Watson J, Oxenham D, *et al*. The integrated implementation of two end-of-life care tools in nursing care homes in the UK: an in-depth evaluation. *Palliat Med* 2010;24(8):828-838.

42. Kaldjian LC, Broderick A. Developing a policy for do not resuscitate orders within a framework of goals of care. *Jt Comm J Qual Patient Saf* 2011;37(1):11-19.

43. Mbriwa K, Limaye S. HOW CAN WE IMPROVE 'DO NOT ATTEMPT CARDIOPULMONARY RESUSCITATION' (DNACPR) AND END OF LIFE CARE (EOLC) DECISION MAKING? *Age and Ageing* 2014:43(suppl_1): i10.

44. Newport KB, Patel S, Lyckholm L, *et al*. The "PSOST": Providers' Signout for Scope of Treatment. *J Palliat Med* 2010;13(9):1055-1058.

45. Piers RD, Benoit DD, Schrauwen WJ, *et al*. Do-not-resuscitate decisions in a large tertiary hospital: differences between wards and results of a hospital-wide intervention. *Acta Clinica Belgica* 2014;66:2,116-122.

46. Stockdale C, Trivedi B, Jerome E, *et al*. Implementation of a combined Cardiopulmonary Resuscitation and Treatment Escalation Plan document in a District General Hospital. *BMJ Quality Improvement Reports* 2013;2(2):u202653. w201236.

47. Dahill M, Powter L, Garland L, et al. Improving documentation of treatment escalation decisions in acute care. BMJ quality improvement reports 2013;2(1):u200617. w201077.

48. Powter L, Dahill M, Garland L, *et al. Improving decision making and documentation of resuscitation and ceiling of treatment decisions*. Available at: http://www.acutemedicine.org.uk/wp-content/uploads/2012/10/aqi28%20-%20improving%20decision%20making%20and%20documentation%20of%20resuscitation%20and%20ceiling%20of%20treatment%20decisions.pdf (Accessed 27 June 2016).

49. Mercer MH. The death of DNR: Treatment escalation plans. (Letter to the editor). *BMJ* 2009;338:b2020.

50. Obolensky L, Mercer M. (2007) Replacing Do-Not-Resuscitate Forms with Treatment Escalation Plans. *The Intensive Care Society Annual Spring Meeting*.

51. Obolensky L, Mercer M. (2007) Patients' Experiences of Treatment Escalation Plans. *The Intensive Care Society Annual Spring Meeting.*

52. Karakusevic C. *Devon Treatment Escalation Plan & Resuscitation Decision Record (TEP).* Available at: http://www.macmillan.org.uk/documents/aboutus/health_professionals/primarycare/showcasing/2013/treatment-escalation-and-resuscitation-devon.pdf (Accessed: 23 June 2016).

53. Gray C. *Treatment Escalation Plans (TEP) and Resuscitation Decision Records*. Torbay and Southern Devon Health and Care NHS Trust, 2014.

54. Care Quality Commission. *North Devon Healthcare NHS Trust End of Life Care Quality Report*. End of Life Care Quality Report, 2014.

55. Human S, Scott K. *Treatment Escalation Plan (TEP) Forms.* Rowcroft Hospice, 2013.

56. Devon LMC Newsletter. *Treatment Escalation Plans (TEP) – new update*. Newsletter article, August 2013.

57. Hill S, Mitchell P, Cooper J, *et al*. *New TEP form (Version 10) – Frequently Asked Questions*. Macmillan Cancer Support, St Luke's Hospice Plymouth, East and West Devon Clinical Commissioning Group.

58. Diamond A. *Briefing note on the use of Treatment Escalation Plans (TEPs).* North Devon Healthcare NHS Trust.

59. North Devon Healthcare NHS Trust. *Guidance for completing Treatment Escalation Plan and Resuscitation Decisions*. Available at: http://www.northdevonhealth.nhs.uk/wp-content/uploads/2015/07/V10-FINAL-Guidance-for-TEP-and-Flowchart.pdf (Accessed 21 March 2017).

For peer review only - http://bmjopen.bmj.com/site/about/guidelines.xhtml

60. Paes P, O'Neill C. Treatment escalation plans–a tool to aid end of life decision making? *BMJ Supportive & Palliative Care* 2012;2(Suppl 1):A60-A60.
61. Paes P. *Treatment Escalation Planning - Introducing systematic senior clinical decision making.* Available at: http://www.karenpicking.co.uk/images/Paul%20Paes%20-%20project %20poster.pdf (Accessed: 23 June 2016).
62. Hannah J. (2014) *One TEP at a time.... Treatment Escalation Plans in Medical High Dependency.* Available at: http://www.qihub.scot.nhs.uk/media/618301/ julie%20hannah.pdf (Accessed: 08 March 2017).
63. Thomson B, Kok K, Harrod E, *et al*. *Introducing Treatment Escalation Plans in Acute Medicine*. Available at: http://www.acutemedicine.org.uk/wp-content/uploads/2014/10/AQI-40-Introducing-Treatment-Escalation-Plans-in-Acute-Medicine-A-Quality-Improvement-Project.pdf (Accessed: 23 June 2016).
64. Over J. *Treatment Escalation Plan and Resuscitation Decision Record (in relation to the adult patient over 18 years)*. Royal Cornwall Hospitals NHS Trust, 2015.
65. West Suffolk NHS Foundation Trust Resuscitation Committee. *Escalation Plan and Resuscitation Status (EPARS) (including DNACPR).* West Suffolk NHS Foundation Trust, 2013.
66. Basildon and Thurrock University Hospitals NHS Foundation Trust Critical care. *Treatment Escalation Plan (TEP*). Basildon and Thurrock University Hospitals NHS Foundation Trust, 2014.
67. Downar J, Hawryluck L. What should we say when discussing "code status" and life support with a patient? A Delphi analysis. *J Palliat Med* 2010;13(2):185-195.
68. Bailey FA, Williams BR, Woodby LL, *et al*. Intervention to improve care at life's end in inpatient settings: the BEACON trial. *J Gen Intern Med* 2014;29(6):836-843.
69. Bookbinder M, Blank AE, Arney E, *et al*. Improving end-of-life care: development and pilot-test of a clinical pathway. *J Pain Symptom Manage* 2005;29(6):529-543.
70. Bernacki RE, Block SD; American College of Physicians High Value Care Task Force. Communication about serious illness care goals: a review and synthesis of best practices. *JAMA Intern Med* 2014;174(12):1994-2003.
71. Hoerger M, Epstein RM, Winters PC, *et al*. Values and options in cancer care (VOICE): study design and rationale for a patient-centered communication and decision-making intervention for physicians, patients with advanced cancer, and their caregivers. *BMC Cancer* 2013;13:188.
72. Markoczy L. Consensus formation during strategic change. *Strategic Management Journal* 2001;22(11):1013-1031.
73. Nugus P, Greenfield D, Travaglia J, *et al*. How and where clinicians exercise power: Interprofessional relations in health care. *Social science & medicine* 2010;71(5):898-909.
74. Richens Y, Rycroft-Malone J, Morrell C. Getting guidelines into practice: a literature review. *Nursing Standard* 2004;18(50):33-40.
75. Checkland PB. (1981) *Systems thinking, systems practice*. Chichester: John Wiley.
76. Stetler CB, Legro MW, Rycroft-Malone J, *et al*. Role of "external facilitation" in implementation of research findings: a qualitative evaluation of facilitation experiences in the Veterans Health Administration. *Implementation Science* 2006;1(1):1.
77. Locock L, Dopson S, Chambers D, *et al*. Understanding the role of opinion leaders in improving clinical effectiveness. *Social science & medicine* 2001;53(6):745-757.
78. Phillipson L, Goodenough B, Reis S, *et al*. Applying Knowledge Translation Concepts and Strategies in Dementia Care Education for Health Professionals: Recommendations From a Narrative Literature Review. *Journal of Continuing Education in the Health Professions* 2016;36(1):74-81.

For peer review only - http://bmjopen.bmj.com/site/about/guidelines.xhtml

79. Field RA, Fritz Z, Baker A, *et al*. Systematic review of interventions to improve appropriate use and outcomes associated with do-not-attempt-cardiopulmonary-resuscitation decisions. *Resuscitation* 2014;85(11):1418-1431.

80. National Institute for Health Research. *Patients and the public*. 2016. Available at: http://www.nihr.ac.uk/patients-and-public/ (Accessed 04 November 2016).

81. May C, Montori VM, Mair FS. We need minimally disruptive medicine. *BMJ* 2009;339:b2803.

82. Shippee ND, Shah ND, May CR, *et al*. Cumulative complexity: a functional, patient-centered model of patient complexity can improve research and practice. *Journal of clinical epidemiology* 2012;65(10):1041-1051.

For peer review only

For peer review only - http://bmjopen.bmj.com/site/about/guidelines.xhtml

Figure 1. Flow diagram of literature screening

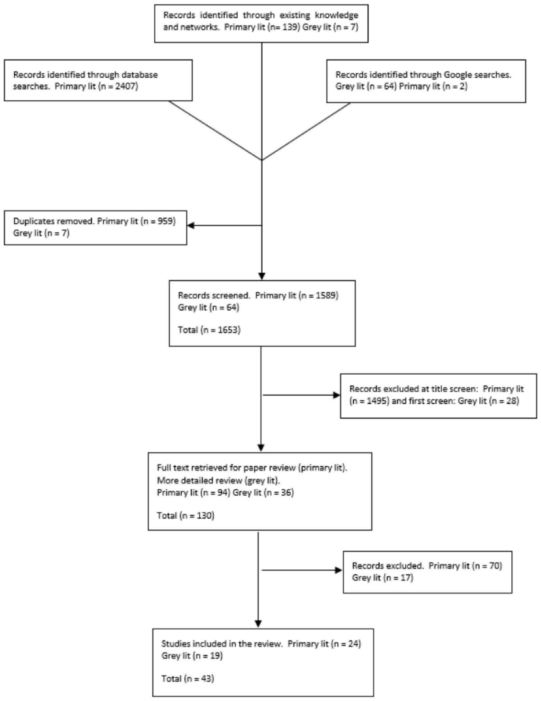

210x297mm (300 x 300 DPI)

Appendix 1: ***Primary literature search strategy***

Combined key word searches		Number of results in MEDLINE	3rd key word added if results >200
End of life care	Goals of care	(298) 12	Treatment decision making
End of life care	Ceiling of care	5	
End of life care	Ceiling of treatment	1	
End of life care	Treatment limitations	12	
End of life care	Resuscitation	(545) 11	Treatment decision making
End of life care	DNACPR	(859) 79	Goals of care
End of life care	Treatment escalation plans	0	
End of life care	Treatment decision making	68	
End of life care	Advance care plans	28	
End of life care	Supported decision making	8	
End of life care	End of life decisions	(624) 4	Treatment limitations for Medline, goals of care for CINAHL
End of life care	Treatment goals	56	
Rapid response	Goals of care	11	
Rapid response	Ceiling of care	0	
Rapid response	Ceiling of treatment	0	
Rapid response	Treatment limitations	5	
Rapid response	Resuscitation	(239) 2	Treatment limitations or End of life decisions
Rapid response	DNACPR	(549) 5	Palliative care
Rapid response	Treatment escalation plans	0	
Rapid response	Treatment decision making	4	
Rapid response	Advance care plans	0	
Rapid response	Supported decision making	0	
Rapid response	End of life decisions	1	
Rapid response	Treatment goals	5	
Medical emergency	Goals of care	53	
Medical emergency	Ceiling of care	0	
Medical emergency	Ceiling of treatment	2	
Medical emergency	Treatment limitations	8	
Medical emergency	Resuscitation	(6,041) 11	End of life decisions
Medical emergency	DNACPR	(2,291) 7	End of life decisions or Treatment goals
Medical emergency	Treatment escalation plans	0	
Medical emergency	Treatment decision making	26	
Medical emergency	Advance care plans	0	

For peer review only

Medical emergency	Supported decision making	4	
Medical emergency	End of life decisions	22	
Medical emergency	Treatment goals	17	
Critical care outreach team	Goals of care	0	
Critical care outreach team	Ceiling of care	0	
Critical care outreach team	Ceiling of treatment	0	
Critical care outreach team	Treatment limitations	0	
Critical care outreach team	Resuscitation	2	
Critical care outreach team	DNACPR	0	
Critical care outreach team	Treatment escalation plans	0	
Critical care outreach team	Treatment decision making	0	
Critical care outreach team	Advance care plans	0	
Critical care outreach team	Supported decision making	0	
Critical care outreach team	End of life decisions	0	
Critical care outreach team	Treatment goals	0	
Palliative care	Goals of care	(633) 27	End of life decisions or Treatment decision making
Palliative care	Ceiling of care	4	
Palliative care	Ceiling of treatment	0	
Palliative care	Treatment limitations	34	
Palliative care	Resuscitation	(599) 54	Goals of care
Palliative care	DNACPR	(2,232) 84	Goals of care
Palliative care	Treatment escalation plans	0	
Palliative care	Treatment decision making	146	
Palliative care	Advance care plans	12	
Palliative care	Supported decision making	3	
Palliative care	End of life decisions	(359) 28	Treatment decision making or Goals of care
Palliative care	Treatment goals	182	
Clinical deterioration	Goals of care	5	
Clinical deterioration	Ceiling of care	0	
Clinical deterioration	Ceiling of treatment	0	
Clinical deterioration	Treatment limitations	1	
Clinical deterioration	Resuscitation	67	

For peer review only

Clinical deterioration	DNACPR	220	N/A (adding 3rd term gave 0 results)
Clinical deterioration	Treatment escalation plans	0	
Clinical deterioration	Treatment decision making	3	
Clinical deterioration	Advance care plans	0	
Clinical deterioration	Supported decision making	1	
Clinical deterioration	End of life decisions	0	
Clinical deterioration	Treatment goals	7	
Treatment escalation plans	--------------------	10	
Advance care plans	--------------------	57	
Treatment decision making	Resuscitation	49	
Treatment limitations	Resuscitation	25	

For peer review only

Appendix 2: *Data extraction tool*

ARTICLE DETAILS				
Study Title				
Primary Author				
Year				
Country				
Setting	Community/Population		Nursing home/Residential care	
	Hospital/Secondary		General Practice/Primary care	
	Other (specify):			
Study design				
Participant(s)				
What is the research objective?				
Is the study describing a new/existing decision or communication <u>tool</u>?	Yes	Name of tool:	No	
What are the decisions of interest being studied? (e.g. DNACPR/active medical care/palliative care)	DNACPR		Other (specify):	
	Active Medical Care			
	Supportive/Palliative Care			
What are the core components of the tools/intervention being studied?	Escalation levels/interventions		Record of who plan discussed was discussed	
	Resuscitation status		Indication of person recording discussion and decision	
	Document rationale for treatment decision and resuscitation status		Mental capacity assessment	
	Review/discontinuation date		Prompt to record completion in notes (relating to separate tool only)	

For peer review only

For peer review only - http://bmjopen.bmj.com/site/about/guidelines.xhtml

Who makes decisions (e.g. consultant, junior doctors) and who is involved in decision-making (e.g. patients and families)?				
What is the trigger to initiating the process of decision making and/or what factors are taken into consideration? (e.g in advance of deterioration/in crisis or emergency situation)	All patients within study setting		Selected patients within study setting	
**Coherence**<br><br>*Sense making work individually and collectively when faced with the problem of operationalizing an intervention*	1) Differentiation: Does the intervention differ, and if so, how is it different from current practices? What work has been undertaken to aid understanding of how the new intervention differs from current practice?			
	2) Communal specification: Do staff have a shared understanding of the purpose of the intervention? How has a shared understanding of the purpose of the intervention been built among staff within the organisation?			
	3) Individual specification: Do staff understand their specific tasks and responsibilities in relation to the intervention? What work has been done to ensure individual tasks and responsibilities are understood. Do individuals understand how the intervention will impact upon the nature of their work?			
	4) Internalization: Do staff understand the potential value of the intervention for their work? What work has been done to promote understanding of the value, benefits and importance of the new intervention among staff and the organisation as a whole?			
**Cognitive participation**<br><br>*Relational work people do to build and sustain a community of practice around a new intervention*	1) Initiation: Are there key individuals identified as those driving the intervention forward?			
	2) Enrolment: What work has been done to promote others to 'buy-in' and engage with the intervention, including training and education? Are people open to working with others in new ways to adopt the intervention?			

For peer review only

	3) Legitimation: Do staff believe that participating in the intervention is a legitimate part of their role? What work has been undertaken to ensure people believe it is right for them to be involved with the intervention?
	4) Activation: Do people continue to support the intervention? What work has been undertaken to sustain the new intervention in practice and keep people supporting it's use?
**Collective action**<br><br>*The operational work that people do to enable new practices*	1) Interactional workability: Can the intervention be easily integrated into existing work practices? What work has been undertaken to allow the new intervention to integrate with existing staff and organisational practices?
	2) Relational integration: Do individuals feel confident in their own and others' abilities to use the intervention? What work has been undertaken to build knowledge and confidence in using the new intervention? Does the intervention impact upon working relationships and what work has been done to minimise this?
	3) Skill set workability: Has the work (associated with the intervention) been assigned to those with the most appropriate skills? What formed the decision behind how the division of labour was allocated? Has sufficient training given staff the skills to enable them to use the intervention?
	4) Contextual integration: What resources have been provided to support the new intervention in practice? Is the intervention adequately supported at managerial level?
**Reflective monitoring**<br><br>*The appraisal work that people do to assess and understand the ways a new intervention affects them and others around them*	1) Systemization: Has information been collected or are there plans to collect information to determine the usefulness of the intervention through feedback, audit or other means? Have the effects of the intervention been reported back to those involved?
	2) Communal appraisal: Is there communal agreement among staff as to the value of the intervention? Has the worth of the intervention been evaluated collaboratively in formal or informal groups?

For peer review only

	3) Individual appraisal: What is the effect of the intervention on an individuals' workload? Do individuals value the effect it has on their individual work?
	4) Reconfiguration: Has appraisal work lead to attempts to improve or modify the intervention?
Do patients and families understand the purpose of the intervention? What work has been done to increase their understanding?	
What is the work that patients have to do and do they involve others in the work (eg. family members)?	
Has feedback from patient/families been sought? Do they see the value of the intervention?	
What are the types of outcomes measured by the study?	
Other	

For peer review only

Appendix 3: The constructs and sub-constructs of NPT [16]

Core Constructs		Sub-constructs	
**Coherence**	The work required to make sense of:<br>a) an intervention, both individually and collectively as a healthcare professional, clinical care team, service and/or organization.<br>b) what is required to individually and collectively implement the intervention (in this case a tool and/or process to support decision making regarding goals of care in the context of pathophysiological deterioration)	**Differentiation**	Understanding how a new set of practices are different from current practices
		**Communal Specification**	How people work together to build a shared understanding of the aims, objectives and expected benefits of a set of new practices
		**Individual Specification**	Work that will help individuals understand their specific tasks and responsibilities around a set of practices
		**Internalization**	Work to understand the value, benefits and importance of a set of practices
**Cognitive Participation**	The relational work required within a clinical care team, service and/or organization to establish and sustain the particular intervention	**Initiation**	Work to drive the new set of practices forward
		**Enrolment**	Organizing or reorganizing of participants in order to collectively contribute to the work involved in new practices
		**Legitimation**	The work of ensuring that participants believe it is right for them to be involved and that they can make a valid contribution
		**Activation**	Actions and procedures needed to sustain a practice and maintain participant involvement
**Collective Action**	The operational work of organising and preparing the care environment, such as a	**Interactional Workability**	The interactional work that people do with each other and with other elements of a set of practices, when they seek to operationalise them in everyday settings

For peer review only

	particular service or organisation, to enact the new practices required to deliver the intervention	**Relational Integration**	The knowledge work that people do to build accountability and maintain confidence in a set of practices and in each other as they use them
		**Skill set Workability**	The allocation work that underpins the division of labour that is built up around a set of practices
		**Contextual Integration**	Managing a set of practices through the allocation of different kinds of resources and the execution of protocols, policies and procedures
**Reflexive Monitoring**	The appraisal work that people do to assess the acceptability and utility of the intervention, as well as the outcomes of the intervention and its delivery on those who negotiate and enact it (clinicians, individual patients and their families/carers)	**Systemization**	The work of collecting information to determine the effectiveness and usefulness of a new set of practices
		**Communal appraisal**	Participants working together in formal collaboratives or in informal groups to evaluate the worth of a set of practices
		**Individual appraisal**	Participants in a new set of practices work as individuals to appraise its effects on them and the contexts in which they are set
		**Reconfiguration**	Appraisal work by individuals or groups may lead to attempts to redefine procedures or modify practices

For peer review only

Appendix 4: ***Summary of findings by NPT construct***

Core NPT Construct [16]	NPT Sub-Construct and Questions [16]	Key Findings	Identified Themes
Coherence	*Differentiation*<br>- Does the intervention differ, & if so, how is it different from current practices?<br>- What work has been undertaken to aid understanding of how the new intervention differs from current practice?	- High clarity & description of differentiation, & where relevant, explanation of aspects that were similar to or based upon existing practice: based upon existing national programmes of work [40, 44], frameworks [41] or guidelines [69]; established from the format of locally used tools [44], adapted from local trusts [46] or consisted of adaptations to pre-existing interventions [43, 45, 46]; entirely new interventions developed [1, 3, 31, 39, 50-52, 67], usually resulting from extensive scoping work [31, 36, 67].<br>- Interventions were developed to replace or complement existing practices or tools [1, 3, 33, 39, 41, 43, 46, 50-52].<br>- To understand how & why interventions should differ from current practices the following were drawn upon: case note reviews [46]; staff interviews [36]; scoping of published literature & guidelines [42, 67, 69]; review of local practices & contexts [31, 36, 47, 48, 61, 68].	Workability/functionality<br>Input into development
	Communal specification<br>- Do staff have a shared understanding of the purpose of the intervention?<br>- How has a shared understanding of the purpose of the intervention been built among staff within the organisation?	- Number of common themes highlighted as the key purposes of interventions: reducing unnecessary or inappropriate treatment [39, 44, 65]; enabling consideration of goals of care [31, 39, 42], guiding treatment planning & decision making [31, 39, 67, 71]; improving communication & discussions between patients & clinicians [31, 39, 40, 65, 71]; respecting patient wishes & ensuring appropriate care [31, 40, 44, 53]; improving the management of uncertainty & advanced planning [31, 65]; guiding smooth transitions of care out-of-hours & between different clinical settings [44, 46, 53, 62, 64]; improving documentation, accessibility & awareness of treatment plans [44, 46, 53, 62, 65]; providing uniformity & continuity of care [44, 53, 62, 64, 65].<br>- Background evidence & scoping work has the secondary purpose of informing staff as to the rationale & purpose of interventions [3, 31, 38, 42, 43, 46-48, 53, 67, 70].<br>- Multi-disciplinary collaboration in both development [3, 36, 46-48, 52] & education provision [42, 52, 68].<br>- Accessing alternative education programmes can act as a barrier to understanding purpose [69].<br>- Staff perception of purpose only minimally described [36, 62, 69], & some evidence of staff misunderstanding purpose [31, 39, 52].	Perceived value & appraisal<br>Input into development<br>Training & education
	Individual Specification<br>- Do staff understand their specific tasks & responsibilities in relation to the intervention? What work has been done to ensure they are understood?<br>- Do individuals understand how the intervention will impact upon the nature of their work?	- Importance of training [36, 48, 52, 68] & tailored education [36, 39] in facilitating staff understanding of tasks & responsibilities.<br>- Lack of training resulting in differing interpretations of an individuals' responsibilities for the same intervention [52].<br>- Lack of understanding of responsibilities or discomfort with proposed tasks leading to ineffective or reduced use [39-41, 54, 69].<br>- Methods of training delivery: incorporation into induction programmes [48]; simulated clinical scenarios [36]; presentations & meetings [68]; provision of material resources [36, 68].	Training & education<br>Perceived value & appraisal
	Internalization<br>- Do staff understand the potential value of the intervention for their work?<br>- What work has been done to promote understanding of the value, benefits &	- Importance of implementation staff understanding the value of an intervention [36, 39, 42, 46].<br>- Foreseen advantages included prompting consideration of more forms of treatment & reducing the negative connotations of DNACPR [36, 46].<br>- Foreseen disadvantages included potential for staff confusion, committing patients to overtreatment & potential patient distress [36].<br>- Implementation area appears important in promoting value, & more challenging in specialities less used to practicing the concepts involved in the intervention [45, 69].	Perceived value & appraisal<br>Setting & context

	importance of the new intervention among staff & the organisation as a whole?	- Staff surveys [36, 46, 62, 71] & collaborative development [36, 38, 65, 67, 69] generated consensus as to the intervention's importance, & promoted value, often based on problems encountered in current practice [46, 71].<br>- National guidance & policy were drawn upon to highlight current inadequacies in practice interventions were designed to address [3, 41, 65]. Established local policy influenced the value attributed to an intervention [41].	Input into development
Cognitive Participation	*Initiation*<br>- Are there key individuals identified as those driving the intervention forward?	- Majority of interventions piloted or implemented within limited settings or specific services [32, 44, 46, 60, 61, 63], often with input into the design from staff within these settings [44, 46, 63].<br>- Key clinical proponents [41, 54, 69] or multidisciplinary teams [68] were used to liaise & orchestrate implementation over multiple sites.<br>- Interventionist/facilitator contact important in orientating & maintaining momentum of local leadership [41, 68, 69].<br>- Input into design & development from: specialists within relevant specialties [31, 41, 46, 67] & multidisciplinary contributors, gaining collective knowledge from all potential users [31, 36, 47, 48, 62].<br>- Senior clinicians or management responsible for compliance with new interventions [54, 64, 69].	Key clinical proponents<br>Input into development<br>Setting & context
	Enrolment<br>- What work has been done to promote others to 'buy-in' & engage with the intervention, including training & education?<br>- Are people open to working with others in new ways to adopt the intervention?	- Training & education encouraged 'buy-in' [3, 31, 36, 41, 42, 48, 53, 54, 68, 69]: incorporated into established working practices [42, 48, 68, 69]; led by senior clinicians from relevant specialties [31, 41, 69]; consisted of multiple modalities [31, 41, 42, 68, 69].<br>- Timeliness of training delivery: prior to implementation [3, 36]; alongside implementation [41, 42, 53, 54, 68, 69]; repeated training [31, 69].<br>- Participation in development shown to promote 'buy-in' [36, 41, 45, 46, 48, 67]. Different techniques were adopted: anonymity of input [45, 67]; individual input in isolation, or prior to group consultation [36, 45, 46, 67]; actively involving those opposed to the intervention [36]; seeking opinions from staff experienced in the subject area targeted by the intervention [31, 36, 46, 67]; input into the intervention design [46, 47, 69] & implementation process [41, 46] from staff/specialists working within implementation settings.<br>- Difficulty in transforming clinician attitudes can act as a barrier to engagement [45].	Workability/functionality<br>Key clinical proponents<br>Training & education<br>Perceived value & appraisal<br>Input into development<br>Setting & context
	Legitimation<br>- Do staff believe that participating in the intervention is a legitimate part of their role?<br>- What work has been undertaken to ensure people believe it is right for them to be involved with the intervention?	- Legitimation was important, but its extent among clinicians varied [43, 61, 69].<br>- Facilitators of legitimation: prior use of the intervention [41]; perceived usefulness of the intervention [60, 61]; opportunities to provide input or suggest modifications to the intervention, irrespective of seniority [36, 46, 47, 67].<br>- Legality appeared as both a barrier & facilitator to legitimation [45, 46]: participation related to a sense of legal duty as opposed to behaviour change [45] & concern for potential legal ramifications were a barrier for clinicians [46].	Perceived value & appraisal<br>Input into development<br>Setting & context
	Activation<br>- Do people continue to support the intervention?<br>- What work has been undertaken to sustain the new intervention in practice & keep people supporting its use?	- Evidence of diffusion of uptake & cultural change across settings [1, 31, 36, 68, 69].<br>- Follow-up resources (including guidelines & policy), & mentorship facilitated implementation & continued support of interventions [34, 42, 47, 53, 64, 65, 68, 69].<br>- Consideration given to specialty & robustness of implementation site [41, 69].<br>- Some evidence of poor completion or failure to honour interventions [40, 52].	Setting & context<br>Key clinical proponents<br>Training & education<br>Perceived value & appraisal
Collective action	*Interactional Workability*<br>- Can the intervention be easily integrated into existing work practices?<br>- What work has been undertaken to allow the new intervention to integrate with existing staff & organisational practices?	- Ability to integrate with existing working practices recognised as important [31, 36, 68].<br>- Facilitators to integration: integration as part of, or in conjunction with, existing practices [3, 31, 38, 39, 46, 64, 68]; usefulness, pragmatism & accessibility of intervention [1, 3, 36, 44, 47-49, 60, 64, 65, 71]; involvement of users in design & piloting, allowing for amendments to facilitate utility [36, 41, 46, 47]; format of intervention based on existing design [44, 46]; transferability across healthcare settings [40, 44, 64].<br>- Barriers to integration: logistical & staffing barriers including staff turnover, management changes & staff availability to complete interventions [39-41, 44]; replication of existing work [47, 69]; clinical complexity [32, 46].	Workability/functionality<br>Input into development<br>Setting & context<br>Training & education

For peer review only

		- Interactional workability of training was an important consideration [69, 71].<br>- Electronic medical health records (EMR): for some, configuration of interventions within EMR facilitated implementation [42, 68], whilst others described poor navigation of interventions within EMR [36, 52].	
	Relational Integration<br>- Do individuals feel confident in their own & others' abilities to use the intervention?<br>- What work has been undertaken to build knowledge & confidence in using the new intervention?<br>- Does the intervention impact upon working relationships & what work has been done to minimise this?	- Circulation of interventions during development/early implementation appeared important for building confidence in their use [31, 36, 44, 52, 62] & allowed for amendments to facilitate understanding [52].<br>- Low confidence in particular aspects of interventions: capacity decisions & determining goals of care [39, 52].<br>- Reduced confidence in others: the ability of clinicians from other specialties to make decisions [36]; potential prematurity of decisions [36]; evidence of interventions not being followed due to disagreement by on-call clinicians [46].<br>- Increased confidence in others: clinicians keen to involve other clinicians & nurses expressed their confidence in others [36].<br>- Evidence of better teamwork & communication especially out-of-hours, driven by improved clarity & consistency of decisions [3, 31, 38, 44].<br>- Multi-modal format of interventions consisting of tools, prompts, algorithms & guidelines, invoke confidence in their application [1, 3, 31, 32, 36, 39, 40, 44, 48, 61-63, 67-70].<br>- Multidisciplinary educational programmes help build knowledge & confidence across disciplines [68, 69].	Workability/functionality<br>Input into development<br>Perceived value & appraisal<br>Setting & context<br>Training & education
	Skill set Workability<br>- Has the work (associated with the intervention) been assigned to those with the most appropriate skills?<br>- What formed the decision behind how the division of labour was allocated?<br>- Has sufficient training given staff the skills to enable them to use the intervention?	- Division of work based on level & nature of responsibility, among nurses, senior & junior clinicians [1, 3, 31-33, 36, 39-46, 67-71].<br>- Delegation often controlled for as the minimum level of clinicians permitted to complete or authorise was specified [1, 3, 36, 42, 46, 48, 62-65, 69].<br>- Colleagues should delegate if they do not have adequate skills [1], but concern was apparent if staff delegated to lacked the necessary experience/skills [42].<br>- Training & education was utilised [31, 39, 41, 68, 69], proposed [70, 71], or identified as deficient [40, 45], in facilitating staff development of skills, & application of skills to the processes involved in using interventions. The following were identified as important: skills training [39, 40, 42, 45, 52, 68, 69, 71]; communication skills (highlighted as difficult, important to clinicians & requiring development) [31, 33, 39, 45, 71]; other skills including: assessment & shared understanding of goals of care [1, 31, 33, 39, 45, 71]; training in application of skills to intervention [40, 52, 54, 64, 68, 69]; having someone in a position of responsibility for training execution/attendance [64, 68, 69]; different educational materials used alongside training programmes [68, 71]; value of not over-assuming existing skillset [52].<br>- Different types of training to teach relevant skills [31, 33, 68, 69]: teaching rounds [31, 68, 69] & formal group sessions [31, 69].<br>- Consideration of experience versus skill, & the interplay of external patient & organisational factors [42, 44].	Setting & context<br>Workability/functionality<br>Training & education<br>Key clinical proponents

For peer review only

	Contextual Integration<br>- What resources have been provided to support the new intervention in practice?<br>- Is the intervention adequately supported at managerial level?	- Patient & carer information leaflets are important [35, 36, 42, 51, 55, 66] & encourage shared communication [38, 64].<br>- A variety of staff guidance resources & updates used: newsletter updates, frequently asked questions documents, briefing notes, guidebooks, pocket cards, checklists, guidelines, frameworks, DVDs, prompt lists, algorithms, procedural leaflets [36, 42, 56-59, 64, 65, 68-70].<br>- A number of interventions also functioned as guidelines, with the use of flowcharts, prompts & checklists [1, 3, 31, 32, 36, 39, 40, 42, 44, 48, 61-63, 67-70], described as encouraging full & accurate completion [31, 42, 70].<br>- Joint patient & staff resources (poster & brochure) [36, 42].<br>- Guidelines incorporated in trust policy documents [36, 42, 64, 65, 68-70].<br>- Evidence from training scenarios used to shape information guidance, in order to address misconceptions evident during training [36].<br>- Guidance was distributed: alongside training sessions [71]; in staff inductions or updates [56, 64]; in local policy documents [65]; during or alongside implementation [36, 57-59, 68, 69]; as part of the intervention itself [69, 70].<br>- Websites: UFTO intervention has its own website providing access to resources, background to development, guidance for use & updates [37, 38]. One study refers to a palliative care website as a resource for implementation [69].<br>- Policy & protocol: three intervention specific policy documents [53, 64, 65]; one end of life care policy document referring to the intervention [34]; most relate to resuscitation & escalation planning [42, 47, 53, 64, 65], but also include trust induction policies [64] & EOLC policies [34].<br>- Evidence of managerial acceptance: approval from heads of department & medical directors [48, 62]; nationwide implementation [31]; incorporation into policy & protocol [34, 42, 47, 53, 64, 65].<br>- Managerial support acts as a catalyst to instigating & maintaining implementation [41, 68, 71].<br>- Levels of contact with management, & influence of management culture recognised [41, 69].	Training & education<br>Workability/functionality<br>Perceived value & appraisal<br>Key clinical proponents
Reflexive Monitoring	*Systemization*<br>- Has information been collected or are there plans to collect information to determine the usefulness of the intervention through feedback, audit or other means?<br>- Have the effects of the intervention been reported back to those involved?	- All describe or propose the collection of information relating to measures of usefulness [1, 3, 31-33, 38-43, 45-52, 54, 60-63, 68, 69].<br>- Data collection is proposed in studies where interventions have not yet been implemented [36, 44, 67, 70, 71].<br>- Different data collection methods: medical record audit [32, 39-41, 43, 46, 60, 61, 63, 68, 69], in the majority executed pre & post implementation, or in comparison with control groups [41, 43, 60, 61, 63, 68, 69]; staff surveys or interviews, often used in combination with medical record audit [3, 40, 41, 46-48, 50, 60, 61, 63, 69]; direct ward based observation [3]; patient interviews [1, 51].<br>- Different outcomes assessed: clinical impact of interventions (e.g. patient deaths) [3, 31, 32, 61]; clinical factors (e.g. illness trajectory) related to the use of interventions [32]; changes in end of life care processes, behaviours & outcomes (e.g. opioid prescriptions) [45, 46, 68, 69]; rates of completion, adherence & compliance with new interventions [31, 39-41, 43, 45, 46, 61, 69], & specifically with regard to documentation [39, 47, 48, 63]; new staff knowledge of care principles associated with the intervention [69]; attitudes toward the intervention [40, 63]; perceived effects of the intervention on practice [41, 50]; practicalities of the intervention [60, 61].<br>- Specific measures: study specific set of evaluation tools to measure feasibility [69] & validated measure to assess harms [3].<br>- Patient & family feedback outcomes are limited in scope & number [1, 3, 31, 33, 40, 47-49, 51, 62, 69, 71]: many recommend or propose the measure of patient & family experience [3, 31, 40, 47, 48, 62, 69, 71], some include measures of patient & family experience [1, 33, 49, 51]. Although limited, feedback was positive & demonstrated preferences for new interventions over previous processes.	Perceived value & appraisal<br>Training & education

For peer review only

		- Reporting the effects of the intervention to those involved: research publication [1, 3, 31-33, 38-43, 45-47, 49-51, 54, 60, 68, 69]; shared on intervention website [3, 38]; results communicated to promote shared learning [54]; shared with consultant panels for improved insight [47].	
	Communal appraisal<br>- Is there communal agreement among staff as to the value of the intervention?<br>- Has the worth of the intervention been evaluated collaboratively in formal or informal groups?	- One study evaluated staff perceptions in a collaborative setting, including focus groups & leadership meetings [69].<br>- Perceived value was evaluated via interviews, audits & questionnaires [3, 40, 41, 46-48, 50, 60, 61, 63, 69].<br>- The importance of individual & group methods in gauging attitudes was described, yet their relative efficiency was not explored [40, 41, 69].<br>- Communal staff appraisal may allow for a greater demonstration of impact than medical record audit data [69].<br>- Many demonstrated communal agreement as to the high level of value that staff attributed to an intervention [3, 40, 41, 47, 48, 50, 60-62, 69].<br>- Assessment of worth related to the intended (& perceived) purpose of interventions [31, 36, 39, 40, 42, 44, 46, 53, 62, 64, 65, 67, 69, 71].<br>- Only one study described negative staff perceptions [40].	Perceived value & appraisal
	Individual appraisal<br>- What is the effect of the intervention on an individuals' workload?<br>- Do individuals value the effect it has on their individual work?	- High value attributed to the intervention by staff, due to positive impact on working practices: reduces negative associations with DNACPR due to its universal application [3] or focus on positive treatment actions [50]; prevents unwanted resuscitation or escalation of care & ensures appropriate level of care [40, 44, 46, 47, 50]; provides a mechanism for forward planning: assessing, communicating & honouring patient preferences [3, 40]; increases staff confidence with, & improves clarity of, patient care plans [40, 44, 47, 50, 62]; improves team working & communication, especially out-of-hours [33, 46, 62]; improves ease & motivation of decision-making processes [44, 47, 60-62].<br>- Perceived & noted increases in workload were a concern for staff. However, it was generally perceived to be a worthwhile investment due to clarity, patient benefit & time saved later on [1, 3, 44, 60, 61, 69].	Workability/functionality<br>Perceived value & appraisal<br>Setting & context
	Reconfiguration<br>- Has appraisal work lead to attempts to improve or modify the intervention?	- Importance of appraisal & reconfiguration emphasised by evidence of its occurrence [31, 32, 36, 39, 41, 42, 46, 47, 51, 61, 62, 69].<br>- Reconfiguration part of the implementation process: adoption of PDSA (plan, do, study, act) cycles [46, 47]; piloting the intervention prior to roll out (with feedback, evaluation & subsequent amendments) [36]; early testing & adaptations [31]; use of sequential role out to amend implementation processes based on gathered insight [69].<br>- Appraisal & feedback led to suggested [69] & actual modifications [31, 36, 39, 46, 47, 61]: refinement of identification or trigger questions [31, 69]; ease of use, layout & clarity [36, 47]; inclusion & clarity of review dates [36, 46]; inclusion of free text boxes [36, 46].<br>- Appraisal led to further study or proposed new areas of study [32, 39, 61, 62, 69]: additional research to determine impact on specific outcomes [39]; feasibility of interventions over longer timeframes [69] & across different settings [32, 61]; studies to address distinct but associated problems identified during the appraisal work (e.g. lack of clinician presence at family meetings) [32, 62, 69].<br>- Appraisal also led to recognition of the need for, & subsequent development of, new patient information materials & services [51, 69], proposals for staff support services [41] & the revision & inclusion of interventions in local & trust policy [42, 47, 61].<br>- In two studies, appraisal demonstrated no desired impact, leading to the proposal or development of alternative interventions [43, 45].	Perceived value & appraisal<br>Input into development<br>Workability/functionality<br>Setting & context<br>Training & education

For peer review only

PRISMA 2009 Checklist

Section/topic	#	Checklist item	Reported on page #
**TITLE**			
Title	1	Identify the report as a systematic review, meta-analysis, or both.	1
**ABSTRACT**			
Structured summary	2	Provide a structured summary including, as applicable: background; objectives; data sources; study eligibility criteria, participants, and interventions; study appraisal and synthesis methods; results; limitations; conclusions and implications of key findings; systematic review registration number.	2
**INTRODUCTION**			
Rationale	3	Describe the rationale for the review in the context of what is already known.	3-4
Objectives	4	Provide an explicit statement of questions being addressed with reference to participants, interventions, comparisons, outcomes, and study design (PICOS).	4
**METHODS**			
Protocol and registration	5	Indicate if a review protocol exists, if and where it can be accessed (e.g., Web address), and, if available, provide registration information including registration number.	N/A for scoping reviews
Eligibility criteria	6	Specify study characteristics (e.g., PICOS, length of follow-up) and report characteristics (e.g., years considered, language, publication status) used as criteria for eligibility, giving rationale.	4-5
Information sources	7	Describe all information sources (e.g., databases with dates of coverage, contact with study authors to identify additional studies) in the search and date last searched.	5-6
Search	8	Present full electronic search strategy for at least one database, including any limits used, such that it could be repeated.	5 & appendix 1
Study selection	9	State the process for selecting studies (i.e., screening, eligibility, included in systematic review, and, if applicable, included in the meta-analysis).	4-6
Data collection process	10	Describe method of data extraction from reports (e.g., piloted forms, independently, in duplicate) and any processes for obtaining and confirming data from investigators.	6
Data items	11	List and define all variables for which data were sought (e.g., PICOS, funding sources) and any assumptions and simplifications made.	6 & appendix 2
Risk of bias in individual studies	12	Describe methods used for assessing risk of bias of individual studies (including specification of whether this was done at the study or outcome level), and how this information is to be used in any data synthesis.	N/A in scoping

For peer review only - http://bmjopen.bmj.com/site/about/guidelines.xhtml

PRISMA 2009 Checklist

			review
Summary measures	13	State the principal summary measures (e.g., risk ratio, difference in means).	7
Synthesis of results	14	Describe the methods of handling data and combining results of studies, if done, including measures of consistency (e.g., I^2) for each meta-analysis.	7

Section/topic	#	Checklist item	Reported on page #
Risk of bias across studies	15	Specify any assessment of risk of bias that may affect the cumulative evidence (e.g., publication bias, selective reporting within studies).	2
Additional analyses	16	Describe methods of additional analyses (e.g., sensitivity or subgroup analyses, meta-regression), if done, indicating which were pre-specified.	N/A
**RESULTS**			
Study selection	17	Give numbers of studies screened, assessed for eligibility, and included in the review, with reasons for exclusions at each stage, ideally with a flow diagram.	7 & figure 1
Study characteristics	18	For each study, present characteristics for which data were extracted (e.g., study size, PICOS, follow-up period) and provide the citations.	8-14
Risk of bias within studies	19	Present data on risk of bias of each study and, if available, any outcome level assessment (see item 12).	15-19 & appendix 4
Results of individual studies	20	For all outcomes considered (benefits or harms), present, for each study: (a) simple summary data for each intervention group (b) effect estimates and confidence intervals, ideally with a forest plot.	15-19 & appendix 4
Synthesis of results	21	Present results of each meta-analysis done, including confidence intervals and measures of consistency.	15-19 & appendix 4
Risk of bias across studies	22	Present results of any assessment of risk of bias across studies (see Item 15).	2
Additional analysis	23	Give results of additional analyses, if done (e.g., sensitivity or subgroup analyses, meta-regression [see Item 16]).	15-19 & appendix 4
**DISCUSSION**			

For peer review only

PRISMA 2009 Checklist

Summary of evidence	24	Summarize the main findings including the strength of evidence for each main outcome; consider their relevance to key groups (e.g., healthcare providers, users, and policy makers).	20-22
Limitations	25	Discuss limitations at study and outcome level (e.g., risk of bias), and at review-level (e.g., incomplete retrieval of identified research, reporting bias).	2 & 20-22
Conclusions	26	Provide a general interpretation of the results in the context of other evidence, and implications for future research.	22
**FUNDING**			
Funding	27	Describe sources of funding for the systematic review and other support (e.g., supply of data); role of funders for the systematic review.	23

From: Moher D, Liberati A, Tetzlaff J, Altman DG, The PRISMA Group (2009). Preferred Reporting Items for Systematic Reviews and Meta-Analyses: The PRISMA Statement. PLoS Med 6(6): e1000097. doi:10.1371/journal.pmed1000097

For more information, visit: **www.prisma-statement.org**.

Page 2 of 2

For peer review only
